# Streamlined Synthesis and Structure–Activity Relationship Analysis of 2‐Amidothiophene‐3‐Carboxamides Targeting Influenza Polymerase PA‐PB1 Heterodimerization

**DOI:** 10.1002/cmdc.70314

**Published:** 2026-05-22

**Authors:** Tommaso Felicetti, Alessia Zago, Andrea Astolfi, Giuseppe Manfroni, Stefano Sabatini, Maria Letizia Barreca, Oriana Tabarrini, Arianna Loregian, Serena Massari

**Affiliations:** ^1^ Department of Pharmaceutical Sciences University of Perugia Perugia Italy; ^2^ Department of Molecular Medicine University of Padua Padua Italy; ^3^ Microbiology and Virology Unit Padua University Hospital Padua Italy

**Keywords:** antiviral agents, Gewald reaction, influenza virus, PA‐PB1, polymerase, protein–protein interactions

## Abstract

Influenza viruses remain a major global health threat due to their rapid evolution and ability to evade current therapies. Among viral targets, the PA–PB1 interface of the RNA polymerase complex has emerged as an attractive site for small‐molecule inhibition. Based on compound **1**, a previously identified PA–PB1 interaction inhibitor featuring a cycloheptathiophene‐3‐carboxamide scaffold, we designed and synthesized a new series of derivatives to investigate the role of the cycloheptyl moiety in antiviral activity and water solubility. In parallel, we developed an improved three‐step synthetic route to access 2‐amidothiophene‐3‐carboxamide analogs more efficiently. The new derivatives (**2–16**) provided valuable structure–activity relationship insights, highlighting how modifications at C‐5 influence both anti‐influenza potency and solubility. Among them, the C‐5 phenyl analog **9** displayed the strongest antiviral activity, achieving sub‐micromolar EC_50_ values (0.19–1.11 µM) across a panel of influenza strains, along with a CC_50_ value > 100 µM. Notably, the C‐5 methyl analog **5** showed the greatest enhancement in aqueous solubility (75.2 µM) while maintaining low‐micromolar potency (EC_50_ of 2 µM) and no significant toxicity (CC_50_ > 100 µM). Despite structural divergence from the starting hit **1**, both compounds preserved the PA–PB1 interaction inhibition mechanism, as demonstrated by enzyme‐linked immunosorbent assay (ELISA) and supported by docking studies within the PA_C_ cavity.

## Graphical abstract

Development of a three‐step synthesis and replacement of the cycloheptyl moiety in cycloheptathiophene‐3‐carboxamide derivatives revealed how thiophene C‐5 substitutions modulate PA–PB1 inhibition, antiviral potency, and water solubility. The phenyl analog **9** delivered sub‐µM potency across multiple IV strains, while the methyl analog **5** achieved the highest aqueous solubility (75 µM), retaining low µM activity.

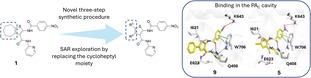

## 1 Introduction

**FIGURE 1:**
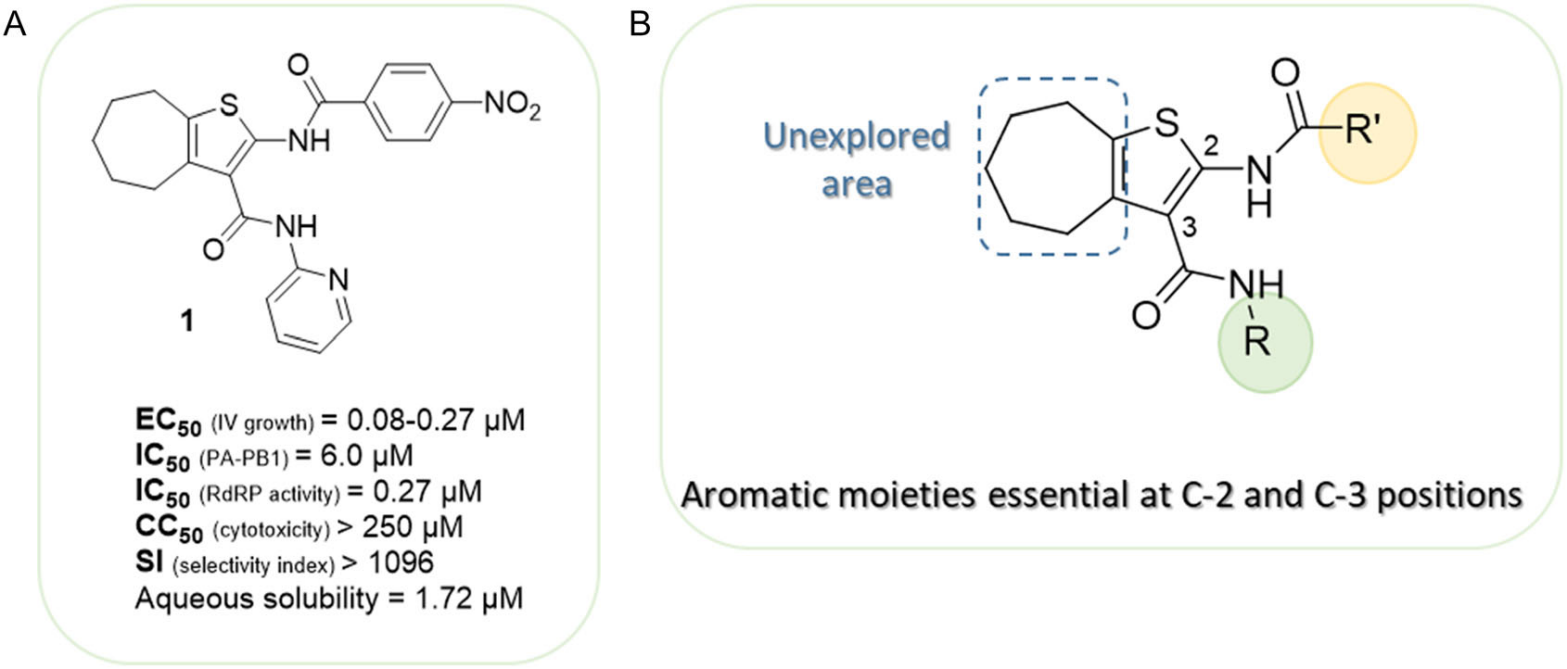
(A) Chemical structure and activities of the hit compound **1** [28, 30]. The EC_50_ ranges were determined using a panel of IV strains. The PA–PB1 interaction inhibition was determined by an enzyme‐linked immunosorbent assay (ELISA)‐based assay. The RdRP inhibition was determined by a minireplicon assay. The aqueous solubility refers to thermodynamic water solubility at 24 h. (B) Representation of the cHTC general structure and SAR insights at the C‐2 and C‐3 positions previously determined [29].

Influenza virus (IV) is a segmented, single‐stranded, negative‐sense RNA virus that belongs to the *Orthomyxoviridae* family. The classification of IVs is determined by their structural differences and is divided into four genera: A, B, C, and D, with types A and B being the most clinically relevant for humans [1, 2]. The viral surface of the last two is studded with two major glycoproteins, hemagglutinin (HA) and neuraminidase (NA), which are critical for the virus life cycle and the primary targets of the host immune response [3]. IV exhibits remarkable genetic variability, driven by point mutations (antigenic drift) and the exchange of entire gene segments between different viral strains (antigenic shift or reassortment) [4]. This variability enables IV to evade pre‐existing host immunity, necessitating the annual reformulation of vaccines [5]. Influenza A viruses (IAV), which possess a vast animal reservoir in wild aquatic birds and the capacity for interspecies transmission, are the primary drivers of pandemics due to antigenic shift [6]. In contrast, influenza B viruses (IBV) predominantly circulate among humans and seals, resulting in more limited, albeit frequently severe, seasonal epidemics [7]. Typically, infection results in an acute respiratory illness that can range from mild to life‐threatening, particularly in high‐risk populations. This phenomenon can result in severe complications, including viral or secondary bacterial pneumonia [8]. However, epidemiological data underscore the profound impact of seasonal influenza on global public health. According to the World Health Organization (WHO), approximately one billion cases of seasonal influenza are reported annually, including three to five million cases of severe illness and 290,000–650,000 deaths [9]. Vaccination remains the most effective method for controlling seasonal influenza's morbidity and mortality [10]. However, a primary limitation is the suboptimal and variable effectiveness of current vaccines, which is highly dependent on the antigenic match between the vaccine strains and the rapidly circulating mutating viruses, thus requiring reformulation every year [11]. Specific antiviral agents have been identified as a critical measure for the management of influenza, due to their ability to target pivotal viral components, such as NA and RNA‐dependent RNA polymerase (RdRP) [12]. However, the efficacy of these agents is frequently contingent upon their administration within 48 h of symptom onset. This limitation is further aggravated by the continuous emergence of drug‐resistant viral strains, underscoring the strong need for the development of novel therapeutic interventions [12, 13]. In this regard, RdRP is widely regarded as a primary target for the development of antiviral agents. In the case of IV, the RdRP possesses a unique heterotrimeric structure that performs three different functions: endonuclease (PA subunit), polymerase *stricto sensu* (PB1), and cap‐snatching (PB2 subunit) [14, 15, 16]. It is noteworthy that two approved drugs target two of these subunits: baloxavir marboxil (BXM) [17, 18, 19], an endonuclease inhibitor, and favipiravir (FPV) [20], which inhibits polymerase function. A PB2 inhibitor is pimodivir, whose clinical trials were discontinued in recent years [12]. However, to date, no molecule capable of inhibiting interactions between IV RdRP subunits has reached advanced stages of clinical trials. Nevertheless, the prevention of viral growth through the use of protein–protein interaction (PPI) inhibitors of IV RdRP subunits may constitute an excellent strategy, with the potential to overcome the significant challenge of drug resistance, thanks to the low possibility of mutating both subunits concurrently [21, 22]. On this line, the PA–PB1 binding interface (pdb: 2ZNL and 3CM8) [23, 24] has attracted considerable research interest in the development of novel anti‐IV agents. This is attributable to the fact that only a few highly conserved residues drive the PA‐PB1 binding, suggesting the possibility of using small molecules as PPI inhibitors [21]. For many years, our research group has been engaged in the development of two classes of PA‐PB1 inhibitors: triazolopyrimidine (TZP) [25, 26] and cycloheptathiophene‐3‐carboxamide (cHTC) [27, 28, 29] derivatives. Among them, the cHTC derivatives exhibited optimal efficacy, with some compounds demonstrating potent anti‐IV activity in cellular contexts with nanomolar EC_50_ values and excellent selectivity indexes (compound **1** depicted in Figure 1A as a representative). This potency suggested the high potential of inhibiting the PA–PB1 interaction, a mechanism that was validated through orthogonal assays. In addition, as observed for compound **1**, this peculiar mechanism also translated into a high barrier to drug resistance and in promising synergistic activity with approved NA inhibitor drugs both in cell culture and in ex vivo infected models [30, 31]. However, due to suboptimal PK properties, particularly a reduced solubility (thermodynamic water solubility of **1** = 1.72 µM) [29], associated with this series, none of them has ever been advanced to studies in animal models. Consequently, the efficacy of PA–PB1 interaction inhibitors remains to be validated in in vivo models. The most recent medicinal chemistry optimization of **1**, which exclusively concerned the modification of the substituents of the aromatic moieties at C‐2 and C‐3, led to an increase in potency but not to a sufficient increase in water solubility, despite the introduction of more polar groups capable of forming hydrogen bonds [29]. However, although the structure–activity relationship (SAR) indicated that an aromatic substituent was essential at the C‐2 and C‐3 positions, and its replacement with an aliphatic portion or its removal was detrimental for the activity, information from the “cycloheptyl” part of the molecule was limited (Figure 1B). Consequently, there is potential for further exploration of the cycloheptyl area, particularly with respect to both SAR and, given the lipophilic nature of this saturated ring, water solubility.

**SCHEME 1:**
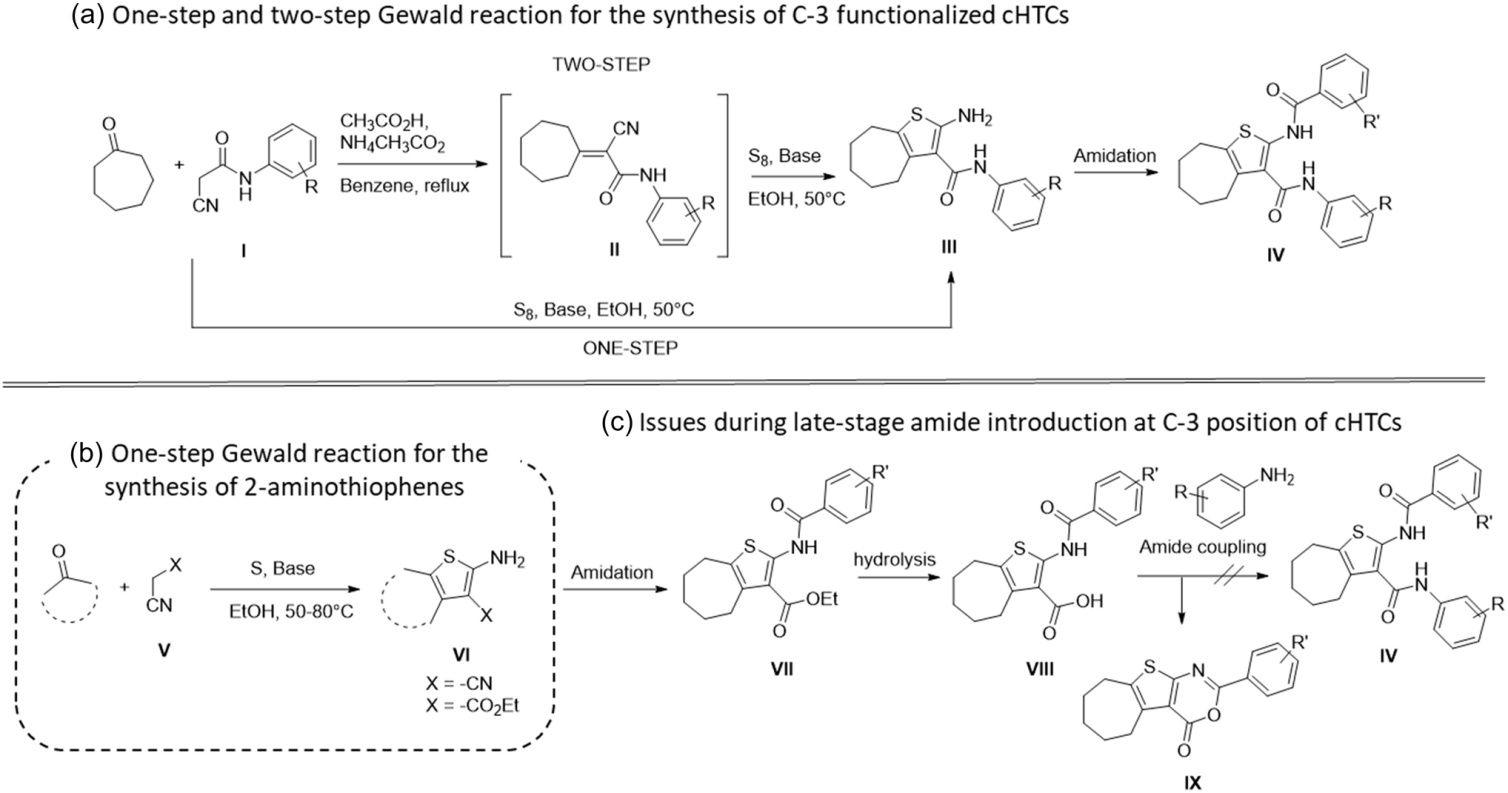
(a) Representation of the one‐step and two‐step Gewald reaction for the synthesis of previously reported C‐3 functionalized cHTC [28]. (b) Representation of the one‐step Gewald reaction for the synthesis of 2‐aminothiophenes. (c) Representation of the formation of the cyclized side product during the synthesis of cHTCs during the attempts to replace the ester at the C‐3 position with a substituted amide [28].

Another issue with this series of compounds concerns the synthetic procedure. Indeed, the synthesis of cHTCs via the one‐pot three‐component Gewald reaction (Scheme 1a) is challenging, owing to the necessity of using a substituted cyanoacetamide (**I**) that is already bearing the final C‐3 substituent of cHTCs (**IV**). This results in a low yield, likely due to the reduced reactivity of the substituted cyanoacetamide (**I**) compared to the traditional malononitrile or ethyl cyanoacetate (**V**) employed in the original reaction, furnishing 2‐aminothiphenes **VI** (Scheme 1b) [32]. A two‐step Gewald reaction is indeed required (Scheme 1a), entailing first the isolation of the Knoevenagel intermediate **II**, which is obtained by reacting the cycloheptanone and the substituted cyanoacetamide **I**. Subsequently, intermediate **II**, which is poorly stable, is subjected to a reaction with sulfur in the presence of a base, culminating in the synthesis of intermediate **III** [28]. A notable drawback of this two‐step Gewald reaction is its time‐consuming nature, with an approximate duration of 12 h, the requirement of elevated temperatures, and the utilization of benzene as a solvent [28]. Moreover, the yield of the two‐step procedure is approximately 50%, and chromatographic purification is always required [28].

Although ethyl cycloheptathiophene‐3‐carboxylate (general structure **VI**, Scheme 1b) is more readily available, it is not suitable for the synthesis of C‐3 amide‐substituted cHTCs (**IV**, Scheme 1c). Indeed, as previously reported in 2017 by us, the functionalization of **VI** at the C‐2 position (to **VII**), hydrolysis (to **VIII**), and amide coupling with aromatic amines led exclusively to the cyclized side product **IX** [28].

**FIGURE 2:**
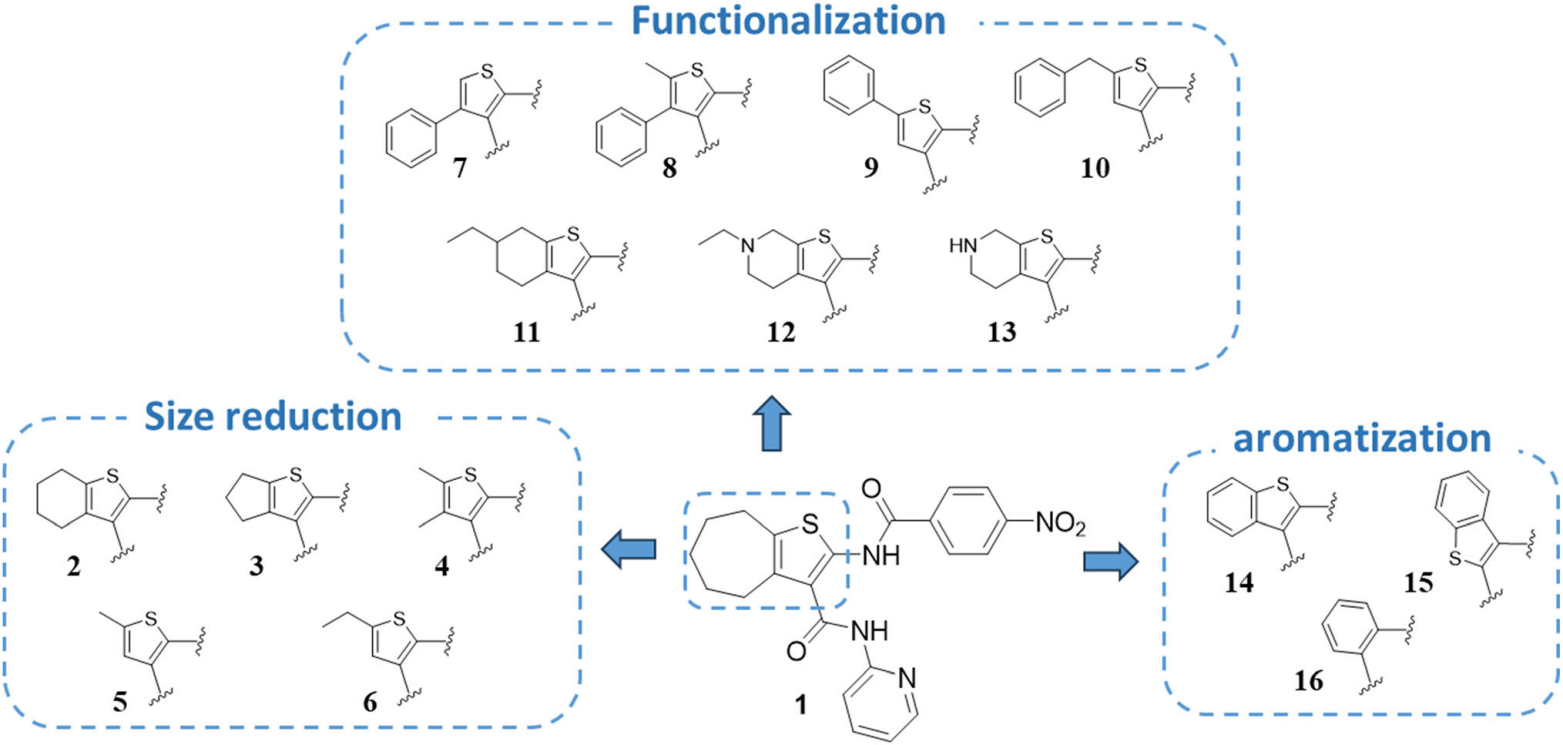
Chemical structures of the designed analogs **2–16** starting from the hit compound **1**.

In light of these considerations, the present work aims at optimizing a synthetic procedure for the synthesis of C‐3 substituted 2‐amidothiophene derivatives. Consequently, this optimized procedure facilitated the synthesis of analogs of compound **1**, which were designed to investigate the SAR and elucidate the role of the cycloheptathiophene in the molecule's water solubility. The modifications applied to the cycloheptathiophene core of **1** encompassed size reduction, functionalization, and aromatization (compounds **2–16**, Figure 2).

## 2 Results and Discussion

### 2.1 Optimization of the Synthesis of C‐3‐Substituted 2‐Amidothiophene Derivatives

**SCHEME 2:**
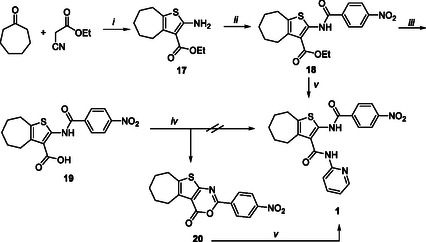
Reagents and conditions: (*i*) sulfur, morpholine, dry EtOH, 50°C, 10 h, 89%; (*ii*) 4‐nitrobenzoyl chloride, dry pyridine, rt, 60 min, 97%; (*iii*) LiOH, H_2_O, THF, rt, 12 h, 65%; (*iv*) 2‐aminopyridine, TBTU, DIPEA, dry DMSO, rt, 60 min, 88%; (*v*) 2‐aminopyridine, 1 M LiHMDS in THF, dry THF, rt, 60 min, 81% (from **20**), 74% (from **18**).

In light of the limitations of the two‐step Gewald reaction, our research was initiated with an attempt to reprepare derivative **1** by the one‐pot Gewald reaction, employing ethyl cyanoacetate as the starting material (Scheme 2). Consequently, the ethyl 2‐amino‐cycloheptathiophene‐3‐carboxylate **17** [33] was obtained through a reaction between cycloheptanone, ethyl cyanoacetate, and sulfur in the presence of morpholine, with dry EtOH serving as the solvent. Subsequently, the amino group of **17** was amidated with 4‐nitrobenzoyl chloride in dry pyridine, resulting in the formation of derivative **18**. This derivative was then subjected to mild basic hydrolysis with LiOH, yielding the acid derivative **19**. Subsequently, we reacted compound **19** with 2‐aminopyridine under the same conditions previously reported by us (BOP and collidine in dry DMSO) [28], which resulted in the formation of the cyclized side product. As expected, the cyclized derivative **20** was the sole product of the reaction. Even replacing BOP with different coupling agents, such as TBTU or HOBt/EDCI, and replacing collidine with different bases, such as Et_3_N and DIPEA, the cyclized derivative **20** was the only product observed.

At this stage, an attempt was made to activate 2‐aminopyridine using strong bases, such as NaH, *t*‐BuOK, or LiHMDS. Subsequently, compound **20** was added as an electrophile to the reaction. In the presence of NaH or *t*‐BuOK activation, no observable conversion of compound **20** was detected. Conversely, the activation of 2‐aminopyridine with LiHMDS resulted in a complete conversion of **20**, yielding the desired compound **1** after a 30 min reaction and achieving an 81% yield. Inspired by these findings, we sought to determine whether the reactivity of the activated 2‐aminopyridine could be utilized directly on the ester intermediate **18**. With our delight, the reaction of **18** with 2‐aminopyrimidine in the presence of LiHMDS in dry THF yielded the compound **1** in a 74% yield after crystallization. This reaction was completed within 60 min. It is noteworthy that the synthetic procedure was repeated on a gram scale with no substantial alterations in time and yields. These findings represent a significant advancement in the synthesis of substituted 2‐amidothiophene‐3‐carboxamide compounds, which can be obtained in three steps and with satisfactory overall yields. Indeed, through this novel synthetic approach, compound **1** was successfully synthesized with an encouraging 64% overall yield. This outcome significantly surpasses the prior reported yield of 23%, which was achieved through a more complex six‐step synthetic process involving the preparation of substituted cyanoacetamide (three steps), the two‐step Gewald reaction, and the final C‐2 amide coupling. Moreover, this furnished us with a considerable advantage in synthesizing analogs of **1** designed to replace the cycloheptathiophene moiety.

**SCHEME 3:**
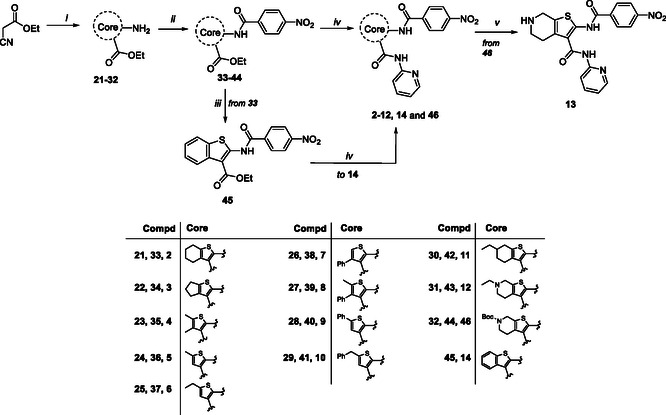
Reagents and conditions: (*i*) sulfur, morpholine, dry EtOH, 50°C, 2–24 h, 55%–95%; (*ii*) 4‐nitrobenzoyl chloride, dry pyridine, rt, 30 min‐4 h, 60–100%; (*iii*) DDQ, benzene, reflux, 10 h, 60%; (i*v*) 2‐aminopyridine, dry LiHMDS 1 M, dry THF, rt, 30 min‐14 h, 63%–95%; (*v*) TFA, dry CH_2_Cl_2_, 0°C, 3h, 81%.

Accordingly, we initiated the synthesis of compounds **2–16** through the one‐step Gewald reaction (Scheme 3). This reaction requires the presence of ethyl cyanoacetate, the appropriate ketone or aldehyde, and sulfur. The syntheses of these intermediates have already been reported in the literature using various procedures, as indicated below for each intermediate. However, a single procedure was selected for the synthesis of all intermediates, which involved the use of morpholine as the base, dry EtOH as the solvent, and a temperature of 50°C. In this manner, according to the reactivity of the starting ketone or aldehyde, we obtained derivatives **21** [34], **22** [35], **23** [36], **24** [37], **25** [36], **26** [38], **27** [39], **28** [40], **29** [41], **30** [35], **31** [42], and **32** [43], in yields ranging from 55 to 95%. Subsequent C‐2 amidation was achieved through the reaction of intermediates **21–32** with 4‐nitrobenzoyl chloride in dry pyridine, resulting in compounds **33–44** (60–100% yields). Intermediate **33** underwent oxidation with 2,3‐dichloro‐5,6‐dicyano‐1,4‐benzoquinone (DDQ) in benzene at reflux, yielding aromatic derivative **45**. Subsequently, C‐3 amidation of compounds **33–45** with 2‐aminopyridine in the presence of 1 M LiHMDS in THF afforded the target compounds **2–12**, **14**, and intermediate **46** in yields ranging from 63 to 95% after a purification step. Finally, the *N*‐Boc group of compound **46** was removed using a trifluoroacetic acid (TFA) solution in dry CH_2_Cl_2_, yielding the target compound **13**.

**SCHEME 4:**
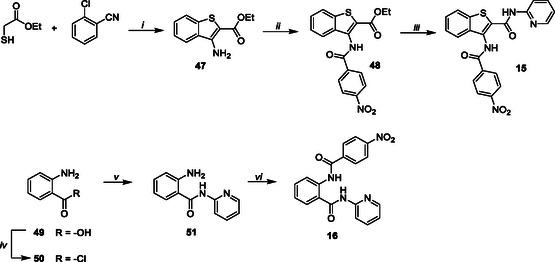
Reagents and conditions: (*i*) KOH, DMF, 80°C, 12 h, 67%; (*ii*) 4‐nitrobenzoyl chloride, dry pyridine, rt, 6 h, 66%; (*iii*) 2‐aminopyridine, 1 M LiHMDS in dry THF, dry THF, rt, 12 h, 86%; (*iv*) SOCl_2_, reflux, 4 h, 100%; (*v*) 2‐aminopyridine, dry pyridine, rt, 3 h, 61%; (*vi*) 4‐nitro benzoyl chloride, dry pyridine, rt, 5 h, 85%.

The target compounds **15** and **16** were synthesized, as outlined in Scheme 4, employing 2‐(ethoxycarbonyl)‐3‐aminobenzo[*b*]thiophene **47** [33] and anthranilic acid **49**, respectively, as the starting materials. Compound **47** [33], which was obtained from the condensation of ethyl thioglycolate and 2‐chlorobenzonitrile in DMF with KOH, was substituted at the C‐3 position with 4‐nitrobenzoyl chloride in dry pyridine to give the intermediate **48**. C‐2 amidation of **48** was performed using 2‐aminopyridine in dry THF with 1 M LiHMDS in THF, affording the desired compound **15** in 86% yield. Conversely, the chlorination of anthranilic acid **49** with SOCl_2_ at reflux yielded compound **50**, which was then coupled with 2‐aminopyridine to give intermediate **51** [44]. Finally, a reaction with 4‐nitrobenzoyl chloride in dry pyridine produced the target compound **16**.

### 2.2 Design and Anti‐IAV Activity of 2‐Amidothiophene‐3‐Carboxamide Derivatives

The design of the new derivatives was based on the starting hit **1**, which is our best characterized cHTC compound in terms of its mechanism of action and anti‐IV activity in a cellular context and ex vivo models [28, 30, 31]. The design involved retaining the C‐2 4‐nitrophenyl and C‐3 2‐pyridine rings of **1**, while extensively modifying the cycloheptyl portion. This investigation was undertaken to explore the SAR and to elucidate the role of this structural element in determining the molecule's water solubility.

The modifications applied to the cycloheptathiophene core of **1** entailed a reduction in size, functionalization, and aromatization. This approach led to the synthesis of compounds **2–16** (see Figure 2 and Table 1). Specifically, the cycloheptathiophene ring of **1** was reduced to form the tetrahydrobenzothiophene compound **2** and the cyclopentathiophene compound **3**. Further size reduction was then studied in compounds **4–6**, wherein the cycloheptane ring was removed, and the thiophene ring was substituted with 4,5‐dimethyl‐, 5‐methyl‐, and 5‐ethyl groups. The cycloheptane ring was also removed in compounds **7–10**, for which the thiophene ring functionalization was examined by introducing bulkier substituents, such as 4‐phenyl‐5‐methyl‐, 4‐phenyl‐, 5‐phenyl‐, and 5‐phenylmethyl groups, respectively. The presence of a six‐member ring fused to the thiophene was further investigated in compounds **11–13**, which are characterized by a substituted or unsubstituted six‐member (hetero)aliphatic ring. Finally, the aromatization of the core was studied in compounds **14–16**. This resulted in compound **14**, which is the close aromatic analog of compound **2**; in compound **15**, which has the C‐2 and C‐3 substituents inverted with respect to **14**; and in compound **16**, which has the cycloheptathiophene ring of hit compound **1** replaced with a benzene ring.

**TABLE 1:** Structure, biological activity, and aqueous solubility of derivatives **1–16**.

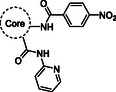					
**Compd**	**Core**	**Anti‐IAV (PRA)** **EC**_**50**_, **µM** a	**Cytotoxicity** **(MTT Assay)** **CC**_**50**_, **µM** b	**SI** c	**Thermodynamic solubility (µM),** **(CLogP)** d
**1**	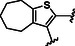	0.26 ± 0.13	>250	>1096	<10 (4.93)
**2**	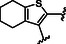	0.39 ± 0.05	>100	>256	<10 (4.37)
**3**	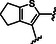	>25	>100	—	<10 (3.81)
**4**	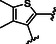	0.27 ± 0.07	>100	>370	22.4 (3.41)
**5**	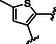	2.00 ± 0.28	>100	>50	75.2 (3.30)
**6**	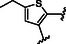	1.10 ± 0.34	>100	>91	25.2 (3.83)
**7**	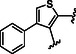	9.2 ± 1.8	> 100	>27	<10 (4.23)
**8**	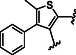	>50	>100	—	<10 (4.43)
**9**	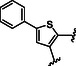	0.36 ± 0.12	>100	>278	<10 (4.89)
**10**	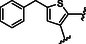	28.0 ± 9.0	>100	>9	<10 (4.87)
**11**	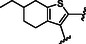	0.27 ± 0.03	>100	>370	12.00 (5.40)
**12**	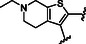	>50	>100	—	<10 (3.23)
**13**	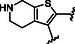	>100	>100	—	27.7 (2.25)
**14**	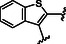	>10	24.5 ± 4.9	—	<10 (4.18)
**15**	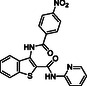	>100	>100	—	<10 (4.18)
**16**	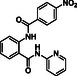	>100	>100	—	<10 (3.07)
**RBV**		8.7 ± 0.8	>100	>12	—
**FPV**		7.7 ± 0.4	>100	>13	—

a Activity of the compounds in PRA with the IAV/PR/8/34 strain. The EC_50_ value represents the compound concentration that inhibits 50% of plaque formation.

b Cytotoxicity of the compounds in MTT assays. The CC_50_ value represents the compound concentration that causes a 50% decrease in cell viability. All the reported values represent the means ± SD of data derived from at least three independent experiments (*n* = 3) in duplicate.

c SI, selectivity index (SI = CC_50_/EC_50_).

d CLogP calculated by ChemDraw v.25.5.0. RBV, ribavirin; FPV, favipiravir.

The anti‐IAV activity of all the synthesized compounds (**2–16**) was evaluated in Madin–Darby canine kidney (MDCK) cells infected with the IAV/PR/8/34 (PR8) strain using plaque reduction assay (PRA), with ribavirin (RBV) and FPV serving as positive controls (Table 1). The compounds were also assessed for potential toxicity using an MTT assay in MDCK cells to confirm that the observed anti‐IAV activity was not due to toxic effects on the cells. Concurrently, the thermodynamic solubility of all the compounds was evaluated using the shake flask method.

As shown in Table 1, the reduction in size of cycloheptane to cyclohexane, but not to cyclopentane, was tolerated, as evidenced by compound **2**, which showed an EC_50_ of 0.39 µM. However, neither of these derivatives exhibited an increase in water solubility despite the progressive reduction in lipophilicity. The replacement of the cycloheptane moiety with 4,5‐dimethyl groups yielded analogous results, with compound **4** demonstrating potent anti‐IAV activity (EC_50_ = 0.27 μM). In addition, compounds **5** and **6**, which possessed a C‐5 methyl or ethyl group, exhibited significant anti‐IAV activity, with EC_50_ values of 2.00 and 1.10 µM, respectively. All three derivatives demonstrated a substantial decrease in lipophilicity and thus an increased water solubility. Specifically, the C‐5 methyl derivative **5** showed a significant improvement in terms of thermodynamic solubility, reaching an encouraging value of 75.2 µM.

From a SAR perspective, the presence of an additional small substituent at C‐4, along with the one at C‐5, ensured high anti‐IAV activity, as demonstrated by compound **4**. However, the presence of a bulkier substituent, such as a phenyl group, at C‐4 significantly reduced antiviral activity (compound **7**, EC_50_ **=** 9.2 µM) and led to a complete loss of activity when a methyl group is present at C‐5 (compound **8**, EC_50_ **>** 50 µM). Notably, the relocation of the phenyl ring from C‐5 to C‐4 resulted in the restoration of the anti‐IAV potency (compound **9**, EC_50_ **=** 0.36 µM). This observation indicates that a bulky substituent is well tolerated at the C‐5, while at the C‐4 position, there is no room for excessively large groups. Increasing the flexibility of the C‐5 substituent by adding a methylene bridge between the thiophene and the phenyl negatively impacted on anti‐IAV activity; in fact, compound **10** showed an EC_50_ of 28 µM. As expected, the increase in the size of the lipophilic substituent in compounds **7–10** resulted in elevated lipophilicity, consequently leading to poor aqueous solubility.

Replacing the cycloheptane with a six‐membered (hetero)aliphatic ring (compounds **11–13**) produced different results. Compounds **12** and **13** were devoid of anti‐IAV activity. However, the presence of an ethyl group in the C‐6 position of the cyclohexyl moiety in compound **11** was found to confer potent anti‐IAV activity (EC_50_ = 0.27 μM). Interestingly, we also observed a slight increase in the water solubility of compound **11**, just above the detection limit of 10 µM, despite an increase in lipophilicity compared to derivative **2**. Conversely, replacing the carbon at C‐6 of compound **11** with a nitrogen atom resulted in compound **12**, which exhibited reduced lipophilicity but not enhanced water solubility. A further decrease in lipophilicity was obtained by deleting the ethyl group from **12**, which in this case correlated with a slight increase in water solubility, reaching the value of 27.7 µM (compound **13**).

When we attempted to aromatize the aliphatic moiety bound to the thiophene (compound **14**), we observed a drastic increase in toxicity. In contrast, the inversion of the thiophene while maintaining the aromatic moiety, or the deletion of the thiophene, resulted in compounds **15** and **16**, which showed neither toxicity nor anti‐IAV activity nor enhanced water solubility.

In summary, the data demonstrate that the cycloheptyl present in **1** is not essential for the anti‐IAV activity and is not directly responsible for the poor solubility of compounds belonging to this series. Indeed, the findings of this study indicate that the water solubility of these analogs is not solely dependent on their lipophilicity, as an increase in polarity is frequently not accompanied by an increase in solubility. In terms of anti‐IV activity, the cycloheptyl can be simply replaced with two highly similar moieties: cyclohexyl (compound **2**) or ethyl cyclohexyl (compound **11**). These substitutions do not result in substantial changes in potency. An alternative option is to “cleave” this lipophilic cycloheptyl portion, leaving only two methyl groups at the C‐4 and C‐5 positions; a change that did not reduce the anti‐IV activity. Unfortunately, these modifications, even in the case of the dimethyl derivative (compound **4**), do not lead to a significant increase in solubility. The most compelling SAR data is exhibited by compound **9**, which contains a phenyl at the C‐5 position and exhibits antiviral activity comparable to that of **1**, suggesting that an aromatic substituent at this position is well‐tolerated. Conversely, if the phenyl is moved to C‐4, the compound exhibits a complete loss of activity, suggesting a clear SAR for these two positions. However, it appears that obtaining a compound with a sub‐micromolar EC_50_ and promising aqueous solubility simply by replacing the cycloheptyl with other groups is not feasible. This is particularly significant because any effort to introduce additional polar or heterocyclic structures invariably leads to compounds that are devoid of anti‐IV activity. However, in this direction, compound **5** has emerged as a particularly promising candidate, exhibiting promising solubility values (75.2 μM) without compromising its anti‐IAV activity, as it maintains an EC_50_ of 2 μM. This data is of great interest for future design, particularly in light of the recent findings concerning the modification of the substituents at the C‐2 and C‐3 positions of **1**. Indeed, some potent derivatives have exhibited a notable enhancement in both anti‐IAV activity and aqueous solubility [29]. Therefore, the combination of the substituents of these cHTC analogs and the replacement of the cycloheptatiophene with the methyl thiophene of **5** may lead to a highly effective compromise in terms of anti‐IV activity and water solubility.

### 2.3 Further Biological Evaluation of Compounds 5 and 9

At this stage, we were interested in determining whether the modifications made to the cycloheptyl of **1** could have impacted the mechanism of action of the new analogs. Consequently, we selected compounds **5** and **9** to evaluate their ability to inhibit PA‐PB1 heterodimerization by enzyme‐linked immunosorbent assay (ELISA) using the PB1_1–15_‐Tat peptide as a positive control. The selection of these two compounds was guided by the following considerations. Compound **5** exhibited a substantial enhancement in aqueous solubility, demonstrating a significant improvement over all other compounds. Additionally, it is the compound in which the lipophilic substituent was minimized the most, with a decrease from the cycloheptyl of **1** to the methyl of **5**. On the other hand, compound **9** exhibited remarkable activity, comparable to compounds **4** and **11**, exhibiting a sub‐micromolar EC_50_ and an excellent selectivity index. However, the aqueous solubility of all three compounds was found to be poor, with results comparable to those of the starting compound **1**. The decision to conduct further studies using compound **9** was based on its structural differences compared to the starting compound **1**. Notably, the presence of the phenyl at position 5 of the thiophene nucleus represents a significant novelty in this series of derivatives, suggesting potential for further targeted modifications. Therefore, we have decided to evaluate whether this modification alters the mechanism of action of compound **9**.

Both compounds retained the capacity to disrupt the PA–PB1 interaction, with IC_50_ values of 18.7 ± 6.1 µM for compound **5** and 15.5 ± 3.1 µM for compound **9**. This finding suggests that the modification of the cycloheptyl group of **1** (IC_50_ = 6.0 µM) did not alter the mechanism of action of these compounds and confirms that the cycloheptyl moiety is not essential for PA‐PB1 inhibition, in accordance with the anti‐IAV activity.

To provide further confirmation of the mechanism of action, an additional investigation was conducted to determine whether compound **9**, the most potent among the two considered analogs, could inhibit IAV RdRP activity using a minireplicon assay in human embryonic kidney (HEK) 293T cells (see Table 2). Notably, the compound demonstrated a robust inhibitory effect on RdRP functions, exhibiting an IC_50_ value of 36 nM without any observed cellular toxicity in HEK 293T cells at the tested concentrations (CC_50_ > 50 µM). Finally, the anti‐IV activity of compound **9** was evaluated against a panel of human IAV and IBV strains (see Table 2). Of note, it maintained the antiviral activity in the sub‐micromolar range against all the tested strains, including an oseltamivir‐resistant IAV strain, thus demonstrating a broad‐spectrum anti‐IV action. These findings suggest that the observed inhibition of PA–PB1 interaction, as determined by ELISA‐based assay, correlates with a significant reduction of IV RNA polymerase activity and a robust anti‐IV effect.

**TABLE 2:** Activity of compound 9 against a panel of IAV and IBV strains, and against viral RNA polymerase.

Virus strain	**Compound** **PRA (EC**_**50**_ **, µM)** a	
	**9**	**RBV**
**A/PR/8/34 (H1N1)**	0.36 ± 0.12	7.4 ± 0.7
**A/Parma/24/09 (H1N1)** **(oseltamivir‐resistant)**	0.31 ± 0.04	22.0 ± 1.4
**A/Wisconsin/67/05 (H3N2)**	0.19 ± 0.05	5.8 ± 1.1
**B/Lee/40 (ancestral)**	1.11 ± 0.15	19.5 ± 3.5
**B/Malaysia/2506/04**	0.72 ± 0.18	10.8 ± 2.5
**Minireplicon Assay** **(IC**_**50**_ **, µM)** b	0.036 ± 0.006	21.7 ± 7.3
**Cytotoxicity (MTT Assay)** **(CC**_**50,**_ **µM)** c	>50	>50

a The EC_50_ value represents the compound concentration that inhibits 50% of viral plaque formation in MDCK cells.

b The IC_50_ value represents the compound concentration that reduces by 50% the activity of IAV RNA polymerase in HEK 293T cells.

c The CC_50_ value represents the compound concentration that causes a decrease of HEK 293T cells viability of 50%. All data shown represent the means ± SD of data derived from three independent experiments (*n* = 3) in duplicate.

### 2.4 Docking Studies

Despite the significant structural differences from the cycloheptathiophene derivative **1**, compounds **5** and **9** retained the ability to inhibit PA‐PB1 subunit heterodimerization (IC_50_ values of 18.7 and 15.5 µM, respectively). This prompted us to investigate the binding of both compounds to the PA_C_ cavity, particularly in comparison with the original hit compound **1** (IC_50_ = 6.0 µM, EC_50_ = 0.26 µM) and compound **7**. The latter was included since the shift of the phenyl group from C‐5 in compound **9** to C‐4 in compound **7** makes the molecule less effective in inhibiting viral replication (EC_50_ = 9.25 µM). For comparative purposes, we also evaluated the ability of compound **7** to displace the PA–PB1 interaction, finding it to be much less active (IC_50_ of 80.5 ± 7.8 µM).

Thanks to the availability of two crystallographic structures of the PA_C_–PB1_N_ complex [23, 24], previous computational studies have explored the conformational plasticity of this PPI region and the binding modes of PA_C_–PB1_N_ interaction inhibitors within the same pocket [21, 45]. These studies have enabled the identification of three hydrophobic subpockets (HSP) that ligands can exploit to engage with PA_C_: (i) HSP‐1, which is defined by W706 and F411; (ii) HSP‐2, which is formed by F710 and L666; and (iii) HSP‐3, which is lined by L640, V636, M595, and W619.

For previously reported cHTC compounds, the most common binding mode suggested by docking studies involved the cycloheptathiophene core being placed within HSP‐1. Meanwhile, the C‐2 aromatic substituent occupied HSP‐2, and the C‐3 aromatic substituent extended toward an additional central hydrophobic region, which was mainly shaped by the aliphatic chain of E623 and the protein backbone.

**FIGURE 3:**
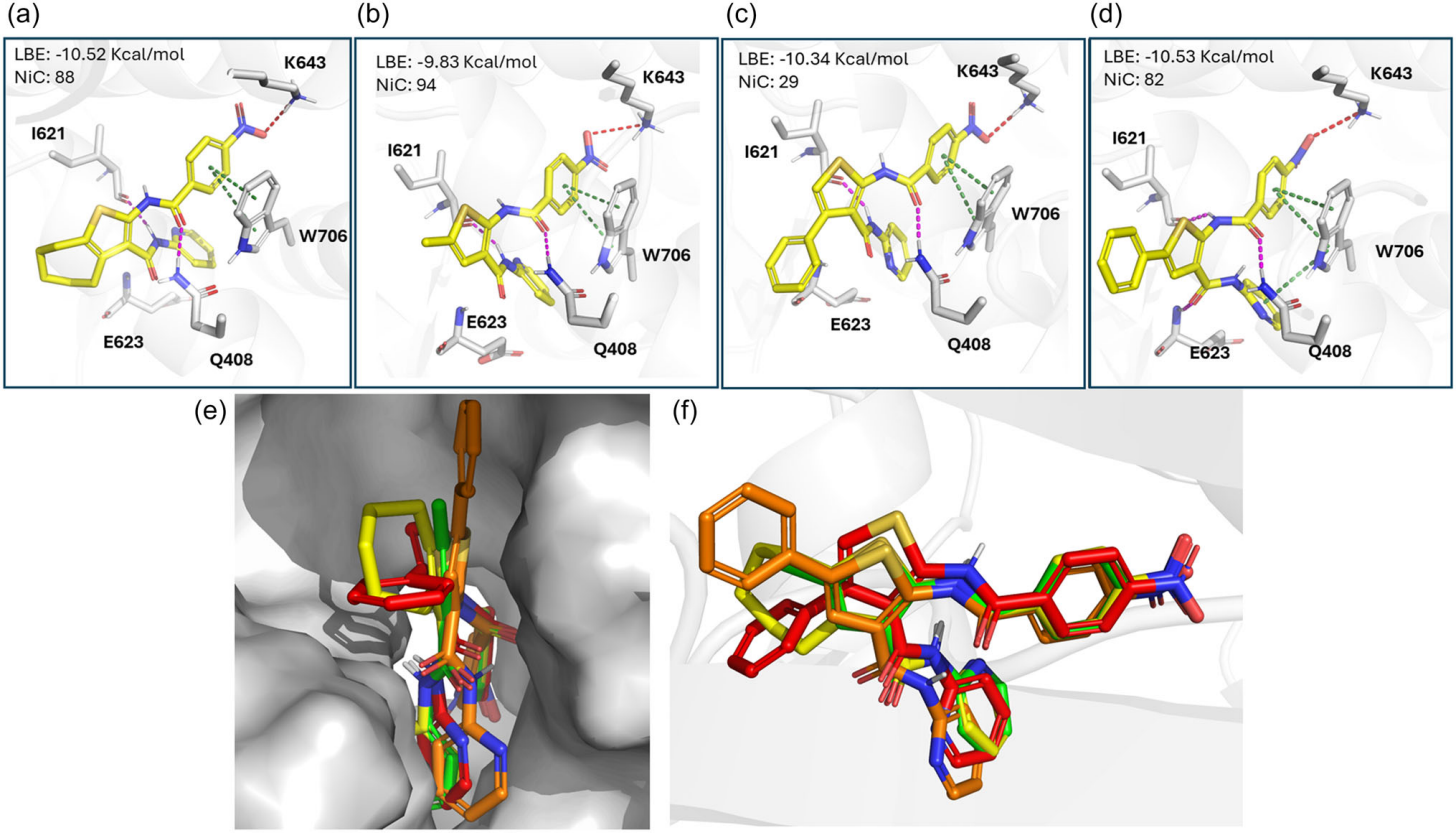
(a–d) 3D of the predicted interaction between compounds **1**, 5, **7**, and **9**, respectively. The type of ligand–protein interactions is color‐coded as follows: hydrogen bond, magenta; π–π interaction, green; salt bridge, red. (e,f) Overlap among the binding modes predicted for compounds **1** (yellow), **5** (green), **7** (red), and **9** (orange).

Consistent with previous docking studies [21], the PA_C_–PB1_N_ complex conformation corresponding to PDB ID 3CM8 [23] was selected for the present investigation. Docking studies performed with AutoDock confirmed that compounds **1**, **5**, **7**, and **9** could interact favorably with the binding site. The four compounds exhibited substantial overlap in their binding modes, indicating that the overall ligand orientation within the pocket is largely conserved (Figure 3a–d). However, this binding mode differs from that previously reported for related analogs. In particular, the *p*‐nitrobenzoyl moiety appears to play a significant role in driving ligand recognition and in establishing the main interactions with the PA_C_. Specifically, the nitro group forms a salt bridge with the positively charged head of K643, and the aromatic ring engages in a double π–π interaction with the side chain of W706. Additionally, the oxygen of the amide linker connecting the *p*‐nitrobenzoyl group to the core forms a hydrogen bond with Q408. An additional hydrogen bond with I621 anchors the ligand within the binding site through interaction with the nitrogen of the C‐3 amide linker for compounds **1**, **5**, and **7** and the C‐2 amide linker for compound **9**. In this binding mode, the thiophene‐based core does not form significant direct interactions with the pocket. Instead, it is oriented toward the solvent‐exposed region of the site. Within this shared binding framework, compound **9** exhibited an expanded interaction network, which included an additional π–π interaction between the pyridine ring and W706, as well as a hydrogen bond between the C‐3 amide linker oxygen and E623.

Notably, all four compounds were associated with comparable ligand binding energy values ranging from −9.83 to −10.53 Kcal/mol, consistent with a similar energetic favourability of the predicted pose once adopted. However, a clear difference emerged when pose reliability was considered. This aspect was assessed through the NiC parameter (number in clusters), which reflects the statistical robustness of a given docking solution. Values close to 100 indicate highly reliable poses, with a value of 100 corresponding to the identification of a single dominant binding mode, whereas lower values indicate that the same pose is less consistently sampled during docking.

In this context, compounds **1**, **5**, and **9** showed NiC values above 80 (i.e., 88, 94, and 83, respectively), while **7** displayed a markedly lower value (i.e., 29). These results suggest that, although the predicted binding mode of **7** is energetically comparable to those obtained for the other analogs, it is statistically less probable.

Notably, comparison of the predicted poses for the entire set considered in this analysis (i.e., **1**, **5**, **7,** and **9**), showed that, in compound **7**, the C‐4 ring is oriented toward the protein surface, with a consequent increased likelihood of steric clashes (Figure 3e), while the thiophene core is not aligned with the other predicted poses (Figure 3f).

In conclusion, docking studies on compounds **1**, **5**, **7**, and **9** suggested a common binding mode, in which the *p*‐nitrobenzoyl moiety appears to play a significant role in driving ligand recognition, forming the main interactions with W706 and K643. The presence of this moiety causes the molecules to adopt a different binding mode from that previously reported [21], whereby the compounds interact with the HSP‐1 and HSP‐2 of PA_C_. Additionally, hydrogen bonds are formed between the C‐2 or C‐3 amide bonds and I621 andQ408, with the latter being identified as important for PA‐PB1 heterodimerization inhibition.

Finally, docking studies have suggested that the presence of bulky groups at the C‐4 position of the thiophene ring, as in compound **7**, can prevent the *p*‐nitrobenzoyl moiety from orienting properly within HSP‐1 or cause clashes with the protein.

## 3 Conclusions

In this study, we investigated the modification of the cycloheptyl moiety linked to the central thiophene scaffold of the cHTC derivative **1**, a region that had never previously undergone modification. The new derivatives were designed to explore the SAR and the contribution of the cycloheptyl moiety to aqueous solubility, a property that has traditionally been inadequate for the cHTC series. In addition, a novel synthetic procedure was developed in this study for the synthesis of 2‐amidothiophene 3‐carboxamide compounds. This novel procedure enables the utilization of the one‐pot Gewald reaction, while introducing the aromatic amide substituent at the C‐3 position exclusively in the final step. This facilitated the synthesis of all the designed analogs in high yields, over brief periods, and with a decrease of synthetic steps. The novel compounds were then subjected to anti‐IV activity testing, yielding intriguing findings that contributed to the comprehensive SAR of this series of analogs. Notably, tests revealed critical information regarding the moiety adjacent to the thiophene, a previously overlooked aspect. Among the newly synthesized compounds, two have demonstrated particularly noteworthy results. Derivative **9**, which features a phenyl at the C‐5 position in place of the cycloheptyl of compound **1**, displayed potent and broad‐spectrum antiviral activity against different IAV and IBV strains (EC_50_ = 0.19–1.11 µM). Conversely, methyl derivative **5** exhibited a modest decrease in anti‐IV activity (EC_50_ of 2 µM), while concurrently demonstrating a substantial increase in aqueous solubility, exceeding 40‐fold the solubility of the starting hit **1**. Furthermore, both derivatives exhibited the same mechanism of action as the cHTC series, namely the inhibition of the PA–PB1 interaction. In addition, docking studies conducted on the PA_C_ cavity also confirmed that compounds **5** and **9** retained an overlapping binding pose with hit compound **1**.

The PA–PB1 interaction represents an innovative and yet‐to‐be‐explored target for the development of anti‐IV agents. The data obtained in this study will be instrumental in the future design of new molecules. Indeed, the information obtained here could be combined with that recently reported on the cHTC class, which has seen the exploration of substituents at the C‐2 and C‐3 positions [29]. The combination of these insights has the potential to maintain potent anti‐IV activity with low‐nanomolar EC_50_s and increased aqueous solubility. The latter has traditionally been a weakness of this class of compounds due to the high lipophilicity required for interaction with PA_C_. Therefore, the potential to obtain PA–PB1 interaction inhibitors endowed with potent anti‐IV activity and a discrete polarity and water solubility represents a significant advancement in this research field.

## 4 Experimental Section

### 4.1 General Chemistry

All reactions were routinely monitored by TLC on silica gel 60F254 (Merck) and visualized by using UV or iodine. Flash column chromatography was performed on Merck silica gel 60 (mesh 230–400). After extraction, organic solutions were dried over anhydrous Na_2_SO_4_, filtered, and concentrated with a Büchi rotary evaporator at reduced pressure. Yields are those of purified products and were not optimized. ^1^H NMR and ^13^C NMR spectra were recorded on a Bruker Avance DRX‐400. Chemical shifts (δ) are reported in ppm relative to TMS and calibrated using residual undeuterated solvent as an internal reference. Coupling constants (*J*) are reported in Hz. Spectra were acquired at 298 K. Data processing was performed with Bruker TopSpin 5.0.0 software, and the spectral data are consistent with the assigned structures. The spin multiplicities are indicated by the symbols: s (singlet), d (doublet), t (triplet), q (quartet), dt (doublet of triplets), m (multiplet), and bs (broad singlet). For the target compounds, the purity (>95%) was revealed at 254 nm and evaluated by HPLC analysis using a Jasco LC‐4000 instrument equipped with a UV–visible Diode Array Jasco MD‐4015 and an XTerra C18 Column (Waters), 5 μm, 4.6 mm × 150 mm or a Gemini C18 (Phenomenex), 3 µm, 100 mm × 2 mm (for compound **13**). The methods have been specified for each compound. The resulting chromatograms were then analyzed using the ChromNAV 2.0 Chromatography Data System software. Peak retention time is given in minutes.

High‐Resolution Mass Spectrometry (HRMS) was based on electrospray ionization (ESI) in positive or negative polarity, as indicated for each compound, using a QTOF Ion Mobility Agilent 6560 equipped with U(H)PLC 1290 Infinity II. Commercially available starting materials, reagents, and solvents were used as supplied. Compound **47** [33] were synthesized as reported in literature.

#### 4.1.1 General Procedure for Gewald Reation (Method A)

In an N_2_ atmosphere, the appropriate ketone or aldehyde (1.0 equiv) was dissolved in dry EtOH (2 mL per mmol). Then, ethyl cyanoacetate (1.5 equiv) and sulfur (2.0 equiv) were added to this solution. The reaction mixture was then cooled to 0°C, and morpholine (3.0 equiv) was added. The reaction was stirred at 50°C for the time indicated for each compound. Afterwards, the reaction was allowed to cool to room temperature (rt) and the excess sulfur was filtered under vacuum. The filtrate (EtOH) was evaporated to dryness, and the resulting crude product was purified by chromatography as described for each compound, to obtain the desired product.

#### 4.1.2 Ethyl 2‐amino‐5,6,7,8‐tetrahydro‐4*H*‐cyclohepta[*b*]thiophene‐3‐carboxylate (17)

The title compound was prepared using cycloheptanone (8.92 mmol, 1.00 g) through Method A after 10 h in 89% yield (1.90 g) as a yellow solid (chromatography column eluted with cyclohexane 80%/EtOAc 20%) [33]. ^1^H NMR (400 MHz, CDCl_3_): δ 1.37 (t, *J* = 7.1 Hz, 3H, OCH_2_ *CH*_3_), 1.60–1.68 (m, 4H, cycloheptyl‐CH_2_ x2), 1.78–1.83 (m, 2H, cycloheptyl‐CH_2_), 2.55–2.61 (m, 2H, cycloheptyl‐CH_2_), 2.98–3.01 (m, 2H, cycloheptyl‐CH_2_), 4.30 (q, *J* = 7.0 Hz, 2H, O*CH*_2_CH_3_), 5.75–5.82 (m, 2H, NH_2_).

#### 4.1.3 General Procedure for C‐2 Amidation (Method B)

In a N_2_ atmosphere, the appropriate intermediate (1.0 equiv) was dissolved in dry pyridine (2 mL per mmol), and 4‐nitrobenzoyl chloride (1.5 equiv) was added. The reaction mixture was stirred at rt for the time indicated for each compound. Then, it was poured into ice/water to obtain a precipitate, which was filtered and purified, or used as such, as described for each compound.

#### 4.1.4 Ethyl 2‐(4‐nitrobenzamido)−5,6,7,8‐tetrahydro‐4*H*‐cyclohepta[*b*]thiophene‐3‐carboxylate (18)

The title compound was prepared starting from **17** (3.34 mmol, 0.80 g) through Method B after 60 min in 97% yield (1.26 g) as a yellow solid. ^1^H NMR (400 MHz, CDCl_3_): δ 1.45 (t, *J* = 7.1 Hz, 3H, OCH_2_ *CH*_3_), 1.63–1.75 (m, 4H, cycloheptyl‐CH_2_ x2), 1.88–1.92 (m, 2H, cycloheptyl‐CH_2_), 2.78–2.83 (m, 2H, cycloheptyl‐CH_2_), 3.07–3.15 (m, 2H, cycloheptyl‐CH_2_), 4.44 (q, *J* = 7.1 Hz, 2H, O*CH*_2_CH_3_), 8.19 (d, *J* = 8.7 Hz, 2H, Ar‐H), 8.40 (d, *J* = 8.7 Hz, 2H, Ar‐H), 12.54 (s, 1H, NH).

#### 4.1.5 General Procedure for the C‐3 Amidation (Method C)

To a solution of the 2‐aminopyridine (3.0 equiv) in dry THF (10 mL per mmol), 1 M LiHMDS in THF (3.1 equiv) was added dropwise at 0°C. The reaction mixture was stirred for 10 min at rt, and then, the appropriate intermediate (1.0 equiv) was added at 0°C. The solution was stirred at rt for the time indicated for each compound. The mixture was then poured into ice/water, and 2N HCl was added until the pH was 8, obtaining a precipitate that was filtered and purified as described for each compound.

#### 4.1.6 2‐(4‐Nitrobenzamido)‐*N*‐(pyridin‐2‐yl)−5,6,7,8‐tetrahydro‐4*H*‐cyclohepta[*b*]thiophene‐3‐carboxamide (1)

The title compound was prepared starting from compound **18** (0.26 mmol, 0.10 g) through Method C after 60 min [28]. After crystallization by EtOH/DMF, the title compound was obtained as a yellow solid in 74% yield (0.08 g). ^1^H NMR (400 MHz, DMSO‐*d*_6_): δ 1.51–1.63 (m, 4H, cycloheptyl‐CH_2_ x2), 1.78–1.89 (m, 2H, cycloheptyl‐CH_2_), 2.70–2.81 (m, 4H, cycloheptyl‐CH_2_ x2), 7.12–7.19 (m, 1H, Ar‐H), 7.75–7.83 (m, 1H, Ar‐H), 8.14 (d, *J* = 7.4 Hz, 2H, Ar‐H), 8.23 (d, *J* = 7.3 Hz, 1H, Ar‐H), 8.34 (d, *J* = 7.3 Hz, 2H, Ar‐H), 8.37–8.42 (m, 1H, Ar‐H), 10.59 (bs, 1H, NH), 11.30 (bs, 1H, NH).

#### 4.1.7 2‐(4‐Nitrobenzamido)−5,6,7,8‐tetrahydro‐4*H*‐cyclohepta[*b*]thiophene‐3‐carboxylic acid (19)

To a solution of compound **18** (2.06 mmol, 0.80 g) in THF (8 mL), a solution of 1 M LiOH in H_2_O (8.24 mL) was added, and the mixture was stirred at rt for 12 h. Then, it was poured into ice/water, 2N HCl was added up to pH 4, and the obtained precipitate was filtered. The title compound was obtained as a yellow solid in 65% yield (0.48 g). ^1^H NMR (400 MHz, DMSO‐*d*_6_): δ 1.47–1.56 (m, 4H, cycloheptyl‐CH_2_ x2), 1.79–1.83 (m, 2H, cycloheptyl‐CH_2_), 2.73–2.77 (m, 2H, cycloheptyl‐CH_2_), 3.09–3.15 (m, 2H, cycloheptyl‐CH_2_), 8.12 (d, *J* = 8.6 Hz, 2H, Ar‐H), 8.44 (d, *J* = 8.6 Hz, 2H, Ar‐H), 12.37 (bs, 1H, NH), 13.55 (bs, 1H, OH).

#### 4.1.8 2‐(4‐Nitrophenyl)−6,7,8,9‐tetrahydro‐4*H*,5*H*‐cyclohepta [4,5]thieno[2,3‐*d* [1,3] oxazin‐4‐one (20)

Under N_2_ atmosphere, to a solution of compound **19** (0.55 mmol, 0.20 g) in dry DMSO, TBTU (1.38 mmol, 0.44 g), DIPEA (1.93 mmol, 0.34 mL), and 2‐aminopyridine (0.61 mmol, 0.06 g) were added, and the mixture was stirred at rt for 60 min. Then, the mixture was poured into ice/water, and the obtained precipitate was filtered. The title compound was obtained as a yellow solid in 88% yield (0.17 g). ^1^H NMR (400 MHz, DMSO‐*d*_6_): δ 1.57–1.68 (m, 4H, cycloheptyl‐CH_2_ x2), 1.85–1.93 (m, 2H, cycloheptyl‐CH_2_), 2.83–2.95 (m, 2H, cycloheptyl‐CH_2_), 3.12–3.18 (m, 2H, cycloheptyl‐CH_2_), 8.29–8.41 (m, 4H, Ar‐H).

#### 4.1.9 Ethyl 2‐amino‐4,5,6,7‐tetrahydro‐1‐benzothiophene‐3‐carboxylate (21)

The title compound was prepared using cyclohexanone (10.19 mmol, 1.05 mL) through Method A (reaction time: 90 min) as a white solid in 90% yield (2.07 g) after purification by chromatography column eluting with cyclohexane/EtOAc (80:20) [34]. ^1^H NMR (400 MHz, CDCl_3_): δ 1.33 (t, *J* = 7.1 Hz, 3H, OCH_2_ *CH*_3_), 1.67–1.81 (m, 4H, cyclohexyl‐CH_2_ x2), 2.42–2.51 (m, 2H, cyclohexyl‐CH_2_), 2.67–2.83 (m, 2H, cyclohexyl‐CH_2_), 4.27 (q, *J* = 7.1 Hz, 2H, O*CH*_2_CH_3_), 5.95 (bs, 2H, NH_2_).

#### 4.1.10 Ethyl 2‐amino‐5,6‐dihydro‐4*H*‐cyclopenta[*b*]thiophene‐3‐carboxylate (22)

The title compound was prepared using cyclopentanone (11.89 mmol, 1.05 mL) through Method A (reaction time: 60 min) as a white solid in 94% yield (2.36 g) after purification by chromatography column eluting with cyclohexane/EtOAc (80:20) [35]. ^1^H NMR (400 MHz, CDCl_3_): δ 1.38 (t, *J* = 7.1 Hz, 3H, OCH_2_ *CH*_3_), 2.27–2.34 (m, 2H, cyclopentyl‐CH_2_), 2.70–2.73 (m, 2H, cyclopentyl‐CH_2_), 2.80–2.84 (m, 2H, cyclopentyl‐CH_2_), 4.25 (q, *J* = 7.1 Hz, 2H, O*CH*_2_CH_3_), 5.91 (bs, 2H, NH_2_).

#### 4.1.11 Ethyl 2‐amino‐4,5‐dimethylthiophene‐3‐carboxylate (23)

The title compound was prepared using butan‐2‐one (6.93 mmol, 0.63 mL) through Method A (reaction time: 4 h) as a yellow solid in 85% yield (1.17 g) after purification by chromatography column eluting with cyclohexane/EtOAc (90:10) [36]. ^1^H NMR (400 MHz, CDCl_3_): δ 1.36 (t, *J* = 7.1 Hz, 3H, OCH_2_ *CH*_3_), 2.17 (s, 3H, CH_3_), 2.19 (s, 3H, CH_3_), 4.30 (q, *J* = 7.1 Hz, 2H, O*CH*_2_CH_3_) and 5.91 (s, 2H, NH_2_).

#### 4.1.12 Ethyl 2‐amino‐5‐methylthiophene‐3‐carboxylate (24)

The title compound was prepared using propionaldehyde (17.2 mmol, 1.25 mL) through Method A (reaction time: 5 h) as a yellow solid in 70% yield (2.23 g) after purification by chromatography column eluting with cyclohexane/EtOAc (90:10) [37]. ^1^H NMR (400 MHz, CDCl_3_): δ 1.30 (t, *J* = 7.1 Hz, 3H, OCH_2_ *CH*_3_), 2.21 (s, 3H, CH_3_), 4.23 (q, *J* = 7.1 Hz, 2H, O*CH*_2_CH_3_), 5.99 (s, 2H, NH_2_), 6.58 (s, 1H, H‐4).

#### 4.1.13 Ethyl 2‐amino‐5‐ethylthiophene‐3‐carboxylate (25)

The title compound was prepared using butyraldehyde (6.93 mmol, 0.63 mL) through Method A (reaction time: 4 h) as a yellow solid in 69% yield (0.95 g) after purification by chromatography column eluting with cyclohexane/EtOAc (85:15) [36]. ^1^H NMR (400 MHz, CDCl_3_): δ 1.25 (t, *J* = 7.0 Hz, 3H, CH_2_ *CH*_3_), 1.35 (t, *J* = 7.1 Hz, 3H, OCH_2_ *CH*_3_), 2.63 (q, *J* = 7.2 Hz, 2H, *CH*_2_CH_3_), 4.28 (q, *J* = 7.1 Hz, 2H, O*CH*_2_CH_3_), 5.79 (s, 2H, NH_2_), 6.66 (s, 1H, H‐4).

#### 4.1.14 Ethyl 2‐amino‐4‐phenylthiophene‐3‐carboxylate (26)

The title compound was prepared using acetophenone (4.29 mmol, 0.50 mL) through Method A (reaction time: 3 h) as a yellow solid in 77% yield (0.82 g) after purification by chromatography column eluting with cyclohexane/EtOAc (95:5) [38]. ^1^H NMR (400 MHz, CDCl_3_): δ 0.96 (t, *J* = 7.0 Hz, 3H, OCH_2_ *CH*_3_), 4.06 (q, *J* = 7.1 Hz, 2H, O*CH*_2_CH_3_), 6.11–6.20 (m, 3H, NH_2_ and H‐5), 7.31–7.57 (m, 5H, Ar‐H).

#### 4.1.15 Ethyl 2‐amino‐5‐methyl‐4‐phenylthiophene‐3‐aarboxylate (27)

The title compound was prepared using propiophenone (3.73 mmol, 0.50 mL) through Method A (reaction time: 24 h) as an orange solid in 74% yield (0.72 g) after purification by chromatography column eluting with cyclohexane/EtOAc (95:5) [39]. ^1^H NMR (400 MHz, CDCl_3_): δ 0.83 (t, *J* = 7.1 Hz, 3H, OCH_2_ *CH*_3_), 2.07 (s, 3H, CH_3_), 3.95 (q, *J* = 7.1 Hz, 2H, O*CH*_2_CH_3_), 5.97 (bs, 2H, NH_2_), 7.18–7.20 (m, 2H, Ar‐H), 7.29–7.38 (m, 3H, Ar‐H).

#### 4.1.16 Ethyl 2‐amino‐5‐phenylthiophene‐3‐carboxylate (28)

The title compound was prepared using 3‐phenylpropionaldehyde (8.32 mmol, 0.97 mL) through Method A (reaction time: 2 h) as a yellow solid in 91% yield (1.87 g) after purification by chromatography column eluting with cyclohexane/EtOAc (80:20) [40]. ^1^H NMR (400 MHz, CDCl_3_): δ 1.39 (t, *J* = 7.1 Hz, 3H, OCH_2_ *CH*_3_), 4.33 (q, *J* = 7.1 Hz, 2H, O*CH*_2_CH_3_), 6.03 (bs, 2H, NH_2_), 7.23 (t, *J* = 7.3 Hz, 1H, Ar‐H), 7.28 (s, 1H, H‐4), 7.35 (t, *J* = 7.0 Hz, 2H, Ar‐H), 7.46 (d, *J* = 7.1 Hz, 2H, Ar‐H).

#### 4.1.17 Ethyl 2‐amino‐5‐benzylthiophene‐3‐carboxylate (29)

The title compound was prepared using 4‐phenyl‐2‐butenal (7.45 mmol, 0.98 mL) through Method A (reaction time: 2 h) as a yellow solid in 65% yield (1.87 g) after purification by chromatography column eluting with cyclohexane/EtOAc (90:10) [41]. ^1^H NMR (400 MHz, CDCl_3_): δ 1.35 (t, *J* = 7.1 Hz, 3H, OCH_2_ *CH*_3_), 3.94 (s, 2H, CH_2_), 4.28 (q, *J* = 7.1 Hz, 2H, O*CH*_2_CH_3_), 5.83 (bs, 2H, NH_2_), 6.73 (s, 1H, H‐4), 7.24–7.30 (m, 3H, Ar‐H), 7.34 (t, *J* = 7.8 Hz, 2H, Ar‐H).

#### 4.1.18 Ethyl 2‐amino‐6‐ethyl‐4,5,6,7‐tetrahydro‐1‐benzothiophene‐3‐carboxylate (30)

The title compound was prepared using 4‐ethylcyclohexan‐1‐one (39.00 mmol, 5.60 mL) through Method A (reaction time: 12 h) as a yellow solid in 81% yield (8.00 g) after purification by chromatography column eluting with cyclohexane/EtOAc (90:10) [35]. ^1^H NMR (400 MHz, DMSO‐_d6_): δ 0.86 (t, *J* = 7.4 Hz, 3H, CH_2_ *CH*_3_), 1.19 (t, *J* = 7.0 Hz, 3H, OCH_2_ *CH*_3_), 1.22–1.34 (m, 3H, *CH*_2_CH_3_ and cyclohexyl‐CH), 1.48–1.53 (m, 1H, cyclohexyl‐CH_2_ x½), 1.75–1.81 (m, 1H, cyclohexyl‐CH_2_ x½), 1.98–2.03 (m, 1H, cyclohexyl‐CH_2_ x½), 2.38–2.41 (m, 1H, cyclohexyl‐CH_2_ x½), 2.52–2.57 (m, 1H, cyclohexyl‐CH_2_ x½), 2.63–2.67 (m, 1H, cyclohexyl‐CH_2_ x½), 4.10 (q, *J* = 7.0 Hz, 2H, O*CH*_2_CH_3_), 7.13 (bs, 2H, NH_2_).

#### 4.1.19 Ethyl 2‐amino‐6‐ethyl‐4,5,6,7‐tetrahydrothieno[2,3‐*c*]pyridine‐3‐carboxylate (31)

The title compound was prepared using 1‐ethylpiperidin‐4‐one (39.00 mmol, 5.29 mL) through Method A (reaction time: 12 h) as a yellow solid in 95% yield (9.42 g) after purification by chromatography column eluting with cyclohexane/EtOAc (70:30) [42]. ^1^H NMR (400 MHz, DMSO‐_d6_): δ 0.99 (t, *J* = 7.2 Hz, 3H, CH_2_ *CH*_3_), 1.20 (t, *J* = 7.2 Hz, 3H, OCH_2_ *CH*_3_), 2.41 (q, *J* = 7.1 Hz, 2H, *CH*_2_CH_3_), 2.54 (t, *J* = 5.6 Hz, 2H, piperidinyl‐CH_2_), 2.59–2.65 (m, 2H, piperidinyl‐NCH_2_), 3.21–3.27 (m, 2H, piperidinyl‐NCH_2_), 4.11 (q, *J* = 7.1 Hz, 2H, O*CH*_2_CH_3_), 7.17 (bs, 2H, NH_2_).

#### 4.1.20 6‐*tert*‐Butyl 3‐ethyl 2‐amino‐4,7‐dihydrothieno[2,3‐*c*]pyridine‐3,6(5*H*)‐dicarboxylate (32)

The title compound was prepared using *te* *rt*‐butyl 4‐oxo‐1‐piperidinecarboxylate (2.51 mmol, 0.50 g) through Method A (reaction time: 3 h) as a yellow solid in 55% yield (0.45 g) after purification by chromatography column eluting with cyclohexane/EtOAc (80:20) [43]. ^1^H NMR (400 MHz, CDCl_3_): δ 1.36 (t, *J* = 7.1 Hz, 3H, OCH_2_ *CH*_3_), 1.50 (s, 9H, C(CH_3_)_3_), 2.81–2.86 (m, 2H, piperidinyl‐CH_2_), 3.86 (t, *J* = 5.7 Hz, 2H, piperidinyl‐NCH_2_), 4.28 (q, *J* = 7.1 Hz, 2H, O*CH*_2_CH_3_), 4.33–4.39 (m, 2H, piperidinyl‐NCH_2_), 6.02 (bs, 2H, NH_2_).

#### 4.1.21 Ethyl 2‐[(4‐nitrobenzoyl)amino]‐4,5,6,7‐tetrahydro‐1‐benzothiophene‐3‐carboxylate (33)

The title compound was prepared starting from **21** (4.44 mmol, 1.00 g) through Method B (reaction time: 1 h) as a yellow solid in 84% yield (1.40 g). ^1^H NMR (400 MHz, DMSO‐_d6_): δ 1.45 (t, *J* = 7.1 Hz, 3H, OCH_2_ *CH*_3_), 1.72–1.78 (m, 4H, cyclohexyl‐CH_2_ x2), 2.63–2.71 (m, 2H, cyclohexyl‐CH_2_), 2.73–2.77 (m, 2H, cyclohexyl‐CH_2_), 4.35 (q, *J* = 7.1 Hz, 2H, O*CH*_2_CH_3_), 8.15 (d, *J* = 8.8 Hz, 2H, Ar‐H), 8.44 (d, *J* = 8.8 Hz, 2H, Ar‐H), 12.11 (bs, 1H, NH).

#### 4.1.22 Ethyl 2‐[(4‐nitrobenzoyl)amino]‐5,6‐dihydro‐4*H*‐cyclopenta[*b*]thiophene‐3‐carboxylate (34)

The title compound was prepared starting from **22** (4.50 mmol, 0.95 g) through Method B (reaction time: 1 h) as a yellow solid in 100% yield (1.60 g). ^1^H NMR (400 MHz, CDCl_3_): δ 1.41 (t, *J* = 7.12 Hz, 3H, OCH_2_ *CH*_3_), 2.41–2.44 (m, 2H, cyclopentyl‐CH_2_), 2.89–2.96 (m, 2H, cyclopentyl‐CH_2_ x2), 4.37 (q, *J* = 7.12 Hz, 2H, O*CH*_2_CH_3_), 8.19 (d, *J* = 8.7 Hz, 2H, Ar‐H), 8.38 (d, *J* = 8.7 Hz, 2H, Ar‐H), 12.23 (bs, 1H, NH).

#### 4.1.23 Ethyl 4,5‐dimethyl‐2‐[(4‐nitrobenzoyl)amino]thiophene‐3‐carboxylate (35)

The title compound was prepared starting from **23** (2.51 mmol, 0.50 g) through Method B (reaction time: 1 h) as a yellow solid in 97% yield (0.85 g) after trituration by Et_2_O. ^1^H NMR (400 MHz, DMSO‐*d*_6_): δ 1.36 (t, *J* = 7.1 Hz, 3H, OCH_2_ *CH*_3_), 2.24 (s, 3H, CH_3_), 2.29 (s, 3H, CH_3_), 4.36 (q, *J* = 7.1 Hz, 2H, O*CH*_2_CH_3_), 8.14 (d, *J* = 8.7 Hz, 2H, Ar‐H), 8.44 (d, *J* = 8.7 Hz, 2H, Ar‐H), 12.06 (bs, 1H, NH).

#### 4.1.24 Ethyl 5‐methyl‐2‐[(4‐nitrobenzoyl)amino]thiophene‐3‐carboxylate (36)

The title compound was prepared starting from **24** (3.77 mmol, 0.70 g) through Method B (reaction time: 30 min) as a yellow solid in 87% yield (1.10 g). ^1^H NMR (400 MHz, CDCl_3_): δ 1.43 (t, *J* = 7.1 Hz, 3H, OCH_2_ *CH*_3_), 2.46 (s, 3H, CH_3_), 4.48 (q, *J* = 7.1 Hz, 2H, O*CH*_2_CH_3_), 6.95–6.97 (m, 1H, H‐4), 8.21 (d, *J* = 8.7 Hz, 2H, Ar‐H), 8.40 (d, *J* = 8.7 Hz, 2H, Ar‐H), 12.16 (bs, 1H, NH).

#### 4.1.25 Ethyl 5‐ethyl‐2‐[(4‐nitrobenzoyl)amino]thiophene‐3‐carboxylate (37)

The title compound was prepared starting from **25** (2.76 mmol, 0.55 g) through Method B (reaction time: 1 h) as a yellow solid in 90% yield (0.87 g). ^1^H NMR (400 MHz, CDCl_3_): δ 1.36 (t, *J* = 7.4 Hz, 3H, CH_2_ *CH*_3_), 1.44 (t, *J* = 7.1 Hz, 3H, OCH_2_ *CH*_3_), 2.82 (q, *J* = 7.4 Hz, 2H, *CH*_2_CH_3_), 4.41 (q, *J* = 7.1 Hz, 2H, O*CH*_2_CH_3_), 6.98 (s, 1H, H‐4), 8.21 (d, *J* = 8.6 Hz, 2H, Ar‐H), 8.41 (d, *J* = 8.6 Hz, 2H, Ar‐H), 12.17 (bs, 1H, NH).

#### 4.1.26 Ethyl 2‐[(4‐nitrobenzoyl)amino]‐4‐phenylthiophene‐3‐carboxylate (38)

The title compound was prepared starting from **26** (1.26 mmol, 0.50 g) through Method B (reaction time: 2 h) as a yellow solid in 97% yield (0.77 g). ^1^H NMR (400 MHz, CDCl_3_): δ 0.98 (t, *J* = 7.1 Hz, 3H, OCH_2_ *CH*_3_), 2.22 (s, 3H, CH_3_), 4.05 (q, *J* = 7.1 Hz, 2H, O*CH*_2_CH_3_), 6.75 (s, 1H, H‐5), 7.31–7.38 (m, 5H, Ar‐H), 8.24 (d, *J* = 8.7 Hz, 2H, Ar‐H), 8.42 (d, *J* = 8.7 Hz, 2H, Ar‐H), 12.61 (bs, 1H, NH).

#### 4.1.27 Ethyl 5‐methyl‐2‐[(4‐nitrobenzoyl)amino]‐4‐phenylthiophene‐3‐carboxylate (39)

The title compound was prepared starting from **27** (0.77 mmol, 0.20 g) through Method B (reaction time: 2 h) as a yellow solid in 80% yield (0.25 g). ^1^H NMR (400 MHz, CDCl_3_): δ 0.85 (t, *J* = 7.1 Hz, 3H, OCH_2_ *CH*_3_), 2.22 (s, 3H, CH_3_), 4.05 (q, *J* = 7.1 Hz, 2H, O*CH*_2_CH_3_), 7.19–7.21 (m, 2H, Ar‐H), 7.33–7.46 (m, 3H, Ar‐H), 8.07 (d, *J* = 8.6 Hz, 2H, Ar‐H), 8.43 (d, *J* = 8.6 Hz, 2H, Ar‐H), 12.55 (bs, 1H, NH).

#### 4.1.28 Ethyl 2‐[(4‐nitrobenzoyl)amino]‐5‐phenylthiophene‐3‐carboxylate (40)

The title compound was prepared starting from **28** (4.04 mmol, 1.00 g) through Method B (reaction time: 4 h) as a yellow solid in 70% yield (1.12 g) after purification by flash chromatography eluting with cyclohexane/EtOAc (85:15). ^1^H NMR (400 MHz, DMSO‐*d*_6_): δ 1.39 (t, *J* = 7.1 Hz, 3H, OCH_2_ *CH*_3_), 4.44 (q, *J* = 7.1 Hz, 2H, O*CH*_2_CH_3_), 7.32–7.36 (m, 1H, Ar‐H), 7.43–7.46 (m, 2H, Ar‐H), 7.63 (s, 1H, H‐4), 7.69–7.71 (m, 2H, Ar‐H), 8.20 (d, *J* = 8.7 Hz, 2H, Ar‐H), 8.47 (d, *J* = 8.7 Hz, 2H, Ar‐H), 11.89 (bs, 1H, NH).

#### 4.1.29 Ethyl 5‐benzyl‐2‐[(4‐nitrobenzoyl)amino]thiophene‐3‐carboxylate (41)

The title compound was prepared starting from **29** (1.72 mmol, 0.45 g) through Method B (reaction time: 2 h) as a yellow solid in 81% yield (0.57 g). ^1^H NMR (400 MHz, CDCl_3_): δ 1.43 (t, *J* = 7.1 Hz, 3H, OCH_2_ *CH*_3_), 4.11 (s, 2H, CH_2_), 4.40 (q, *J* = 7.1 Hz, 2H, O*CH*_2_CH_3_), 7.01–7.03 (m, 1H, H‐4), 7.27–7.37 (m, 5H, Ar‐H), 8.18 (d, *J* = 8.7 Hz, 2H, Ar‐H), 8.40 (d, *J* = 8.7 Hz, 2H, Ar‐H), 12.18 (bs, 1H, NH).

#### 4.1.30 Ethyl 6‐ethyl‐2‐[(4‐nitrobenzoyl)amino]−4,5,6,7‐tetrahydro‐1‐benzothiophene‐3‐carboxylate (42)

The title compound was prepared starting from **30** (1.97 mmol, 0.50 g) through Method B (reaction time: 30 min) as a yellow solid in 86% yield (0.68 g). ^1^H NMR (400 MHz, DMSO‐*d*_6_): δ 0.94 (t, *J* = 7.1 Hz, 3H, CH_2_ *CH*_3_), 1.32–1.36 (m, 6H, *CH*_2_CH_3_, OCH_2_ *CH*_3_ and cyclohexyl‐CH), 1.59–1.61 (m, 1H, cyclohexyl‐CH_2_ x^1^/_2_), 1.88–1.92 (m, 1H, cyclohexyl‐CH_2_ x^1^/_2_), 2.24–2.27 (m, 1H, cyclohexyl‐CH_2_ x^1^/_2_), 2.51–2.54 (m, 1H, cyclohexyl‐CH_2_ x^1^/_2_), 2.76–2.80 (m, 1H, cyclohexyl‐CH_2_ x^1^/_2_), 2.89–2.93 (m, 1H, cyclohexyl‐CH_2_ x^1^/_2_), 4.33 (q, *J* = 7.0 Hz, 2H, O*CH*_2_CH_3_), 8.13 (d, *J* = 7.7 Hz, 2H, Ar‐H), 8.44 (d, *J* = 7.7 Hz, 2H, Ar‐H), 12.11 (bs, 1H, NH).

#### 4.1.31 Ethyl 6‐ethyl‐2‐[(4‐nitrobenzoyl)amino]‐4,5,6,7‐tetrahydrothieno[2,3‐*c*]pyridine‐3‐carboxylate (43)

The title compound was prepared starting from **31** (7.86 mmol, 2.00 g) through Method B (reaction time: 1 h) as a yellow solid in 60% yield (1.60 g) after purification by flash chromatography eluting with CHCl_3_/MeOH (95:5). ^1^H NMR (400 MHz, DMSO‐*d*_6_): δ 1.08 (t, *J* = 7.2 Hz, 3H, CH_2_ *CH*_3_), 1.35 (t, *J* = 7.1 Hz, 3H, OCH_2_ *CH*_3_), 2.51 (q, *J* = 7.2 Hz, 2H, *CH*_2_CH_3_), 2.66–2.69 (m, 2H, piperidinyl‐CH_2_), 2.79–2.81 (m, 2H, piperidinyl‐NCH_2_), 3.50–3.53 (m, 2H, piperidinyl‐NCH_2_), 2.51 (q, *J* = 7.1 Hz, 2H, O*CH*_2_CH_3_), 8.14 (d, *J* = 8.9 Hz, 2H, Ar‐H), 8.44 (d, *J* = 8.9 Hz, 2H, Ar‐H), 12.05 (bs, 1H, NH).

#### 4.1.32 6‐*tert*‐Butyl 3‐ethyl 2‐[(4‐nitrobenzoyl)amino]‐4,7‐dihydrothieno[2,3‐*c*]pyridine‐3,6(5*H*)‐dicarboxylate (44)

The title compound was prepared starting from **32** (0.92 mmol, 0.30 g) through Method B (reaction time: 2 h) as a yellow solid in 69% yield (0.30 g) after crystallization by EtOH/DMF. ^1^H NMR (400 MHz, CDCl_3_): δ 1.70 (t, *J* = 7.1 Hz, 3H, OCH_2_ *CH*_3_), 1.52 (s, 9H, C(CH_3_)_3_), 2.93–2.95 (m, 2H, piperidinyl‐CH_2_), 3.69–3.71 (m, 2H, piperidinyl‐NCH_2_), 4.43 (q, *J* = 7.1 Hz, 2H, O*CH*_2_CH_3_), 4.57–4.59 (m, 2H, piperidinyl‐NCH_2_), 8.21 (d, *J* = 8.8 Hz, 2H, Ar‐H), 8.40 (d, *J* = 8.8 Hz, 2H, Ar‐H), 12.50 (bs, 1H, NH).

#### 4.1.33 Ethyl 2‐[(4‐nitrobenzoyl)amino]‐1‐benzothiophene‐3‐carboxylate (45)

To a solution of compound **33** (0.53 mmol, 0.20 g) in benzene (7 mL), the 2,3‐dichloro‐5,6‐dicyano‐1,4‐benzoquinone (1.33 mmol, 0.37 g) was added, and the mixture was stirred at reflux for 10 h. The reaction was poured into a saturated aqueous solution of NaHCO_3_, obtaining a precipitate that was filtered. After purification by flash chromatography eluting with CH_2_Cl_2_, the title compound was obtained in 60% yield (0.11 g) as a brown solid. ^1^H NMR (400 MHz, CDCl_3_): δ 1.58 (t, *J* = 7.1 Hz, 3H, OCH_2_ *CH*_3_), 4.57 (q, *J* = 7.1 Hz, 2H, O*CH*_2_CH_3_), 7.39 (dt, *J* = 1.2 and 8.1 Hz, 1H, Ar‐H), 7.48 (dt, *J* = 1.2 and 8.1 Hz, 1H, Ar‐H), 7.84 (d, *J* = 8.0 Hz, 1H, Ar‐H), 8.28 (d, *J* = 8.6 Hz, 2H, Ar‐H), 8.35 (m, 1H, Ar‐H), 8.42 (d, *J* = 8.6 Hz, 2H, Ar‐H), 13.04 (bs, 1H, NH).

#### 4.1.34 2‐[(4‐Nitrobenzoyl)amino]‐*N*‐pyridin‐2‐yl‐4,5,6,7‐tetrahydro‐1‐benzothiophene‐3‐carboxamide (2)

The title compound was prepared starting from compound **33** (0.53 mmol, 0.20 g) through Method C (reaction time: 2 h) and obtained as a yellow solid in 75% yield (0.17 g) after crystallization by EtOH/DMF. ^1^H NMR (400 MHz, DMSO‐*d*_6_): δ 1.73–1.85 (m, 4H, cyclohexyl‐CH_2_ x2), 2.67–2.74 (m, 2H, cyclohexyl‐CH_2_), 2.76–2.81 (m, 2H, cyclohexyl‐CH_2_), 7.17 (t, *J* = 6.7 Hz, 1H, Ar‐H), 7.85 (t, *J* = 7.6 Hz, 1H, Ar‐H), 8.14–8.22 (m, 3H, Ar‐H), 8.37–8.39 (m, 3H, Ar‐H), 10.04 (bs, 1H, NH), 11.43 (bs, 1H, NH). ^13^C NMR (101 MHz, DMSO‐*d*_6_): δ 22.81, 23.12, 24.46, 25.47, 115.04, 120.36, 121.24, 124.43, 128.69, 129.73, 130.84, 138.67, 138.93, 140.44, 148.60, 150.08, 152.32, 162.58, 164.12. HRMS (ESI) m/z [M–H]^−^ calcd for C_21_H_18_N_4_O_4_S 421.0976, found 421.09705. HPLC: flow, 1 mL/min; isocratic H_2_O 20%/CH_3_CN 80% for 10 min; ret. time: 3.5667 min.

#### 4.1.35 2‐[(4‐Nitrobenzoyl)amino]‐*N*‐pyridin‐2‐yl‐5,6‐dihydro‐4*H*‐cyclopenta[*b*]thiophene‐3‐carboxamide (3)

The title compound was prepared starting from compound **34** (0.83 mmol, 0.30 g) through Method C (reaction time: 2 h) and obtained as a yellow solid in 71% yield (0.24 g) after purification by crystallization with EtOH/DMF. ^1^H NMR (400 MHz, DMSO‐*d*_6_): δ 2.42–2.50 (m, 2H, cyclopentyl‐CH_2_), 2.88 (t, *J* = 6.8 Hz, 2H, cyclopentyl‐CH_2_), 3.08 (t, *J* = 6.8 Hz, 2H, cyclopentyl‐CH_2_), 7.17–7.23 (m, 1H, Ar‐H), 7.88 (dt, *J* = 1.9 and 7.4 Hz, 1H, Ar‐H), 8.15–8.22 (m, 3H, Ar‐H), 8.36–8.41 (m, 1H, Ar‐H), 8.43 (d, *J* = 8.9 Hz, 2H, Ar‐H), 9.07 (bs, 1H, NH), 12.48 (bs, 1H, NH). ^13^C NMR (101 MHz, DMSO‐*d*_6_): δ 28.36, 28.98, 29.85, 113.81, 114.84, 120.76, 124.76, 129.34, 134.21, 138.37, 138.92, 139.72, 148.79, 149.02, 150.35, 151.58, 161.87, 164.38. HRMS (ESI) m/z [M–H]^‐^ calcd for C_20_H_16_N_4_O_4_S 407.0819, found 407.08121. HPLC: flow, 1 mL/min; isocratic H_2_O 20%/CH_3_CN 80% for 15 min; ret. time: 4.2333 min.

#### 4.1.36 4,5‐Dimethyl‐2‐[(4‐nitrobenzoyl)amino]‐*N*‐pyridin‐2‐ylthiophene‐3‐carboxamide (4)

The title compound was prepared starting from **35** (0.60 mmol, 0.20 g) through Method C (reaction time: 12 h) and obtained as a yellow solid in 87% yield (0.21 g) after crystallization by EtOH/DMF. ^1^H NMR (400 MHz, DMSO‐*d*_6_): δ 2.23 (s, 3H, CH_3_), 2.31 (s, 3H, CH_3_), 7.17 (t, *J* = 6.5 Hz, 1H, Ar‐H), 7.85 (t, *J* = 7.8 Hz, 1H, Ar‐H), 8.16 (d, *J* = 8.4 Hz, 2H, Ar‐H), 8.22 (d, *J* = 8.5 Hz, 1H, Ar‐H), 8.33–8.44 (m, 3H, Ar‐H), 10.33 (bs, 1H, NH), 11.54 (bs, 1H, NH). ^13^C NMR (101 MHz, DMSO‐*d*_6_): δ 12.84, 13.42, 115.16, 120.28, 123.84, 124.30, 125.78, 129.05, 129.85, 138.13, 138.57, 139.17, 148.57, 150.02, 152.55, 162.84, 164.28. HRMS (ESI) m/z [M+H]^+^ calcd for C_19_H_16_N_4_O_4_S 397.0965, found 397.09733. HPLC: flow, 1 mL/min; isocratic H_2_O 20%/CH_3_CN 80% for 12 min; ret. time: 2.5233 min.

#### 4.1.37 5‐Methyl‐2‐[(4‐nitrobenzoyl)amino]‐*N*‐pyridin‐2‐ylthiophene‐3‐carboxamide (5)

The title compound was prepared starting from **36** (2.84 mmol, 0.95 g) through Method C (reaction time: 2 h) and obtained as a white solid in 76% yield after purification by flash chromatography eluting with CHCl_3_ (100). ^1^H NMR (400 MHz, CDCl_3_): δ 2.50 (s, 3H, CH_3_), 6.90 (s, 1H, H‐4), 7.13 (t, *J* = 5.8 Hz, 1H, Ar‐H), 7.81 (t, *J* = 8.0 Hz, 1H, Ar‐H), 8.24 (d, *J* = 8.4 Hz, 2H, Ar‐H), 8.34–8.36 (m, 3H, Ar‐H and NH), 8.42 (d, *J* = 8.4 Hz, 2H, Ar‐H), 13.01 (s, 1H, NH). ^13^C NMR (101 MHz, DMSO‐_*d6*_): δ 15.02, 114.44, 115.15, 118.23, 120.35, 124.25, 128.83, 131.82, 137.88, 138.61, 146.65, 148.26, 150.19, 150.97, 161.40, 163.88. HRMS (ESI) m/z [M–H]^−^ calcd for C_18_H_14_N_4_O_4_S 381.0663, found 381.06666. HPLC: flow, 1 mL/min; isocratic H_2_O 25%/CH_3_CN 75% for 10 min; ret. time: 2.9600 min.

#### 4.1.38 5‐Ethyl‐2‐[(4‐nitrobenzoyl)amino]‐*N*‐pyridin‐2‐ylthiophene‐3‐carboxamide (6)

The title compound was prepared starting from **37** (1.43 mmol, 0.50 g) through Method C (reaction time: 2 h) and obtained in 72% yield (0.41 g) as a yellow solid after purification by flash chromatography eluting with CHCl_3_/MeOH (98:2). ^1^H NMR (400 MHz, CDCl_3_): δ 1.37 (t, *J* = 7.5 Hz, 3H, CH_2_ *CH*_3_), 2.85 (q, *J* = 7.5 Hz, 2H, *CH*_2_CH_3_), 6.94 (s, 1H, H‐4), 7.11–7.16 (m, 1H, Ar‐H), 7.81 (dt, *J* = 1.1 and 7.2 Hz, 1H, Ar‐H), 8.24 (d, *J* = 8.8 Hz, 2H, Ar‐H), 8.33–8.38 (m, 2H, Ar‐H), 8.41 (d, *J* = 8.7 Hz, 2H, Ar‐H), 8.46 (bs, 1H, NH), 13.02 (bs, 1H, NH). ^13^C NMR (101 MHz, CDCl_3_): δ 15.51, 23.08, 114.50, 115.03, 116.49, 120.31, 124.23, 128.82, 137.94, 138.64, 139.17, 146.45, 148.19, 150.21, 151.05, 161.41, 146.02. HRMS (ESI) m/z [M+H]^+^ calcd for C_19_H_16_N_4_O_4_S 397.0965, found 397.09712. HPLC: flow, 1 mL/min; isocratic H_2_O 25%/CH_3_CN 75% for 12 min; ret. time: 3.6567 min.

#### 4.1.39 2‐[(4‐Nitrobenzoyl)amino]‐4‐phenyl‐*N*‐pyridin‐2‐ylthiophene‐3‐carboxamide (7)

The title compound was prepared starting from **38** (1.01 mmol, 0.40 g) through Method C (reaction time: 10 h) and obtained as an orange solid in 94% yield (0.42 g) after crystallization by EtOH/DMF. ^1^H NMR (400 MHz, DMSO‐*d*_6_): δ 7.09–7.16 (m, 1H, Ar‐H), 7.20 (s, 1H, H‐5), 7.34–7.53 (m, 5H, Ar‐H), 7.81 (t, *J* = 7.6 Hz, 1H, Ar‐H), 8.11–8.32 (m, 4H, Ar‐H), 8.42 (d, *J* = 8.3 Hz, 2H, Ar‐H), 9.69 (bs, 1H, NH), 12.08 (bs, 1H, NH). ^13^C NMR (101 MHz, DMSO‐*d*_6_): δ 114.87, 117.49, 120.33, 120.44, 124.48, 128.35, 129.02, 129.17, 129.85, 136.11, 138.59, 138.64, 138.82, 143.81, 148.58, 150.18, 151.94, 163.16, 163.97. HRMS (ESI) m/z [M+H]^+^ calcd for C_23_H_16_N_4_O_4_S 445.0965, found 445.09664. HPLC: flow, 1 mL/min; isocratic H_2_O 25%/CH_3_CN 75% for 12 min; ret. time: 4.5400 min.

#### 4.1.40 5‐Methyl‐2‐[(4‐nitrobenzoyl)amino]‐4‐phenyl‐*N*‐pyridin‐2‐ylthiophene‐3‐carboxamide (8)

The title compound was prepared starting from **39** (0.49 mmol, 0.20 g) through Method C (reaction time: 14 h) and obtained as a white solid in 90% yield (0.20 g) after crystallization by EtOH/DMF. ^1^H NMR (400 MHz, DMSO‐*d*_6_): δ 2.21 (s, 3H, CH_3_), 7.09 (t, *J* = 7.2 Hz, 1H, Ar‐H), 7.40 (d, *J* = 7.0 Hz, 2H, Ar‐H), 7.44–7.56 (m, 3H, Ar‐H), 7.78 (t, *J* = 7.1 Hz, 1H, Ar‐H), 8.10 (d, *J* = 8.3 Hz, 1H, Ar‐H), 8.16–8.24 (m, 3H, Ar‐H), 8.43 (d, *J* = 8.7 Hz, 2H, Ar‐H), 8.86 (bs, 1H, NH), 12.44 (bs, 1H, NH). ^13^C NMR (101 MHz, DMSO‐*d*_6_): δ 13.35, 114.58, 119.24, 120.50, 124.66, 127.65, 128.83, 129.57, 129.63, 130.41, 134.05, 135.11, 138.59, 138.68, 142.16, 148.65, 150.24, 151.44, 162.61, 163.89. HRMS (ESI) m/z [M+H]^+^ calcd for C_24_H_18_N_4_O_4_S 459.1122, found 459.11245. HPLC: flow, 1 mL/min; isocratic H_2_O 25%/CH_3_CN 75% for 10 min; ret. time: 6.1333 min.

#### 4.1.41 2‐[(4‐Nitrobenzoyl)amino]‐5‐phenyl‐*N*‐pyridin‐2‐ylthiophene‐3‐carboxamide (9)

The title compound was prepared starting from **40** (0.76 mmol, 0.30 g) through Method C (reaction time: 2h) and obtained as a yellow solid in 95% yield (0.32 g) after crystallization by EtOH/DMF. ^1^H NMR (400 MHz, DMSO‐*d*_6_): δ 7.23 (t, *J* = 5.6 Hz, 1H, Ar‐H), 7.34 (t, *J* = 7.1 Hz, 1H, Ar‐H), 7.46 (t, *J* = 7.4 Hz, 2H, Ar‐H), 7.68 (d, *J* = 7.7 Hz, 2H, Ar‐H), 7.88 (t, *J* = 7.4 Hz, 1H, Ar‐H), 8.20 (t, *J* = 8.8 Hz, 3H, Ar‐H), 8.42–8.55 (m, 4H, Ar‐H), 10.75 (bs, 1H, NH), 13.05 (bs, 1H, NH). ^13^C NMR (101 MHz, DMSO‐*d*_6_): δ 116.01, 117.53, 120.30, 120.78, 124.87, 125.36, 128.09, 129.26, 129.71, 133.34, 133.78, 137.77, 138.68, 146.71, 148.55, 150.30, 151.89, 161.66, 164.67. HRMS (ESI) m/z [M+H]^+^ calcd for C_23_H_16_N_4_O_4_S 445.0965, found 445.09702. HPLC: flow, 1 mL/min; isocratic H_2_O 25%/CH_3_CN 75% for 10 min; ret. time: 4.8767 min.

#### 4.1.42 5‐Benzyl‐2‐[(4‐nitrobenzoyl)amino]‐*N*‐pyridin‐2‐ylthiophene‐3‐carboxamide (10)

The title compound was prepared starting from **41** (1.24 mmol, 0.51 g) through Method C (reaction time: 11 h) and obtained as a yellow solid in 69% yield (0.39 g) after purification by flash chromatography eluting with CH_2_Cl_2_ (100). ^1^H NMR (400 MHz, CDCl_3_): δ 4.13 (s, 2H, CH_2_), 6.89 (s, 1H, H‐4), 7.13 (t, *J* = 5.8 Hz, 1H, Ar‐H), 7.30 (d, *J* = 6.9 Hz, 3H, Ar‐H), 7.32–7.40 (m, 2H, Ar‐H), 7.80 (t, *J* = 8.3 Hz, 1H, Ar‐H), 8.23 (d, *J* = 8.9 Hz, 2H, Ar‐H), 8.28–8.36 (m, 3H, Ar‐H and NH), 8.41 (d, *J* = 8.8 Hz, 2H, Ar‐H), 13.04 (bs, 1H, NH). ^13^C NMR (101 MHz, CDCl_3_): δ 35.94, 114.53, 115.06, 118.13, 120.40, 124.27, 127.10, 128.78, 128.83, 128.99, 136.35, 137.79, 138.64, 139.07, 147.29, 148.23, 150.22, 150.90, 161.47, 163.92. HRMS (ESI) m/z [M–H]^−^ calcd for C_24_H_18_N_4_O_4_S 457.0976, found 457.0975. HPLC: flow, 1 mL/min; isocratic H_2_O 25%/CH_3_CN 75% for 10 min; ret. time: 3.8033 min.

#### 4.1.43 6‐Ethyl‐2‐[(4‐nitrobenzoyl)amino]‐*N*‐pyridin‐2‐yl‐4,5,6,7‐tetrahydro‐1‐benzothiophene‐3‐carboxamide (11)

The title compound was prepared starting from **42** (0.50 mmol, 0.20 g) through Method C (reaction time: 30 min) and obtained as a yellow solid in 63% yield (0.14 g) after purification by flash chromatography eluting with CHCl_3_/MeOH (95:5). ^1^H NMR (400 MHz, DMSO‐*d*_6_): δ 0.96 (t, *J* = 7.4 Hz, 3H, CH_2_ *CH*_3_), 1.33–1.48 (m, 3H, *CH*_2_CH_3_ and cyclohexyl‐CH), 1.63–1.75 (m, 1H, cyclohexyl‐CH_2_ x^1^/_2_), 1.89–1.98 (m, 1H, cyclohexyl‐CH_2_ x^1^/_2_), 2.26–2.38 (m, 1H, cyclohexyl‐CH_2_ x^1^/_2_), 2.73–2.90 (m, 3H, cyclohexyl‐CH_2_ and ‐CH_2_ x^1^/_2_), 7.17 (t, *J* = 5.9 Hz, 1H, Ar‐H), 7.84 (t, *J* = 8.2 Hz, 1H, Ar‐H), 8.11–8.22 (m, 3H, Ar‐H), 8.38 (d, *J* = 7.3 Hz, 3H, Ar‐H), 9.99 (bs, 1H, NH), 11.80 (bs, 1H, NH). ^13^C NMR (101 MHz, DMSO‐*d*_6_): δ 11.94, 25.17, 28.57, 28.74, 30.40, 36.02, 115.04, 120.37, 120.83, 124.45, 128.42, 129.69, 130.71, 138.67, 138.90, 140.78, 148.62, 150.08, 152.29, 162.52, 164.20. HRMS (ESI) m/z [M+H]^+^ calcd for C_23_H_22_N_4_O_4_S 451.1435, found 451.14375. HPLC: flow, 1 mL/min; isocratic H_2_O 20%/CH_3_CN 80% for 12 min; ret. time: 4.7533 min.

#### 4.1.44 6‐Ethyl‐2‐[(4‐nitrobenzoyl)amino]‐*N*‐pyridin‐2‐yl‐4,5,6,7‐tetrahydrothieno[2,3‐*c*]pyridine‐3‐carboxamide (12)

The title compound was prepared starting from **43** (1.24 mmol, 0.50 g) through Method C (reaction time: 7 h) and obtained as a yellow solid in 77% yield (0.46 g) purified by flash chromatography eluting with CH_2_Cl_2_/MeOH (95:5). ^1^H NMR (400 MHz, DMSO‐*d*_6_): δ 1.34 (t, *J* = 7.2 Hz, 3H, CH_2_ *CH*_3_), 3.21–3.49 (m, 6H, *CH*_2_CH_3_ and piperidinyl‐CH_2_ x2), 4.22.4.38 (m, 2H, piperidinyl‐CH_2_), 7.02–7.08 (m, 1H, Ar‐H), 7.76 (dt, *J* = 2.0 and 8.2 Hz, 1H, Ar‐H), 8.26–8.34 (m, 3H, Ar‐H), 8.47–8.52 (m, 1H, Ar‐H), 8.71 (d, *J* = 8.3 Hz, 2H, Ar‐H), 9.68 (bs, 1H, NH), 14.19 (bs, 1H, NH). ^13^C NMR (101 MHz, DMSO‐*d*_6_): δ 9.86, 24.99, 49.76, 50.07, 50.94, 113.51, 113.96, 114.41, 118.64, 123.47, 129.55, 130.06, 138.34, 145.68, 148.80, 149.15, 153.83, 159.53, 164.32, 165.79. HRMS (ESI) m/z [M+H]^+^ calcd for C_22_H_21_N_5_O_4_S, 452.1387, found 452.13922. HPLC: flow, 0.8 mL/min; isocratic H_2_O with 0.1% formic acid 10%/CH_3_CN 50%/MeOH 40% for 10 min; ret. time: 2.2967 min.

#### 4.1.45 *tert*‐Butyl 2‐[(4‐nitrobenzoyl)amino]‐3‐[(pyridin‐2‐ylamino)carbonyl]−4,7‐dihydrothieno[2,3‐*c*]pyridine‐6(5*H*)‐carboxylate (46)

The title compound was prepared starting from **44** (0.53 mmol, 0.25 g) through Method C (reaction time: 12 h) and obtained as a red solid in 82% yield (0.23 g) after crystallization by EtOH/DMF. ^1^H NMR (400 MHz, CDCl_3_): δ 1.54 (s, 9H, C(CH_3_)_3_), 3.09–3.12 (m, 2H, piperidinyl‐CH_2_), 3.80–3.84 (m, 2H, piperidinyl‐NCH_2_), 4.63–4.65 (m, 2H, piperidinyl‐NCH_2_), 7.13–7.41 (m, 1H, Ar‐H), 7.79–7.84 (m, 1H, Ar‐H), 8.22 (d, *J* = 8.1 Hz, 2H, Ar‐H), 8.28 (bs, 1H, NH), 8.32–8.34 (m, 1H, Ar‐H), 8.35–8.37 (m, 1H, Ar‐H), 8.40 (d, *J* = 8.1 Hz, 2H, Ar‐H), 12.53 (bs, 1H, NH).

#### 4.1.46 2‐[(4‐Nitrobenzoyl)amino]‐*N*‐pyridin‐2‐yl‐4,5,6,7‐tetrahydrothieno[2,3‐*c*]pyridine‐3‐carboxamide (13)

Under N_2_ atmosphere, to a solution of compound **46** (0.96 mmol, 0.50 g) in dry CH_2_Cl_2_ (14 mL), trifluoroacetic acid (2.87 mmol, 0.22 mL) was added dropwise, maintaining the temperature at 0°C. After 3 h at 0°C, the mixture was concentrated to dryness and diluted with H_2_O (pH = 8). The obtained precipitate was filtered to give compound **13** as an orange solid in 81% yield (0.33 g) after crystallization by EtOH/DMF. ^1^H NMR (400 MHz, DMSO‐*d*_6_): δ 3.20–3.29 (m, 2H, piperidinyl‐CH_2_), 3.34 (s, 1H, NH), 3.36–3.46 (m, 2H, piperidinyl‐NCH_2_), 4.19–4.29 (m, 2H, piperidinyl‐NCH_2_), 7.04–7.07 (m, 1H, Ar‐H), 7.78 (t, *J* = 7.3 Hz, 1H, Ar‐H), 8.27–8.38 (m, 3H, Ar‐H), 8.50–8.56 (m, 1H, NH), 8.74 (d, *J* = 7.5 Hz, 2H, Ar‐H), 8.89–9.11 (m, 2H, Ar‐H and NH), 14.30 (bs, 1H, NH). ^13^C NMR (101 MHz, DMSO‐*d*_6_): δ 24.29, 41.74, 42.35, 113.33, 114.02, 114.79, 118.59, 123.47, 129.60, 129.98, 138.37, 145.58, 148.59, 149.17, 153.70, 159.08, 164.25, 165.57. HRMS (ESI) m/z [M+H]^+^ calcd for C_20_H_17_N_5_O_4_S 424.1074, found 424.10767. HPLC: flow, 0.5 mL/min; gradient, from 80% H_2_O with 0.01% formic acid and 1 mM ammonium formate and 20% CH_3_CN to 100% CH_3_CN in 12 min followed by 3 min at 100% CH_3_CN; ret. time: 3.633 min.

#### 4.1.47 2‐[(4‐Nitrobenzoyl)amino]‐*N*‐pyridin‐2‐yl‐1‐benzothiophene‐3‐carboxamide (14)

The title compound was prepared starting from **45** (0.54 mmol, 0.20 g) through Method C (reaction time: 6 h) and obtained as a yellow solid in 79% yield (0.18 g) after crystallization by EtOH/DMF. ^1^H NMR (400 MHz, DMSO‐*d*_6_): δ 7.18–7.26 (m, 1H, Ar‐H), 7.32–7.41 (m, 1H, Ar‐H), 7.43–7.51 (m, 1H, Ar‐H), 7.87–8.09 (m, 3H, Ar‐H), 8.18–8.35 (m, 3H, Ar‐H), 8.38–8.51 (m, 3H, Ar‐H), 10.87 (bs, 1H, NH), 12.08 (bs, 1H, NH). ^13^C NMR (101 MHz, DMSO‐d_6_): δ 115.50, 120.40, 122.71, 124.36, 125.67, 129.63, 130.14, 134.59, 134.93, 138.82, 138.96, 144.08, 148.24, 148.37, 150.19, 152.60, 152.66, 163.57, 163.72. HRMS (ESI) m/z [M+H]^+^ calcd for C_21_H_14_N_4_O_4_S 419.0809, found 419.08186. HPLC: flow, 0.8 mL/min; isocratic H_2_O 20%/CH_3_CN 80% for 10 min; ret. time: 3.0500 min.

#### 4.1.48 Ethyl 3‐[(4‐nitrobenzoyl)amino]‐1‐benzothiophene‐2‐carboxylate (48)

The title compound was prepared starting from compound **47** [33] (2.71 mmol, 0.60 g) through Method B (reaction time: 6 h) as a yellow solid in 66% yield (0.66 g). ^1^H NMR (400 MHz, CDCl_3_): δ 1.46 (t, *J* = 6.4 Hz, 3H, OCH_2_ *CH*_3_), 4.45 (q, *J* = 6.4 Hz, 2H, O*CH*_2_CH_3_), 7.47 (t, *J* = 7.0 Hz, 1H, Ar‐H), 7.55 (t, *J* = 7.0 Hz, 1H, Ar‐H), 7.84 (d, *J* = 7.8 Hz, 1H, Ar‐H), 8.25–8.30 (m, 3H, Ar‐H), 8.42 (d, *J* = 7.5 Hz, 2H, Ar‐H), 10.78 (s, 1H, NH).

#### 4.1.49 3‐[(4‐Nitrobenzoyl)amino]‐*N*‐pyridin‐2‐yl‐1‐benzothiophene‐2‐carboxamide (15)

The title compound was prepared starting from **48** (0.27 mmol, 0.10 g) through Method C (reaction time: 12 h) and obtained as a yellow solid in 86% yield (0.10 g) after crystallization by EtOH/DMF. ^1^H NMR (400 MHz, DMSO‐*d*_6_): δ 7.16 (t, *J* = 6.6 Hz, 1H, Ar‐H), 7.52 (t, *J* = 7.1 Hz, 1H, Ar‐H), 7.58 (t, *J* = 7.2 Hz, 1H, Ar‐H), 7.84 (t, *J* = 8.3 Hz, 1H, Ar‐H), 7.91 (d, *J* = 8.4 Hz, 1H, Ar‐H), 8.11 (d, *J* = 8.1 Hz, 2H, Ar‐H), 8.26–8.36 (m, 3H, Ar‐H), 8.43 (d, *J* = 8.5 Hz, 2H, Ar‐H), 10.42 (bs, 1H, NH), 10.97 (bs, 1H, NH). ^13^C NMR (101 MHz, DMSO‐*d*_6_): δ 114.75, 120.76, 123.69, 124.27, 125.56, 127.75, 130.01, 130.55, 131.21, 136.14, 137.67, 138.97 (2C), 139.93, 148.77, 150.07, 151.79, 160.88, 165.72. HRMS (ESI) m/z [M+H]^+^ calcd for C_21_H_14_N_4_O_4_S 419.0809, found 419.08074. HPLC: flow, 0.8 mL/min; isocratic H_2_O with 0.1% formic acid 30%/CH_3_CN 70% for 10 min; ret. time: 3.1000 min.

#### 4.1.50 2‐Amino‐*N*‐phenylbenzamide (51)

A solution of acid derivative **49** (1.82 mmol, 0.25 g) in SOCl_2_ (2 mL) was stirred at reflux for 4 h. Then, the excess of SOCl_2_ was evaporated under vacuum to obtain the acyl chloride derivative **50** as a yellow oil in a quantitative yield [44]. Subsequently, under N_2_ atmosphere, compound **50** (1.82 mmol, 0.28 g) was added dropwise to a solution of 2‐aminopyridine (1.82 mmol, 0.17 g) in dry pyridine (6 mL) and the mixture was stirred at rt for 3 h. Then, it was poured into ice/water and extracted with EtOAc (x3). The organic layer was washed with brine, dried over Na_2_SO_4_, and evaporated to dryness to give a yellow oil. After purification by chromatography column eluting with cyclohexane/EtOAc (70:30), compound **51** was obtained as a yellow solid in 61% yield (0.24 g). ^1^H NMR (400 MHz, DMSO‐*d*_6_): δ 6.45 (bs, 2H, NH_2_), 6.56 (t, *J* = 7.1 Hz, 1H, Ar‐H), 6.76 (d, *J* = 8.3 Hz, 1H, Ar‐H), 7.14 (dt, *J* = 1.0 and 7.2 Hz, 1H, Ar‐H), 7.21 (t, *J* = 7.1 Hz, 1H, Ar‐H), 7.74 (d, *J* = 8.0 Hz, 1H, Ar‐H), 7.82 (t, *J* = 7.1 Hz, 1H, Ar‐H), 8.09 (d, *J* = 7.4 Hz, 1H, Ar‐H), 8.36–8.38 (m, 1H, Ar‐H), 10.40 (bs, 1H, NH).

#### 4.1.51 2‐[(4‐Nitrobenzoyl)amino]‐*N*‐pyridin‐2‐ylbenzamide (16)

The title compound was prepared starting from **51** (1.82 mmol, 0.28 g) through Method B (reaction time: 5 h) and obtained as a white solid in 85% yield (0.18 g) after purification by flash chromatography eluting with cyclohexane/EtOAc (70:30). ^1^H NMR (400 MHz, DMSO‐*d*_6_): δ 7.20–7.26 (m, 1H, Ar‐H), 7.37 (t, *J* = 7.4 Hz, 1H, Ar‐H), 7.70 (t, *J* = 7.3 Hz, 1H, Ar‐H), 7.89 (t, *J* = 8.5 Hz, 1H, Ar‐H), 8.00 (dd, *J* = 1.0 and 7.8 Hz, 1H, Ar‐H), 8.60 (d, *J* = 8.3 Hz, 1H, Ar‐H), 8.20 (d, *J* = 8.7 Hz, 2H, Ar‐H), 8.28 (d, *J* = 8.0 Hz, 1H, Ar‐H), 8.42–8.47 (m, 3H, Ar‐H), 11.00 (bs, 1H, NH), 11.57 (bs, 1H, NH). ^13^C NMR (101 MHz, DMSO‐d_6_): δ 115.81, 120.59, 122.95, 124.42, 124.70, 125.05, 129.25, 129.96, 132.68, 138.03, 138.56, 140.66, 148.49, 149.82, 152.25, 163.85, 168.02. HPLC: flow, 1 mL/min; isocratic H_2_O with 0.1% formic acid 30%/CH_3_CN 70% for 10 min; ret. time: 2.787 min.

### 4.2 Biological Studies

#### 4.2.1 Compounds for Biological Studies

RBV (1‐D‐ribofuranosyl‐1,2,4‐triazole‐3‐carboxamide) was purchased from Roche. FPV was purchased from MedChemExpress. All test compounds were dissolved in 100% DMSO. The PB1_1–15_–Tat peptide was synthesized and purified by the Peptide Facility of CRIBI Biotechnology Center (University of Padua, Padua, Italy). All stock solutions were stored at −20°C.

#### 4.2.2 Cells and Viruses

(MDCK [NBL‐2], CCL‐34, RRID:CVCL_0422) and HEK 293T cells (CRL‐3216, RRID:CVCL_0063) were purchased from American Type Culture Collection (ATCC) and were grown in Dulbecco's modified Eagle's medium (DMEM, Life Biotechnologies) supplemented with 10% (v/v) fetal bovine serum (FBS, Life Technologies) and antibiotics (100 U/mL penicillin and 100 μg/mL streptomycin, Life Technologies). Cells were maintained at 37°C in a humidified atmosphere with 5% CO_2_.

The IAV/PR/8/34 (H1N1) was kindly provided by P. Digard (Roslin Institute, University of Edinburgh, United Kingdom). The IAV/Wisconsin/67/05 and IBV/Malaysia/2506/4 strains were provided by R. Cusinato (Microbiology and Virology Unit, Padua University Hospital, Italy). The IBV/Lee/40 strain was obtained from W. S. Barclay (Imperial College, London, United Kingdom). The clinical isolate A/Parma/24/09 was kindly provided by I. Donatelli (Istituto Superiore di Sanità, Rome, Italy). All viruses were propagated and titrated in MDCK cells at 37°C and 5% CO_2_.

#### 4.2.3 Cytotoxicity Assays

Cytotoxicity of test compounds was tested in MDCK and HEK 293T cells by the 3‐(4,5‐dimethylthiazol‐2‐yl)‐2,5‐diphenyl tetrazolium bromide (MTT) method, as previously reported [46] with minor modifications. Briefly, MDCK or HEK 293T cells were cultured in 96‐well plates at 37°C for 48 h in DMEM supplemented with 10% FBS containing different concentrations of test compounds or DMSO as a solvent control. Then, MTT solution (5 mg/mL in PBS) was added to each well, and the plates were incubated for 4 h at 37°C. Successively, a solubilization solution (10% SDS, 0.01N HCl) was added to lyse cells. After overnight incubation at 37°C, absorbance was read at 570 nm using a plate reader (Multiskan FC Microplate Photometer, Thermo‐Fisher Scientific, USA).

#### 4.2.4 PRAs

PRAs were performed as previously reported [46] with some modifications. MDCK cells were seeded at 5 × 10^5^ cells/well into 12‐well plates and incubated at 37°C for 24 h. The following day, the culture medium was removed, and the monolayers were first washed with serum‐free DMEM, then infected with 40 PFU of the different IAV and IBV strains in DMEM supplemented with 2 μg/mL of TPCK‐treated trypsin (Worthington Biochemical Corporation), 0.14% BSA, and test compounds at different concentrations (or solvent as a control), and incubated for 1 h at 37°C. Then, serum‐free medium containing 2 μg/mL of TPCK‐treated trypsin, 0.14% BSA, 1.2% Avicel, and test compounds at different concentrations or solvent as a control was added. After 48 h of incubation, cell monolayers were fixed with 4% formaldehyde and stained with 0.01% toluidine blue. Viral plaques were counted, and the EC_50_ of each compound was determined from the dose–response curves obtained by the nonlinear regression function of GraphPad Prism software version 10.

#### 4.2.5 Minireplicon Assays

The minireplicon assay was performed as described [47], with some modifications. Briefly, HEK 293T cells (2 × 10^5^ cells per well) were plated into 24‐well plates and incubated overnight at 37°C. The next day, cells were transfected using the calcium phosphate coprecipitation method with pcDNA‐PB1, pcDNA‐PB2, pcDNA‐PA, and pcDNA‐NP plasmids (100 ng/well of each) along with 50 ng/well of the pPolI‐Flu‐ffLuc reporter plasmid and 50 ng/well of pRL‐SV40 plasmid as a transfection control. Transfections were performed in the presence of different concentrations of test compounds or DMSO as a negative control. RBV was used as a positive control for inhibition. Cell medium was removed 4 h post‐transfection and replaced with DMEM containing test compounds, RBV, or DMSO. At 24 h post‐transfection, cells were harvested, lysed, and both firefly and *Renilla* luciferase activity were measured using the Dual Luciferase Assay Kit (Promega). In each experiment, firefly luciferase activity was normalized with that of the *Renilla* luciferase, and relative luciferase units (RLU) were obtained. The activity measured in control transfection reactions containing DMSO was set at 100% of polymerase activity.

#### 4.2.6 ELISA‐Based PA/PB1 Interaction Assay

The PA–PB1 interaction was detected by a procedure previously described [48], with some modifications. Briefly, 96‐well microtiter plates (Nuova Aptaca) were coated with 400 ng of 6His‐PA_(239–716)_ for 3 h at 37°C and then blocked with 2% BSA (Sigma) in PBS for 1 h at 37°C. After washing, 200 ng of GST‐PB1_(1–25)_, or GST alone as a control, were added and incubated in serum‐free DMEM in the absence or the presence of test compounds at various concentrations O/N at rt. After washing, the interaction between 6His‐PA_(239–716)_ and GST‐PB1_(1–25)_ was detected with a horseradish peroxidase‐coupled anti‐GST monoclonal antibody (GenScript) diluted 1:3,000 in PBS supplemented with 2% FBS. Following washes, the substrate 3,3′, 5, 5′tetramethylbenzidine (TMB, KPL) was added, and absorbance was measured at 450 nm by an ELISA plate reader (MultiSkan FC, Thermo‐Fisher). Values obtained from the samples treated with DMSO were used to set as 100% of PA–PB1 interaction.

### 4.3 Statistical Analysis

Data analysis was performed with GraphPad Prism version 10.5. Data in tables are presented as mean ± standard deviation (SD). The exact *n* value for each experiment is described in the table legends.

### 4.4 Thermodynamic Solubility Assessment

Thermodynamic solubility values were measured in aqueous media (pH = 7) using the shake flask method. One mg of each compound was placed in 1 mL of Milli‐Q water, and the suspensions were shaken at rt for 24 h. The resulting suspensions were centrifuged at 14 000 × g for 10 min using a microcentrifuge (Eppendorf 5425/5425 R, Hamburg, Germany). 200 µL of each supernatant was taken and diluted with 200 µL of MeOH (1:1 dilution). The resulting solutions were analyzed at *λ*_max_ of each compound using HPLC with a Jasco LC‐4000 instrument equipped with a UV–visible Diode Array Jasco MD‐4015 (Jasco Corporation, Tokyo, Japan) and a Gemini LC C18 110 Å column, 3 µm, 100 mm × 2 mm (Phenomenex, Torrance, CA, USA). Chromatographic separation was performed using a gradient elution with CH_3_CN:H_2_O with formic acid 0.1%_v/v_, eluting from 20 to 100% of CH_3_CN in 10 min at 0.5 mL/min. The measured values were corrected for the corresponding dilution factor and interpolated from calibration curves prepared using concentrated DMSO stock solutions of each compound, which were diluted with Milli‐Q water to a final concentration of 10, 50, and 100 µM (final DMSO concentration 5%). A solution of each compound at a known concentration was used as a positive control. The compounds were tested in duplicate.

### 4.5 In Silico Studies

#### 4.5.1 Protein Preparation

The PA_C_–PB1_N_ complex (PDB ID: 3CM8) was downloaded from Protein Data Bank [49] and submitted to Schrödinger's Protein Preparation Wizard [50] to obtain a suitable starting structure for computational studies. All water molecules were removed, hydrogen atoms were added, and bond orders were assigned to amino acid residues and ligands. Terminal groups were capped with neutral blocking groups (acetyl (ACE) at the N–terminus and N–methylamide (NME) at the C–terminus) to neutralize free chain ends and avoid artefactual electrostatic interactions during the simulations. Epik [50] was then used to predict ionization and tautomeric states for the ligands using a pH of 7.0 ± 1.0. Optimization of the hydrogen‐bonding network was performed, including adjustment of side‐chain orientations and tautomeric states. In addition, the ionization and tautomeric states of His, Asp, Glu, Arg, and Lys were adjusted to match a pH of 7.4. The systems were then subjected to restrained minimization using the OPLS4 force field, and the procedure was halted when the heavy‐atom root mean square deviation (RMSD) converged to the default limit of 0.30 Å.

#### 4.5.2 SiteMap

The prepared protein was submitted to siteMap analysis. Starting from the PA_C_–PB1_N_ complex, the PB1_N_ portion was removed to allow a comprehensive analysis of the interaction region [51, 52]. SiteMap was run with default settings, using the more restrictive definition of hydrophobicity and a standard grid. The pocket detected in the interaction region between PA and PB1 was characterized by favorable scores (Sitescore 0.991; Dscore; 1.041) and defined by the residues 408, 411, 415, 620, 621, 622, 623, 628, 635, 638, 639, 706, 709, 710, and 713.

#### 4.5.3 Docking Studies

Docking studies were performed by using AutoDock [53]. Ligand and receptor structures were converted to AD4 format files using AutoDockTools [53], and then, the Gasteiger–Marsili partial charges were assigned. The dimension of the grid was 60 × 60 × 60 (grid points separated by a 0.375 Å). The grid was centered on the position of the binding site detected in the siteMap analysis (x:63.747; y:17.486; z:46.440). The Lamarckian genetic algorithm local search method was used, and for each compound, the docking simulation was composed of 100 runs. Clustering of docked conformations was performed on the basis of their RMSD (tolerance = 2.0 Å), and the results were ranked based on the estimated free energy of binding.

## Funding

This study was supported by NextGeneration EU‐MUR PNRR Extended Partnership initiative on Emerging Infectious Diseases (PE00000007), Associazione Italiana per la Ricerca sul Cancro (25899), Fondazione Cassa di Risparmio di Padova e Rovigo (55777 2020.0162).

## Conflicts of Interest

The authors declare no conflicts of interest.

## Supporting information

Supplementary Material

## Data Availability

The data that support the findings of this study are available from the corresponding author upon reasonable request.
