# Supplementary material for: Streamlined Synthesis and Structure–Activity Relationship Analysis of 2‐Amidothiophene‐3‐Carboxamides Targeting Influenza Polymerase PA‐PB1 Heterodimerization
Source: ChemMedChem. 2026 May 22;21(10):e70314. doi: 10.1002/cmdc.70314 (PMC13206478; doi:10.1002/cmdc.70314)
Supplement: Supplementary file 1 — Supplementary Material [file CMDC-21-e70314-s001.pdf]

Supporting Information

Streamlined Synthesis and SAR Analysis of 2-Amidothiophene-3-Carboxamides Targeting Influenza Polymerase PA–PB1 Heterodimerization

Tommaso Felicetti,[a] Alessia Zago,[b] Andrea Astolfi,[a] Giuseppe Manfroni,[a] Stefano Sabatini,[a] Maria Letizia Barreca,[a] Oriana Tabarrini,[a] Arianna Loregian,[b,c,*] and Serena Massari.[a,*]

[a] Department of Pharmaceutical Sciences, University of Perugia, Via Del Liceo 1, 06123, Perugia, Italy

[b] Department of Molecular Medicine, University of Padua, Via Gabelli 63, 35121, Padua, Italy

[c] Microbiology and Virology Unit, Padua University Hospital, Via Giustiniani 2, 35128, Padua, Italy

*Corresponding authors. Email address: S.M.: serena.massari@unipg.it; A.L. arianna.loregian@unipd.it

Table of Contents

Figures S1-S15. Chromatograms of HPLC analyses of target compounds **2-16**. Pag. S3-10

Figures S16-S44. ^{1}H NMR and ^{13}C NMR spectra of target compounds **2-16**. Pag. S11-S25

Figures S45-S58. HRMS spectra of target compounds **3-15**. Pag. S26-S28

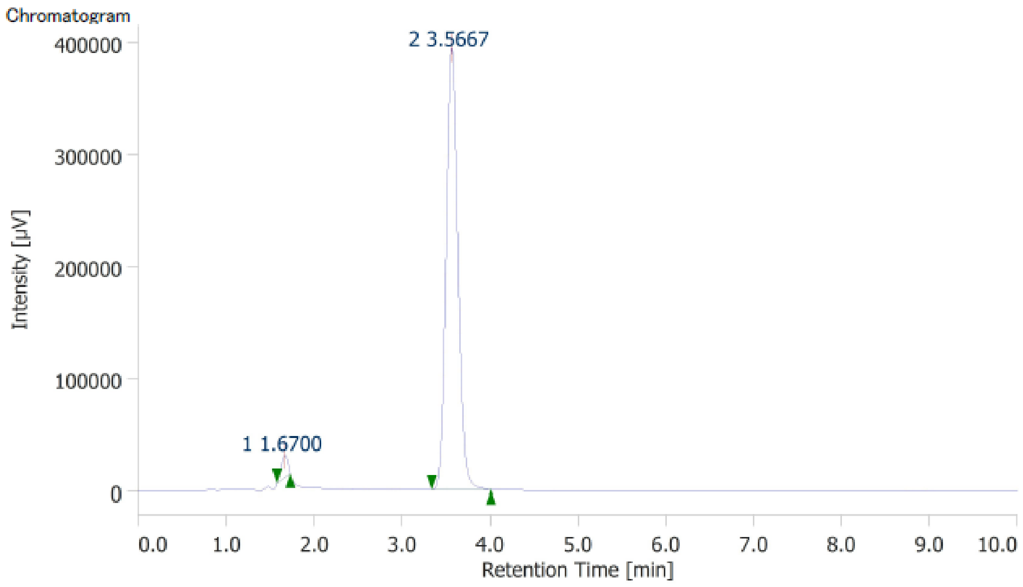

Peak Information

#	Peak Name	CH	tR [min]	Area [µV·sec]	Height [µV]	Area%	Height%	Quantity
1	Peak-001	5	1.670	108396	19769	2.880	4.780	N/A
2	Peak-002	5	3.567	3654831	393827	97.120	95.220	N/A

Figure S1. Chromatography analysis of compound **2**.

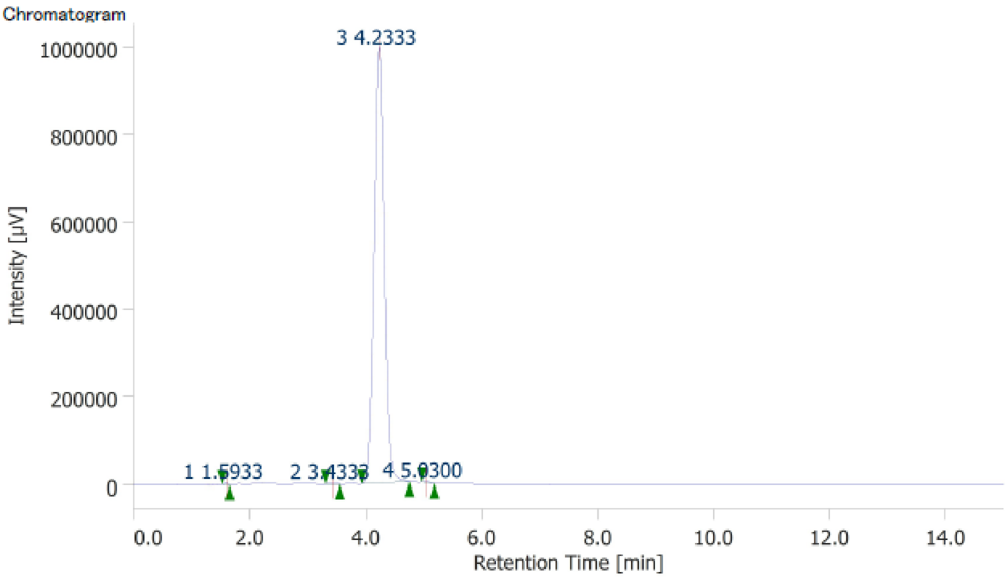

Peak Information

#	Peak Name	CH	tR [min]	Area [µV·sec]	Height [µV]	Area%	Height%	Quantity
1	Peak-001	5	1.593	13291	3567	0.109	0.355	N/A
2	Peak-002	5	3.433	12862	1733	0.106	0.172	N/A
3	Peak-003	5	4.233	12137689	998259	99.694	99.339	N/A
4	Peak-004	5	5.030	11058	1340	0.091	0.133	N/A

Figure S2. Chromatography analysis of compound **3**.

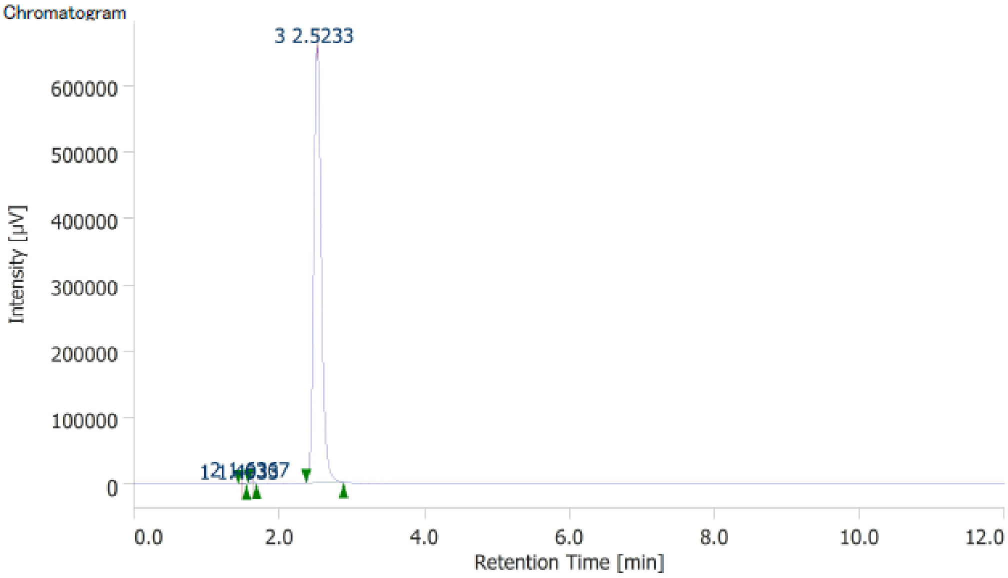

Peak Information

#	Peak Name	CH	tR [min]	Area [µV·sec]	Height [µV]	Area%	Height%	Quantity
1	Peak-001	5	1.493	5194	1498	0.110	0.226	N/A
2	Peak-002	5	1.637	11605	3457	0.247	0.520	N/A
3	Peak-003	5	2.523	4688882	659508	99.643	99.254	N/A

Figure S3. Chromatography analysis of compound **4**.

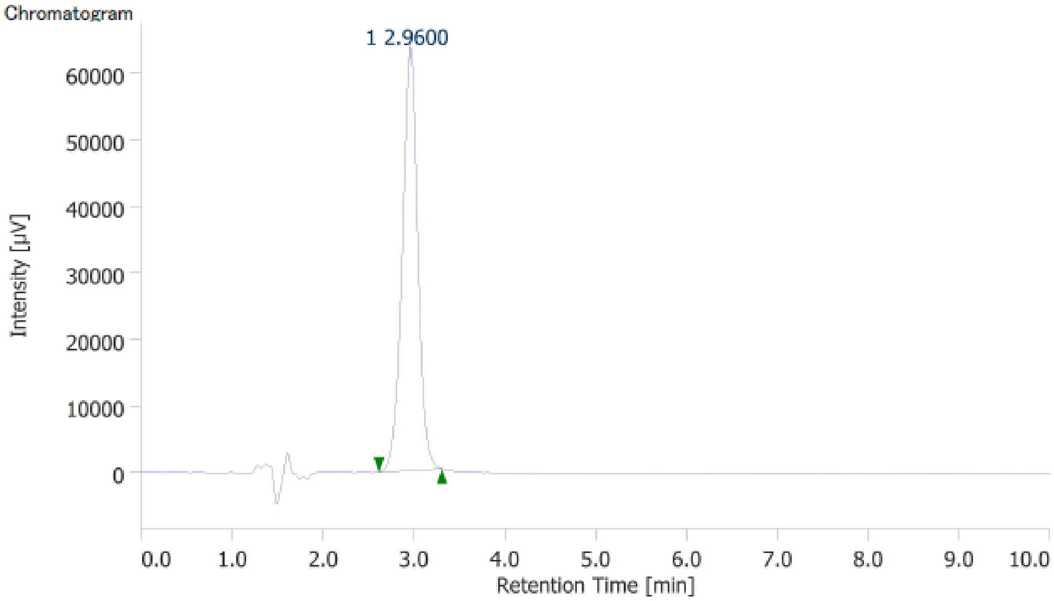

Peak Information

#	Peak Name	CH	tR [min]	Area [µV·sec]	Height [µV]	Area%	Height%	Quantity
1	Peak-001	5	2.960	758675	63685	100.000	100.000	N/A

Figure S4. Chromatography analysis of compound **5**.

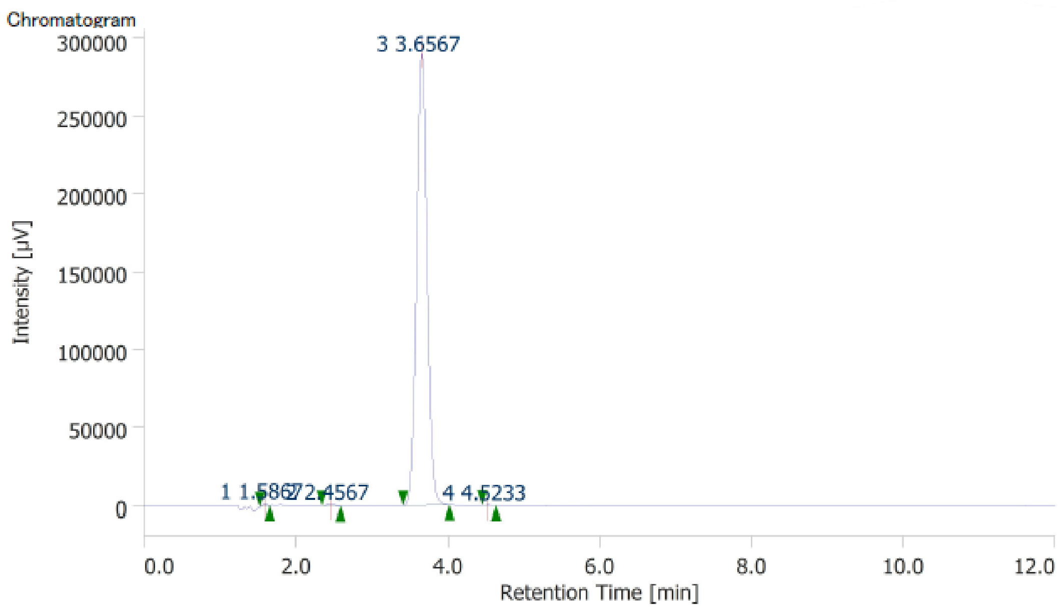

Peak Information

#	Peak Name	CH	tR [min]	Area [μV·sec]	Height [μV]	Area%	Height%	Quantity
1	Peak-001	5	1.587	6581	1723	0.233	0.589	N/A
2	Peak-002	5	2.457	6232	845	0.220	0.289	N/A
3	Peak-003	5	3.657	2812583	289875	99.510	99.069	N/A
4	Peak-004	5	4.523	1024	155	0.036	0.053	N/A

Figure S5. Chromatography analysis of compound **6**.

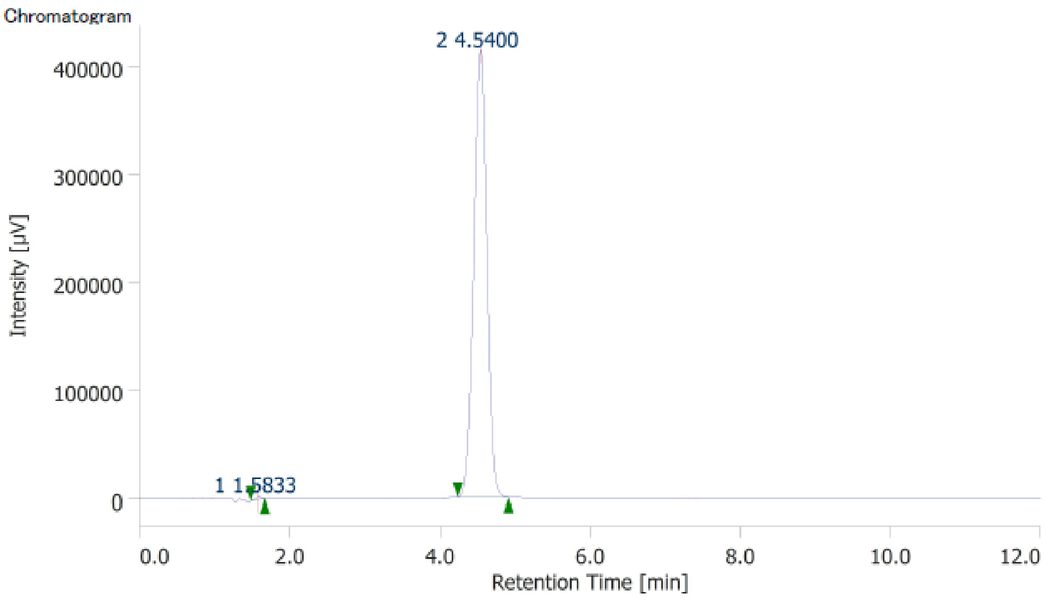

Peak Information

#	Peak Name	CH	tR [min]	Area [μV·sec]	Height [μV]	Area%	Height%	Quantity
1	Peak-001	5	1.583	12060	2312	0.240	0.554	N/A
2	Peak-002	5	4.540	5019809	414887	99.760	99.446	N/A

Figure S6. Chromatography analysis of compound **7**.

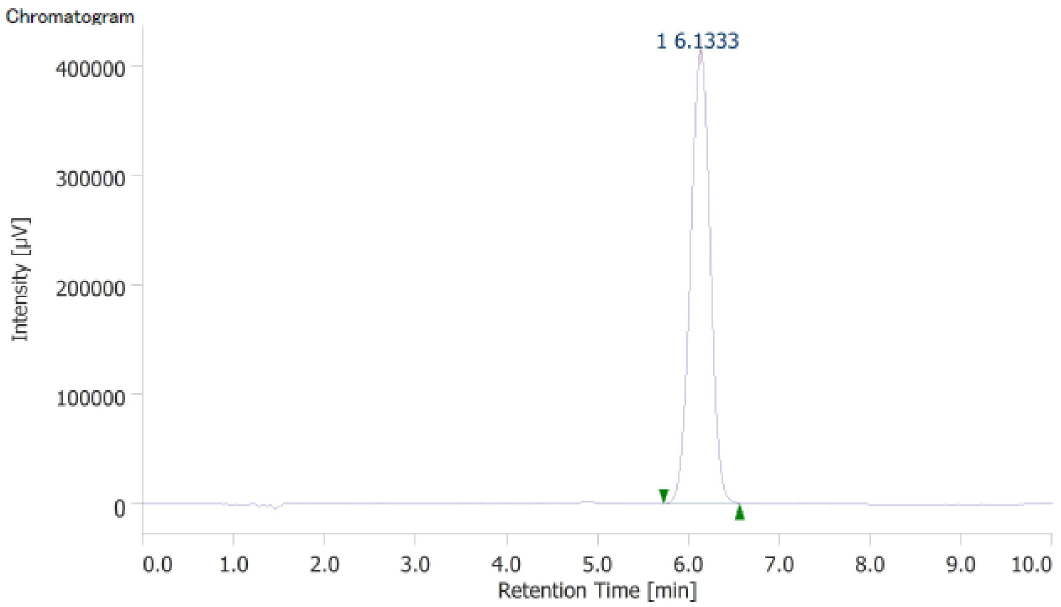

Peak Information

#	Peak Name	CH	tR [min]	Area [μV·sec]	Height [μV]	Area%	Height%	Quantity
1	Peak-001	5	6.133	6210280	413638	100.000	100.000	N/A

Figure S7. Chromatography analysis of compound **8**.

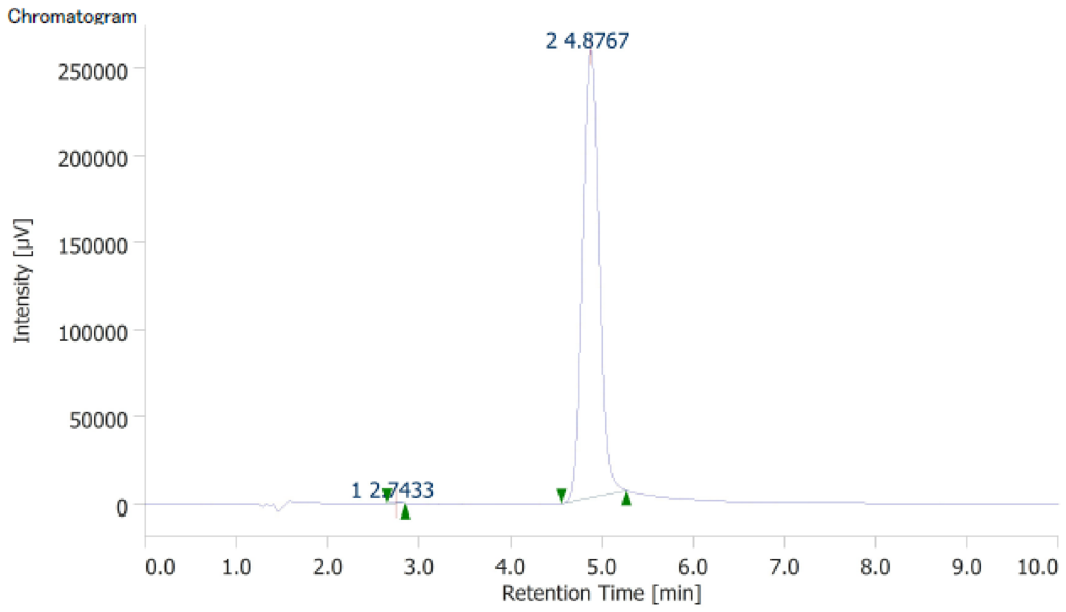

Peak Information

#	Peak Name	CH	tR [min]	Area [μV·sec]	Height [μV]	Area%	Height%	Quantity
1	Peak-001	5	2.743	5392	824	0.167	0.320	N/A
2	Peak-002	5	4.877	3214948	256928	99.833	99.680	N/A

Figure S8. Chromatography analysis of compound **9**.

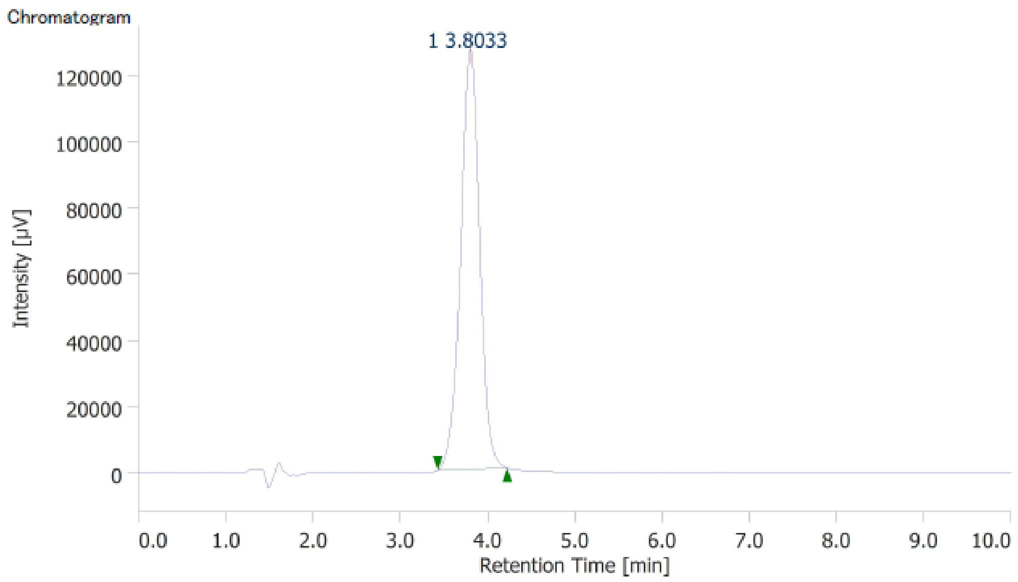

Peak Information

#	Peak Name	CH	tR [min]	Area [µV·sec]	Height [µV]	Area%	Height%	Quantity
1	Peak-001	5	3.803	1910824	126640	100.000	100.000	N/A

Figure S9. Chromatography analysis of compound **10**.

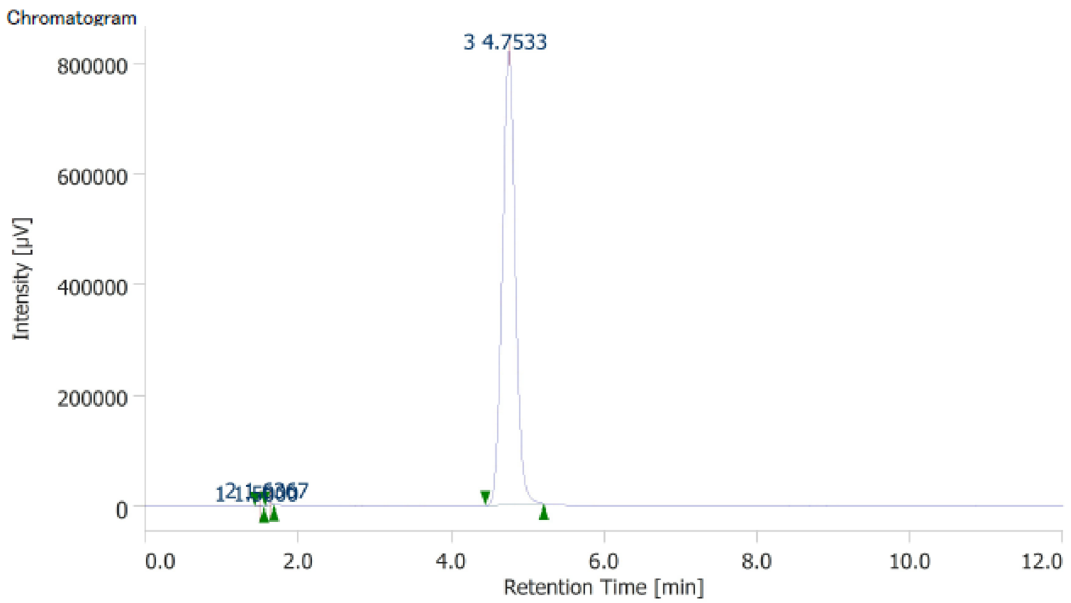

Peak Information

#	Peak Name	CH	tR [min]	Area [µV·sec]	Height [µV]	Area%	Height%	Quantity
1	Peak-001	5	1.500	6068	1780	0.063	0.215	N/A
2	Peak-002	5	1.637	19009	5629	0.197	0.681	N/A
3	Peak-003	5	4.753	9620242	819188	99.740	99.104	N/A

Figure S10. Chromatography analysis of compound **11**.

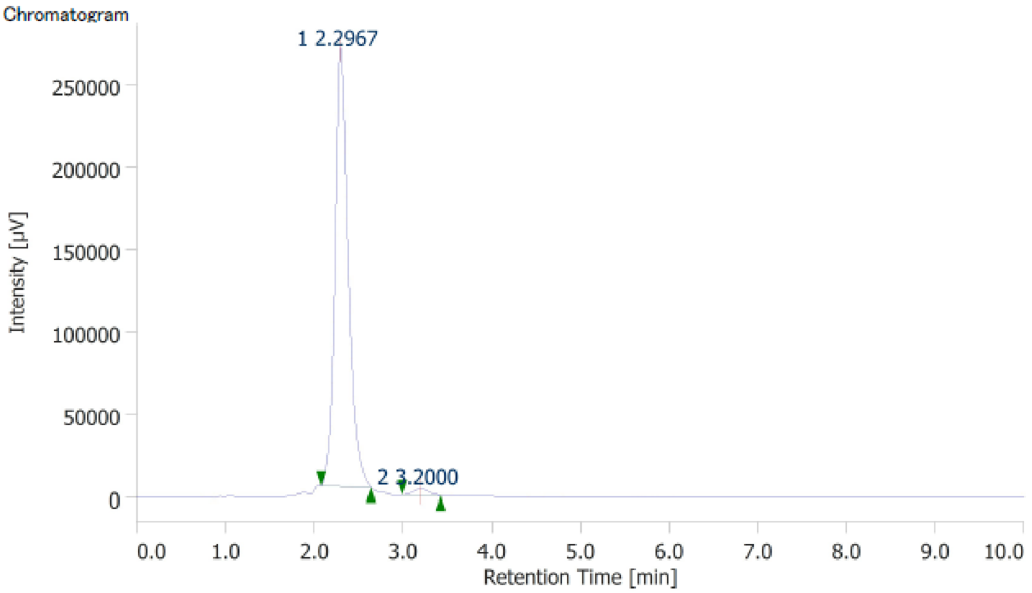

Peak Information

#	Peak Name	CH	tR [min]	Area [µV·sec]	Height [µV]	Area%	Height%	Quantity
1	Peak-001	5	2.297	2751143	266355	98.411	98.563	N/A
2	Peak-002	5	3.200	44421	3884	1.589	1.437	N/A

Figure S11. Chromatography analysis of compound **12**.

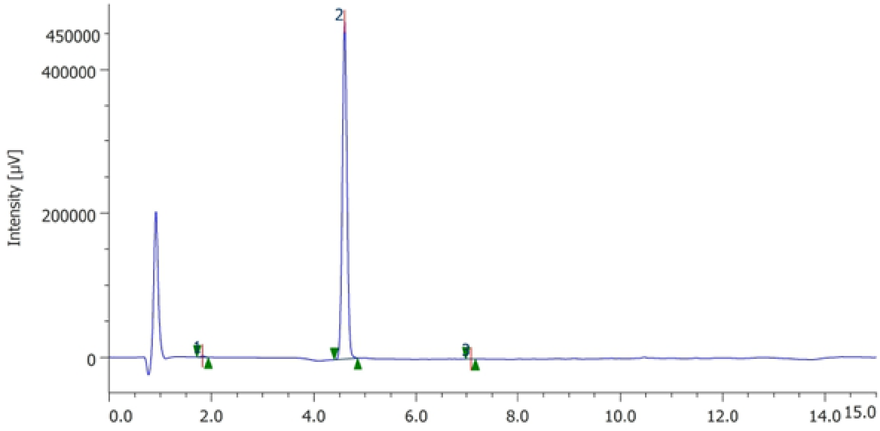

#	Peak Name	CH	tR [min]	Area [µV·sec]	Height [µV]	Area%	Height%	Quantity	NTP	Resolution	Symmetry Factor	Warning
1	Peak-001	5	1.827	9347	1549	0.316	0.329	N/A	1978	16.697	1.040	
2	Peak-002	5	4.593	2951700	468770	99.651	99.634	N/A	12544	16.399	1.017	
3	Peak-003	5	7.090	987	173	0.033	0.037	N/A	40309	N/A	0.888	

Figure S12. Chromatography analysis of compound **13**. The non-integrated peak at 1 minute corresponds to the DMSO that was used to dissolve the sample prior to injection.

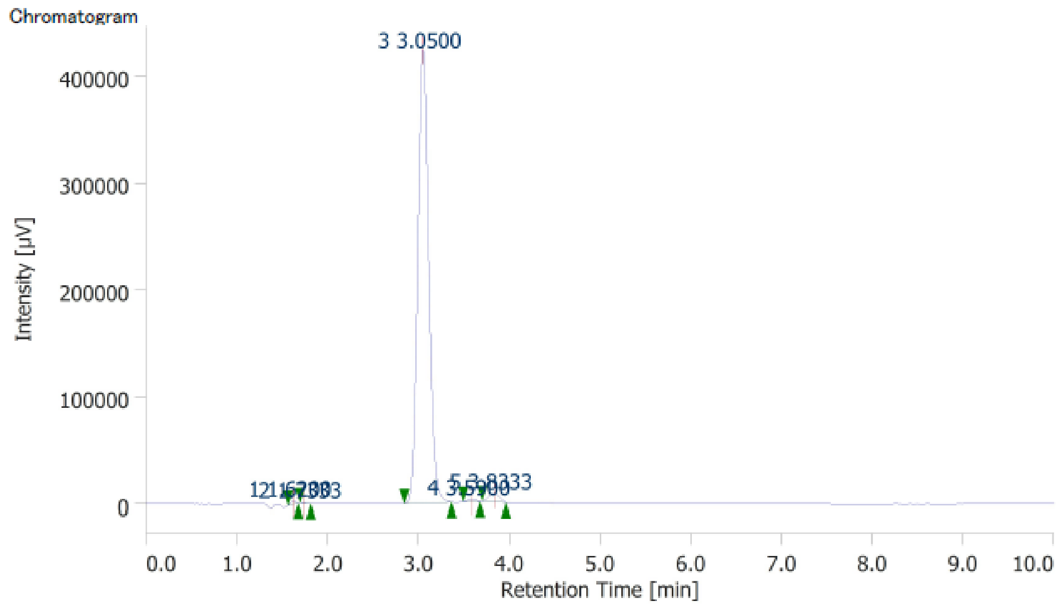

Peak Information

#	Peak Name	CH	tR [min]	Area [µV·sec]	Height [µV]	Area%	Height%	Quantity
1	Peak-001	5	1.620	11484	3061	0.321	0.701	N/A
2	Peak-002	5	1.733	2533	771	0.071	0.176	N/A
3	Peak-003	5	3.050	3487322	423694	97.603	96.986	N/A
4	Peak-004	5	3.590	10631	1702	0.298	0.390	N/A
5	Peak-005	5	3.833	61003	7632	1.707	1.747	N/A

Figure S13. Chromatography analysis of compound **14**.

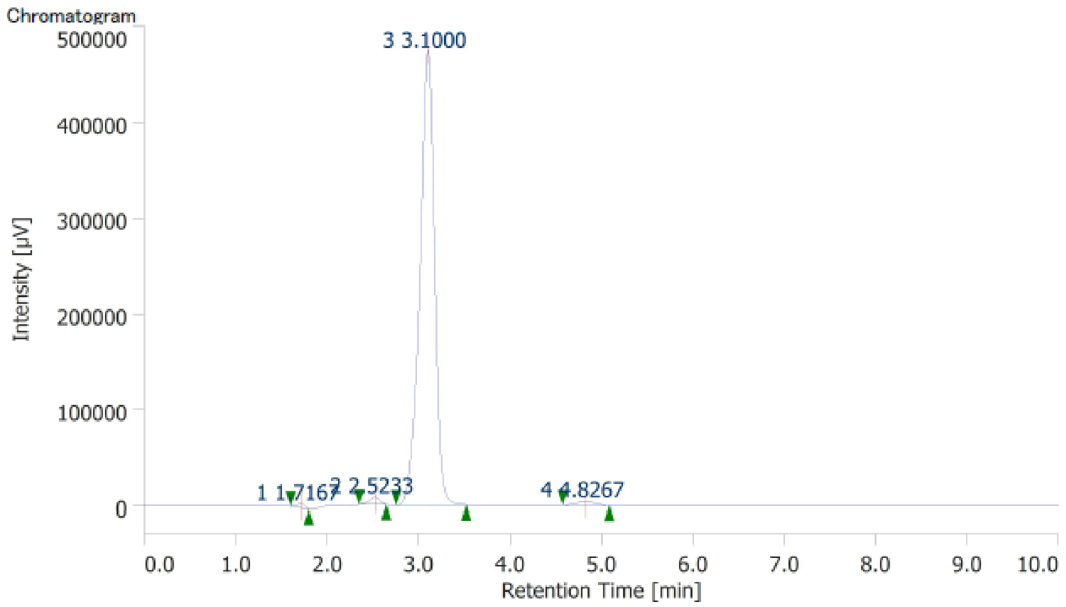

Peak Information

#	Peak Name	CH	tR [min]	Area [µV·sec]	Height [µV]	Area%	Height%	Quantity
1	Peak-001	5	1.717	26045	3655	0.483	0.745	N/A
2	Peak-002	5	2.523	56416	7168	1.046	1.461	N/A
3	Peak-003	5	3.100	5263022	476149	97.570	97.071	N/A
4	Peak-004	5	4.827	48625	3542	0.901	0.722	N/A

Figure S14. Chromatography analysis of compound **15**.

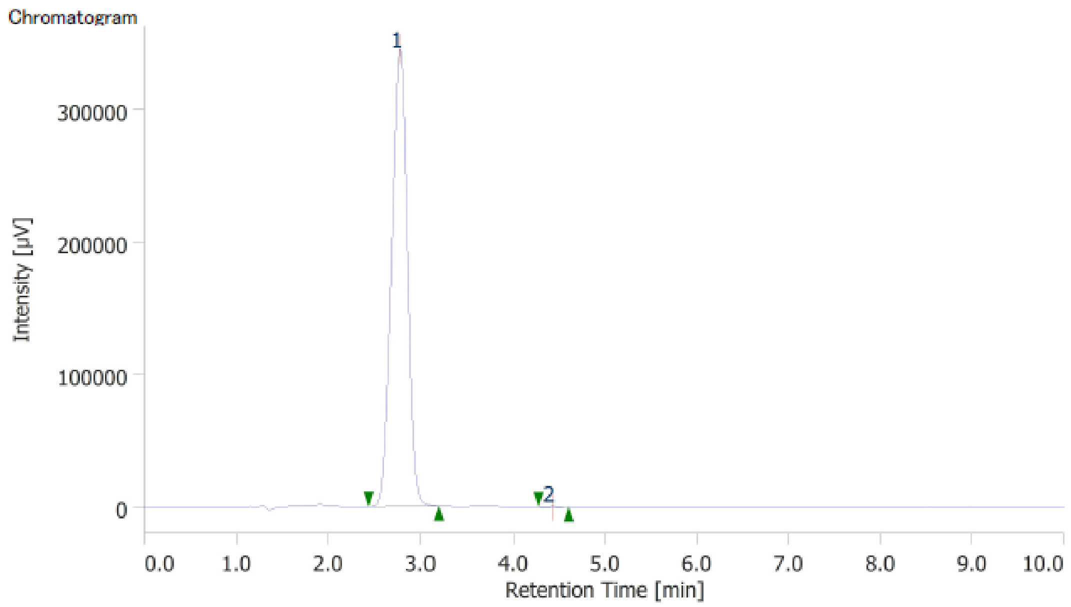

Peak Information

#	Peak Name	CH	tR [min]	Area [µV·sec]	Height [µV]	Area%	Height%	Quantity	Resolution
1	Peak-001	5	2.787	4097752	345203	99.902	99.900	N/A	5.049
2	Peak-002	5	4.427	4006	347	0.098	0.100	N/A	N/A

Figure S15. Chromatography analysis of compound **16**.

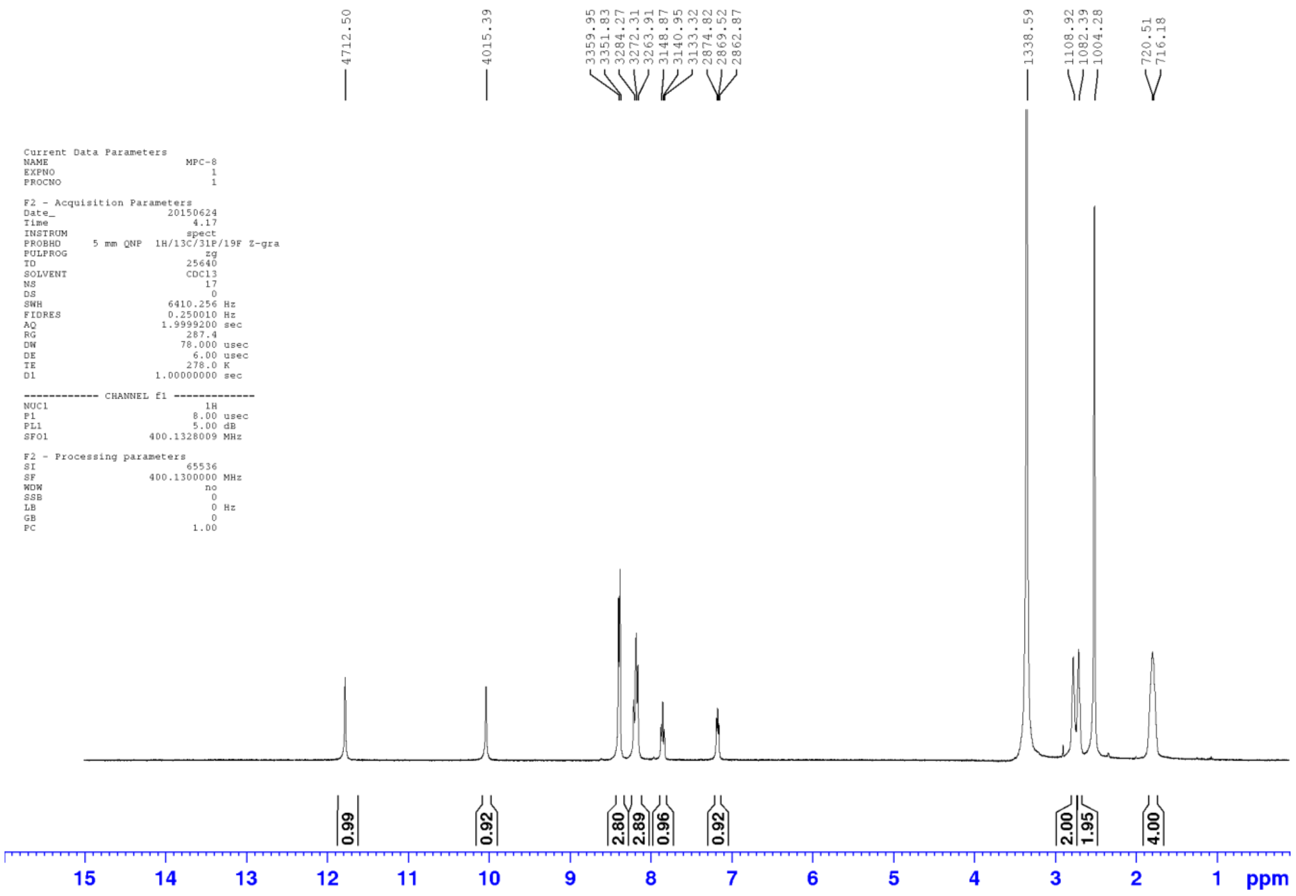

Figure S16. ^{1}H NMR spectrum of compound **2**.

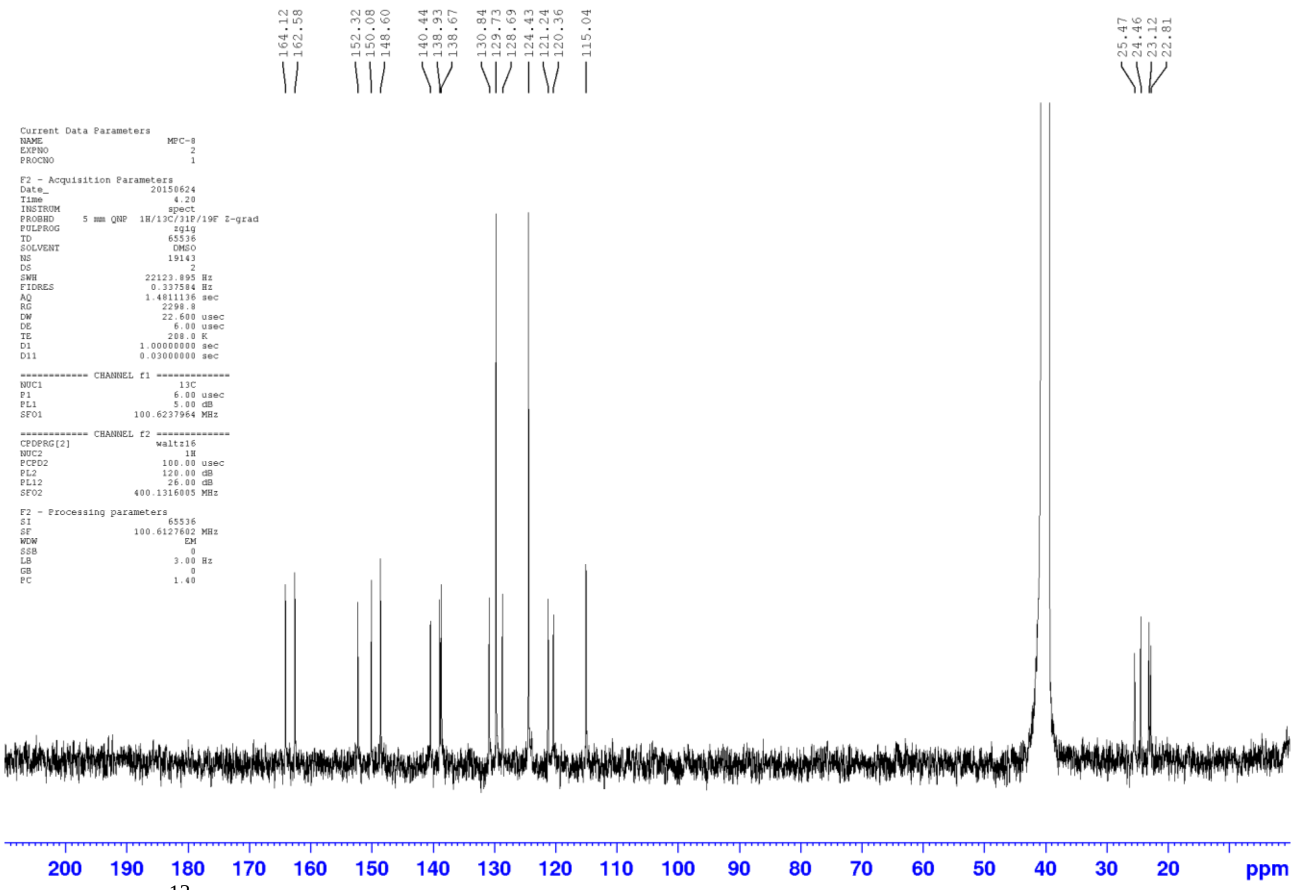

Figure S17. ^{13}C NMR spectrum of compound **2**.

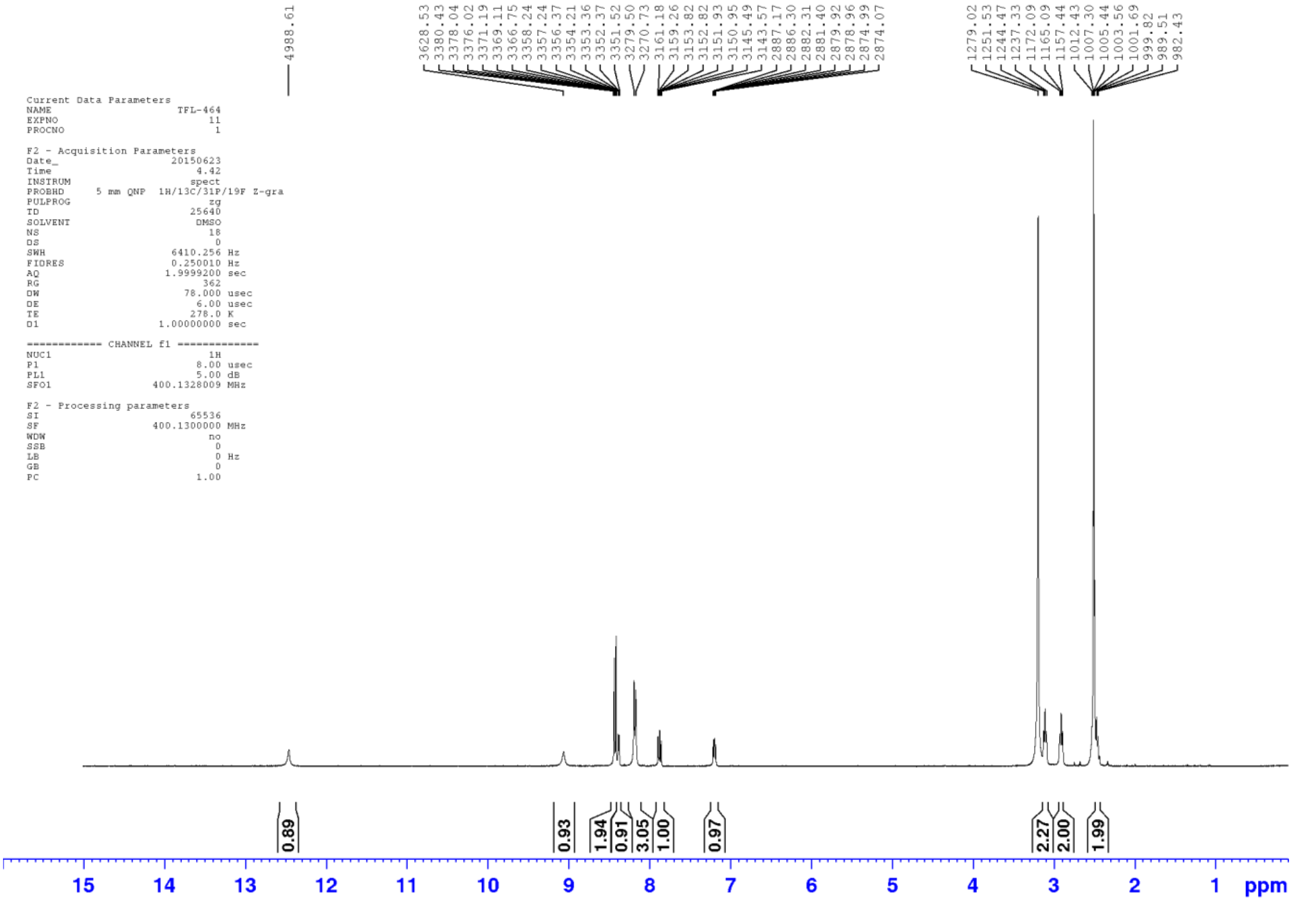

Figure S18. ^{1}H NMR spectrum of compound **3**.

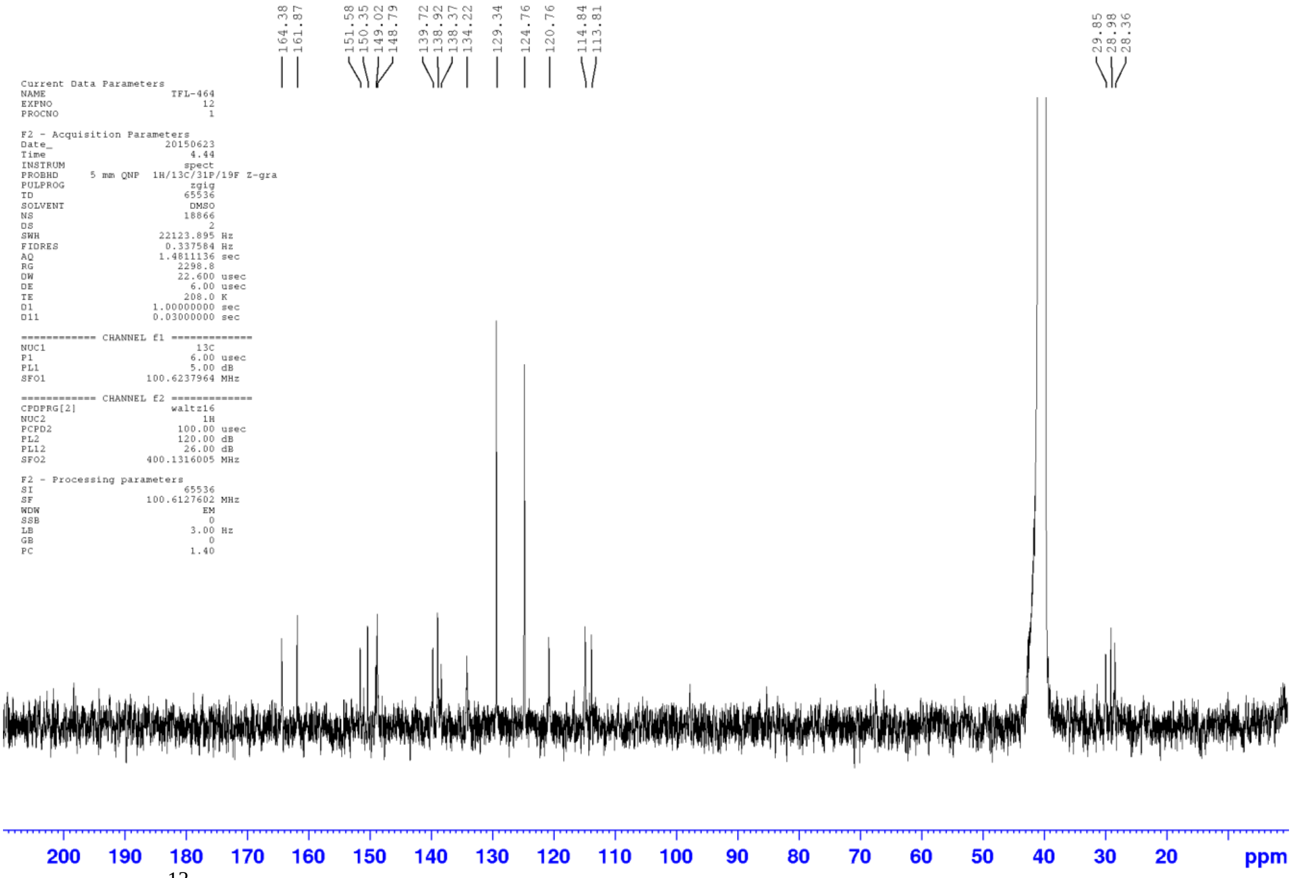

Figure S19. ^{13}C NMR spectrum of compound **3**.

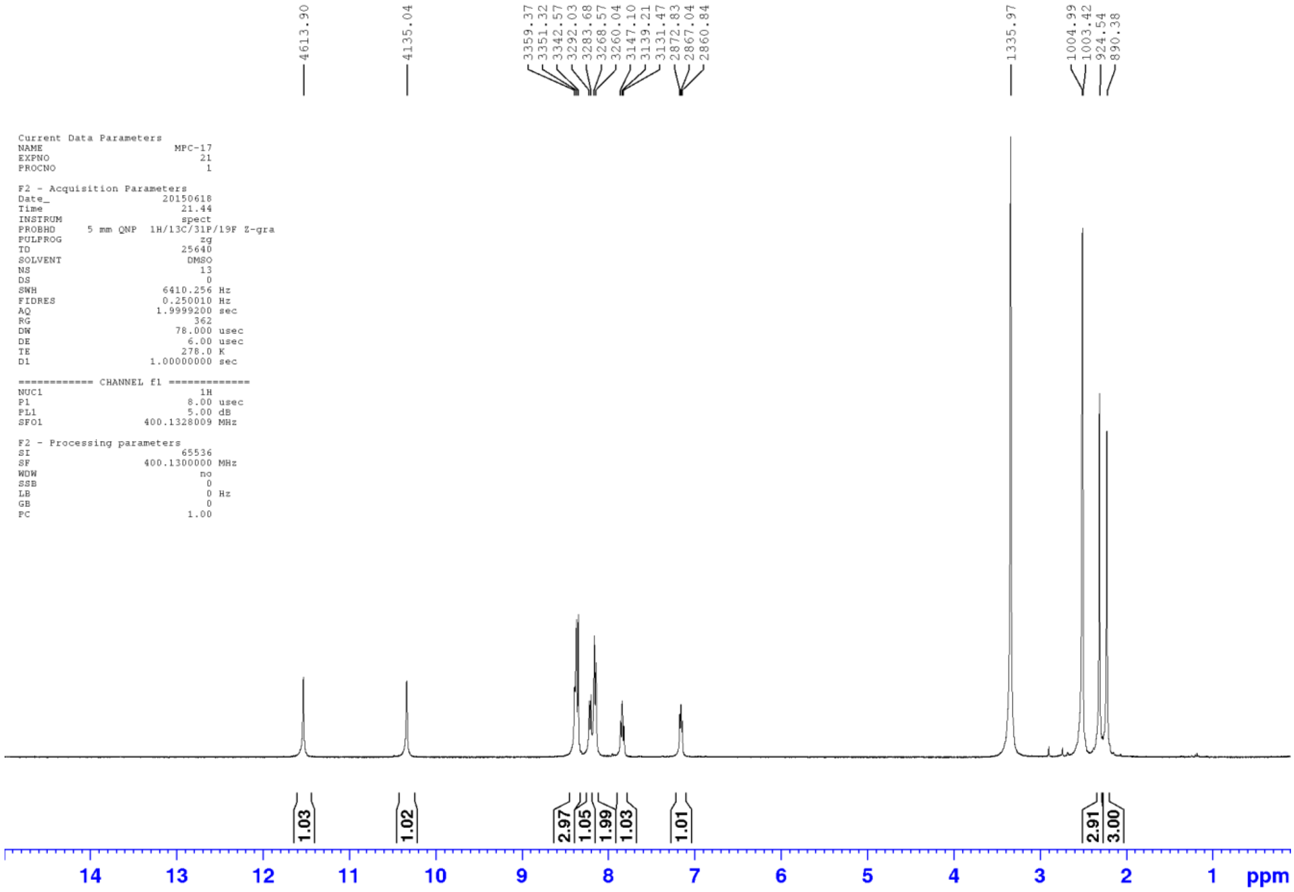

Figure S20. ^{1}H NMR spectrum of compound **4**.

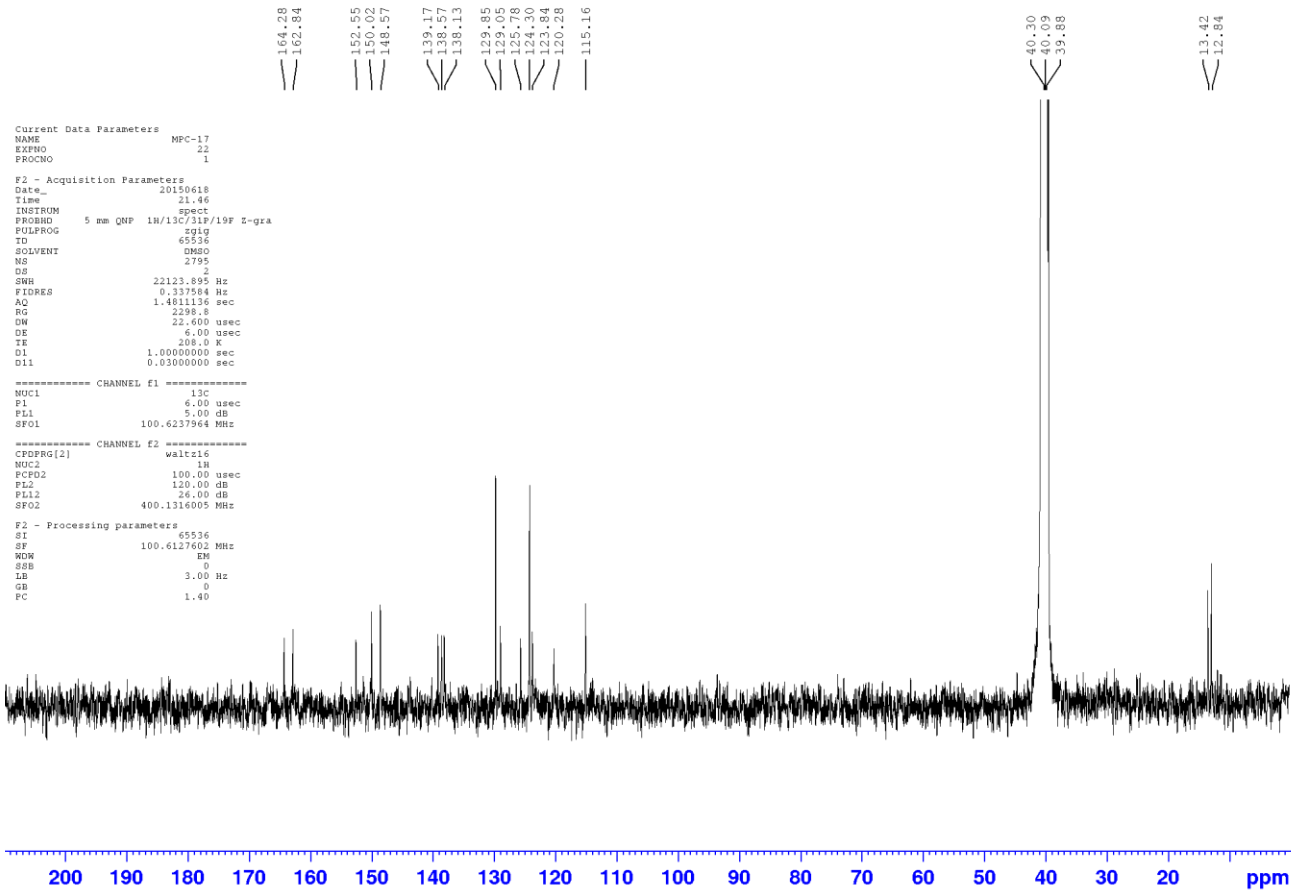

Figure S21. ^{13}C NMR spectrum of compound **4**.

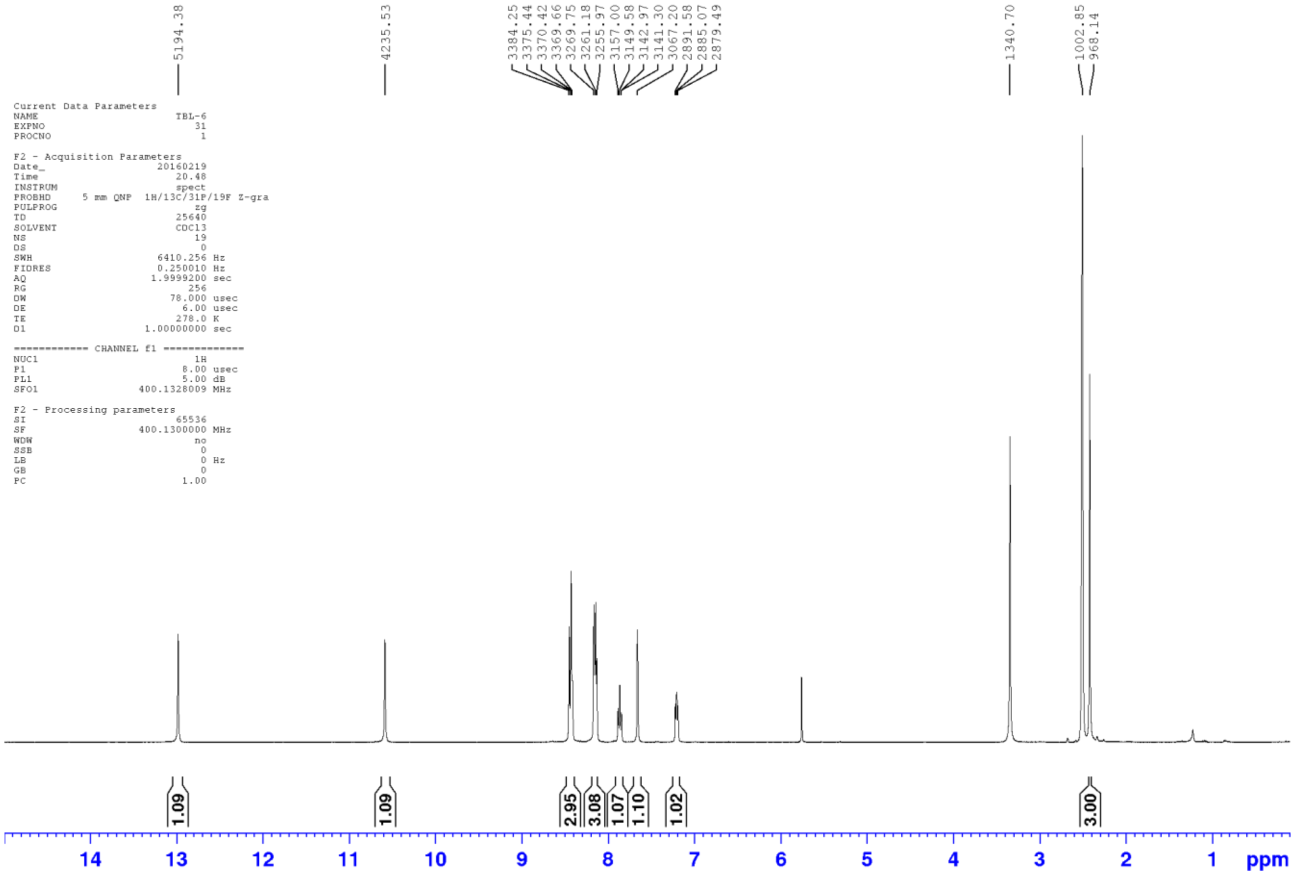

Figure S22. ^{1}H NMR spectrum of compound **5**.

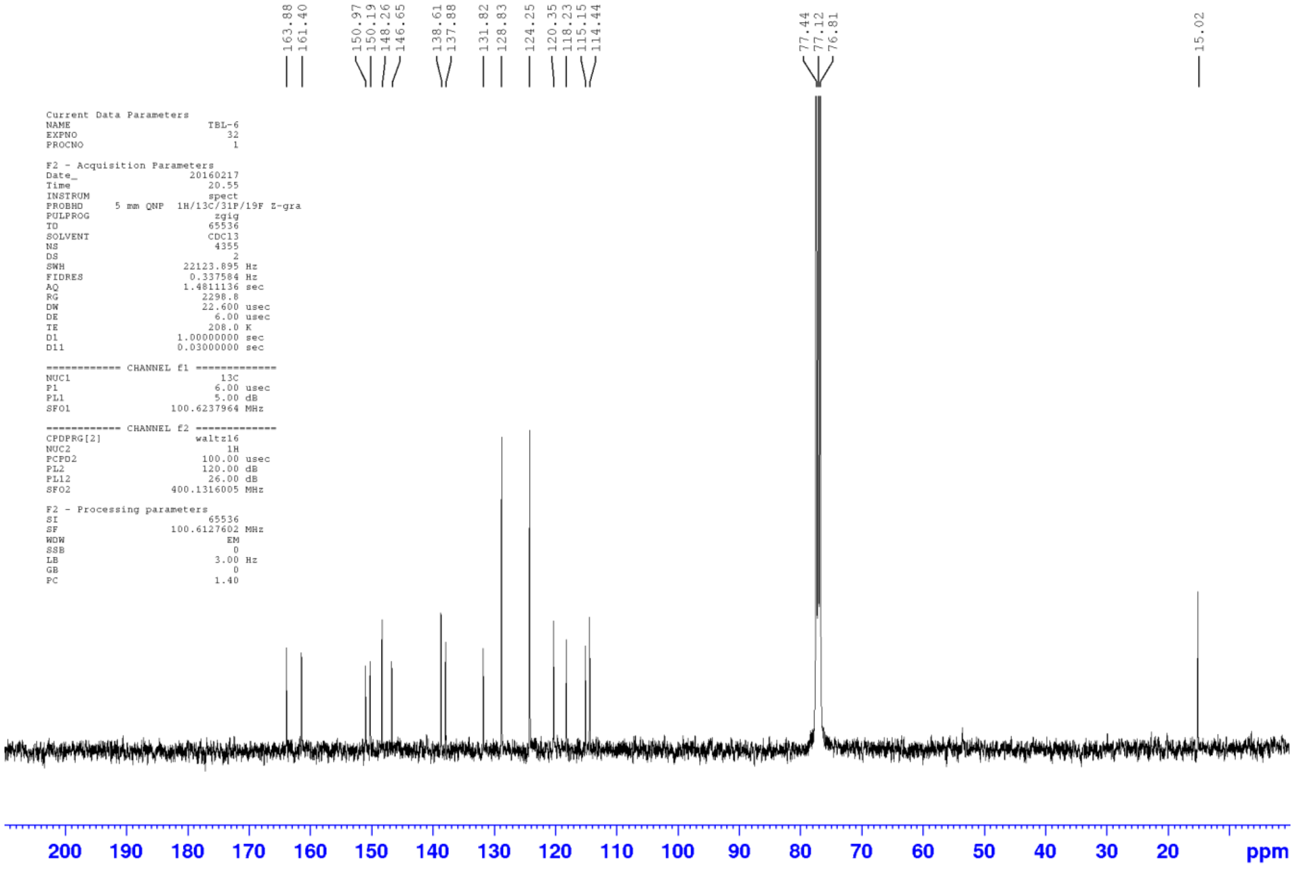

Figure S23. ^{13}C NMR spectrum of compound **5**.

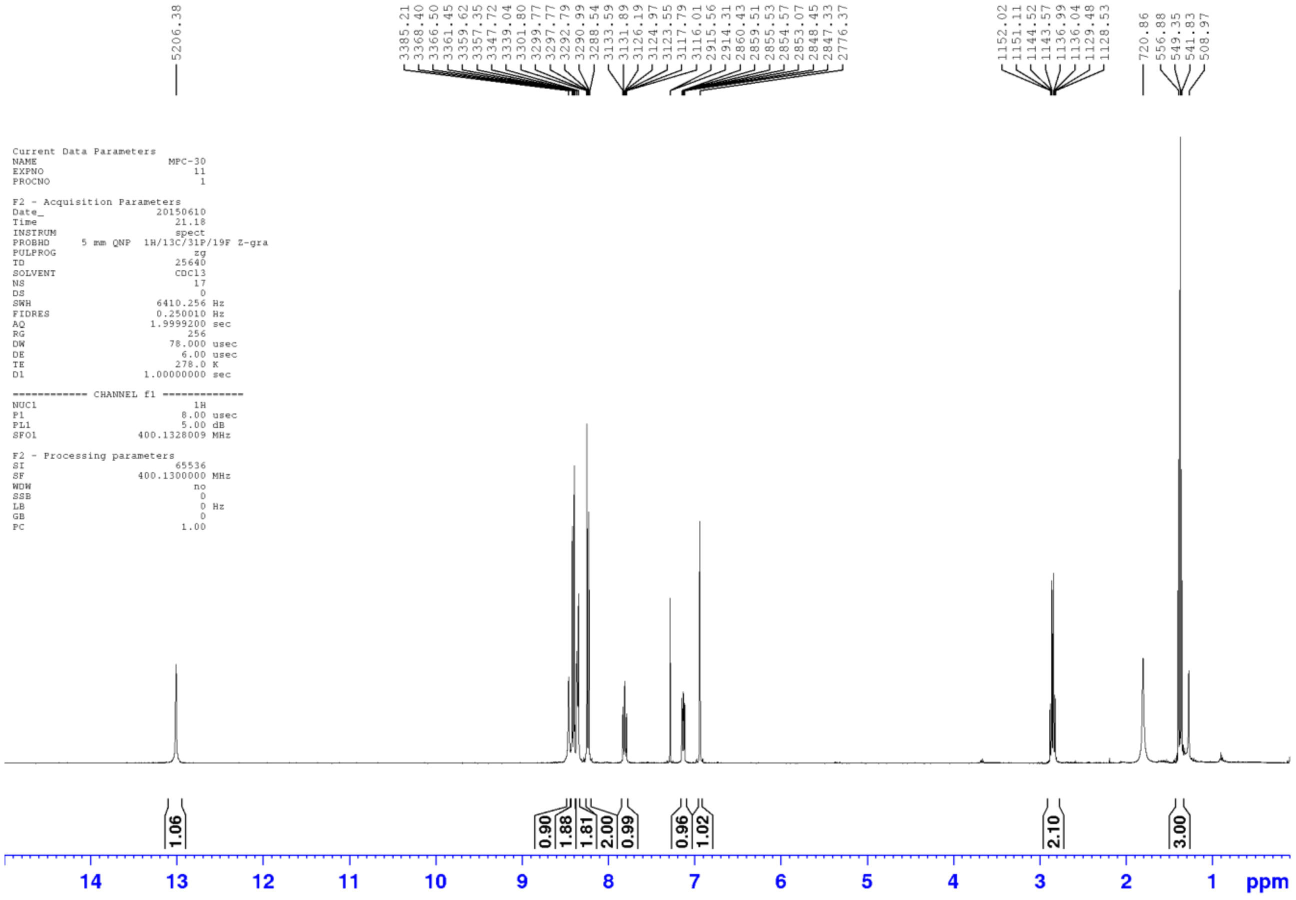

Figure S24. ^{1}H NMR spectrum of compound **6**.

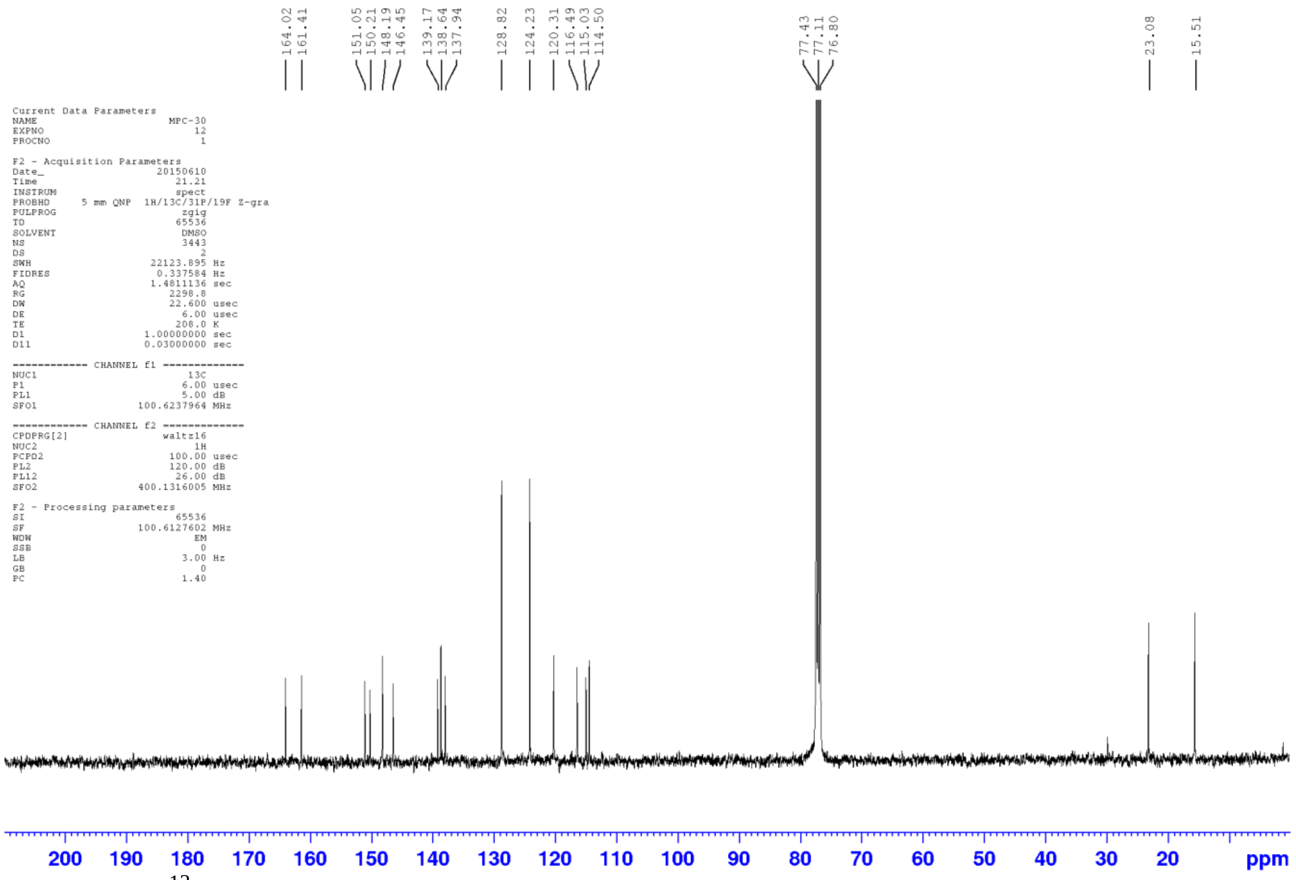

Figure S25. ^{13}C NMR spectrum of compound **6**.

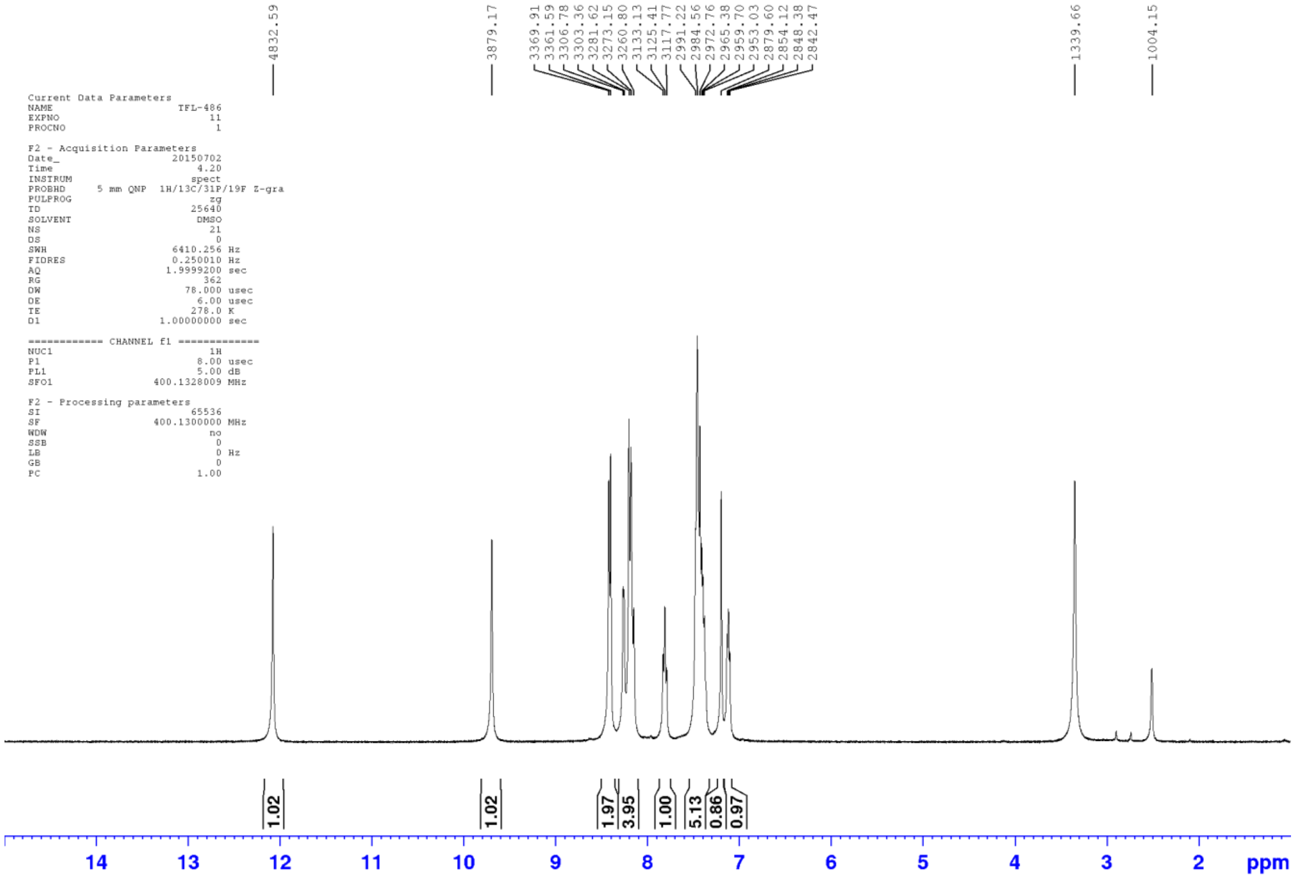

Figure S26. ^{1}H NMR spectrum of compound **7**.

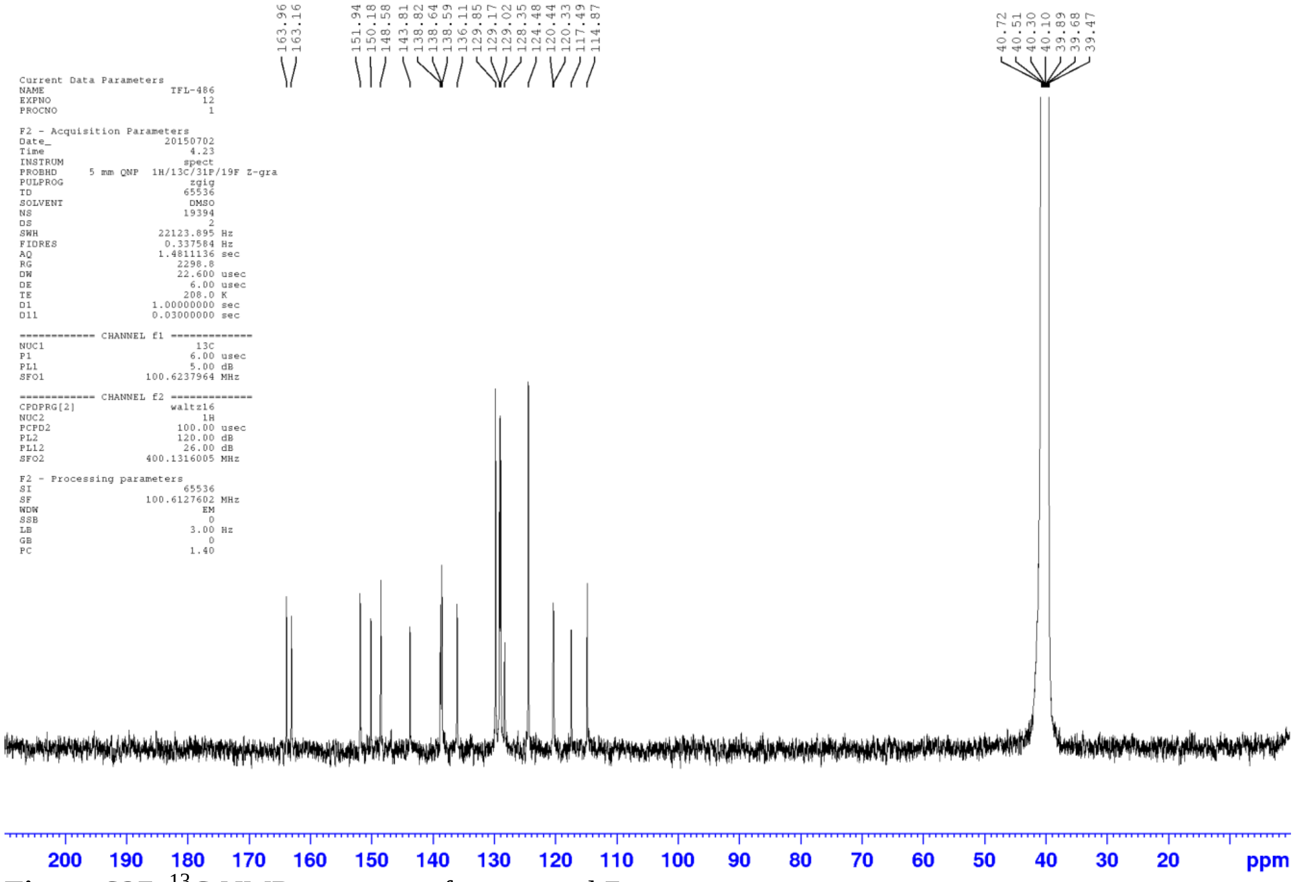

Figure S27. ^{13}C NMR spectrum of compound **7**.

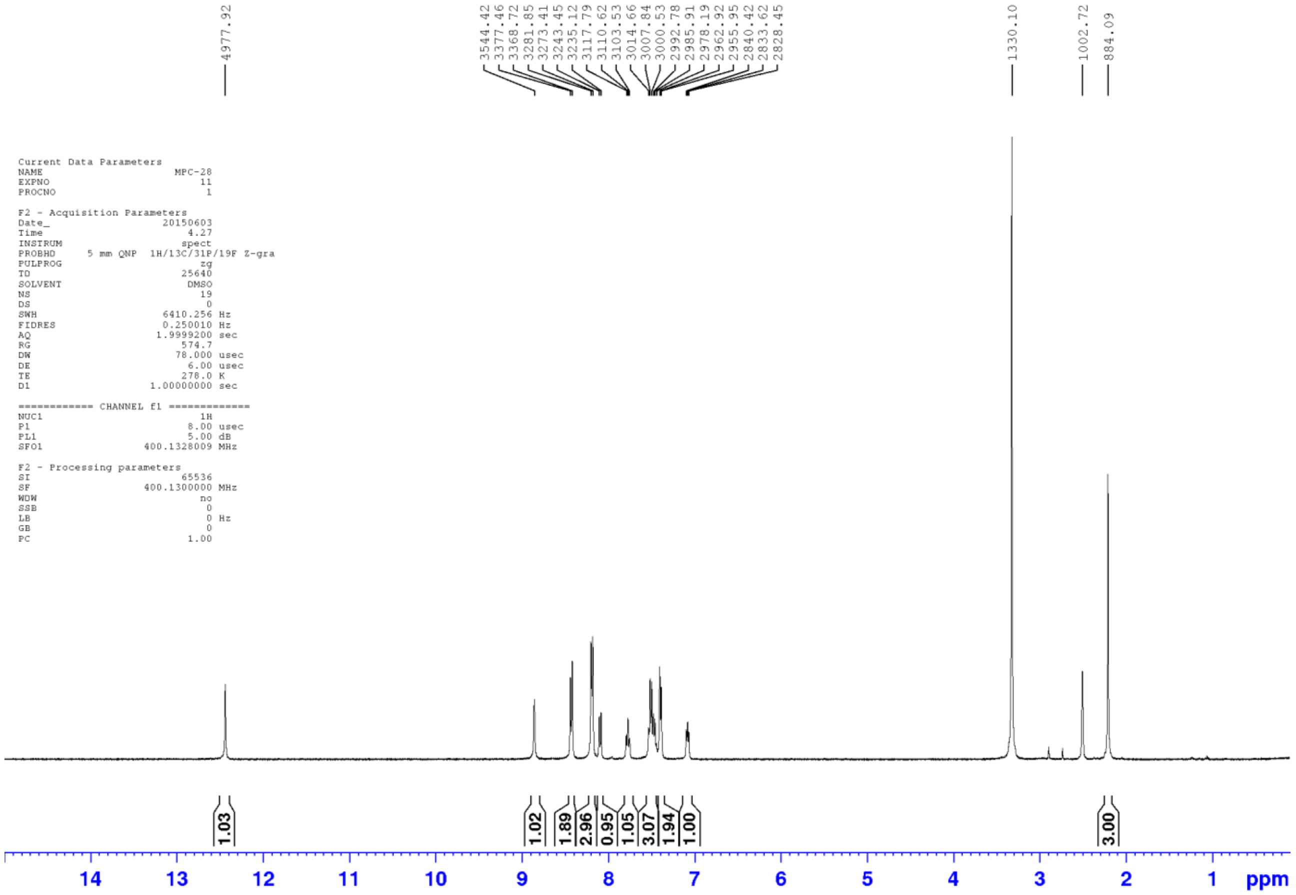

Figure S28. ^{1}H NMR spectrum of compound **8**.

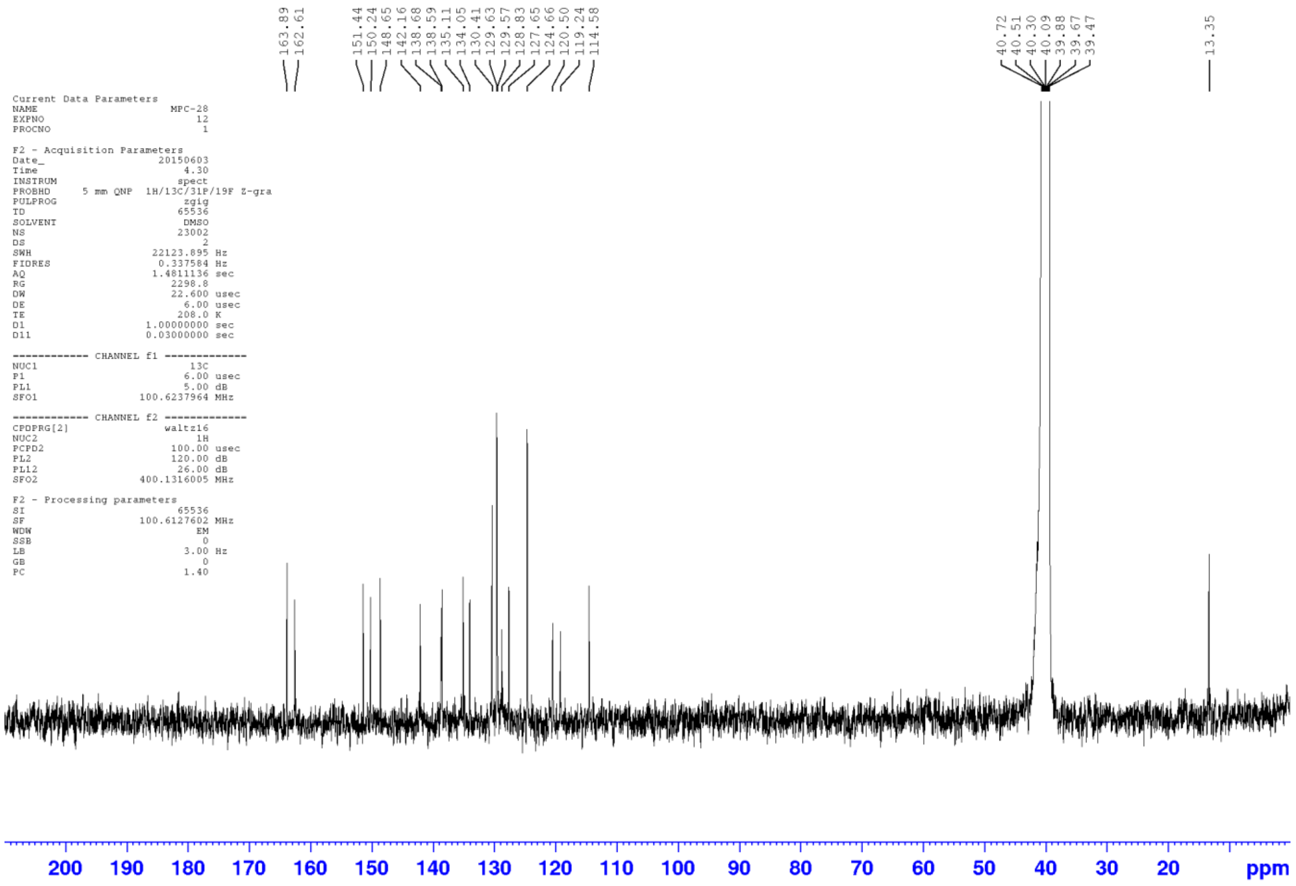

Figure S29. ^{13}C NMR spectrum of compound **8**.

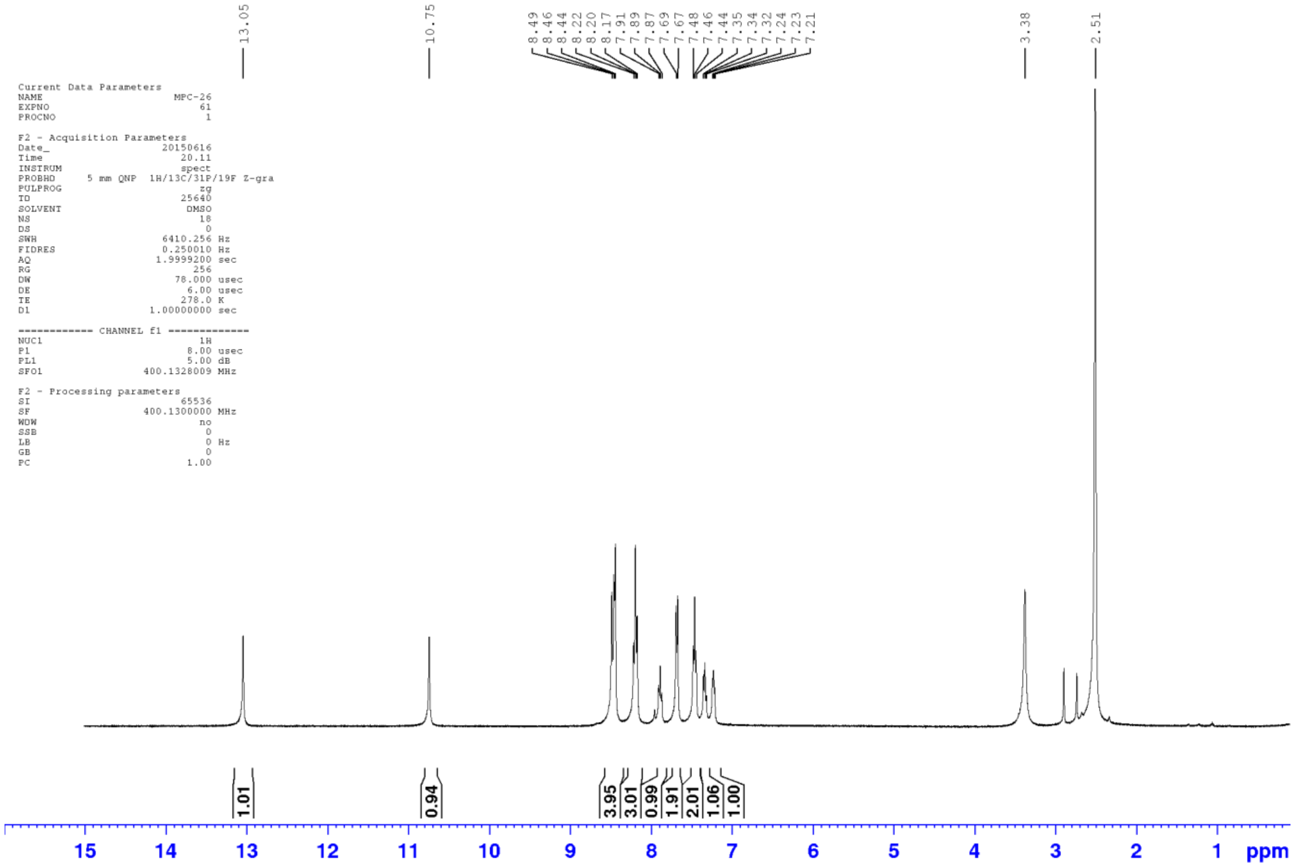

Figure S30. ^{1}H NMR spectrum of compound **9**.

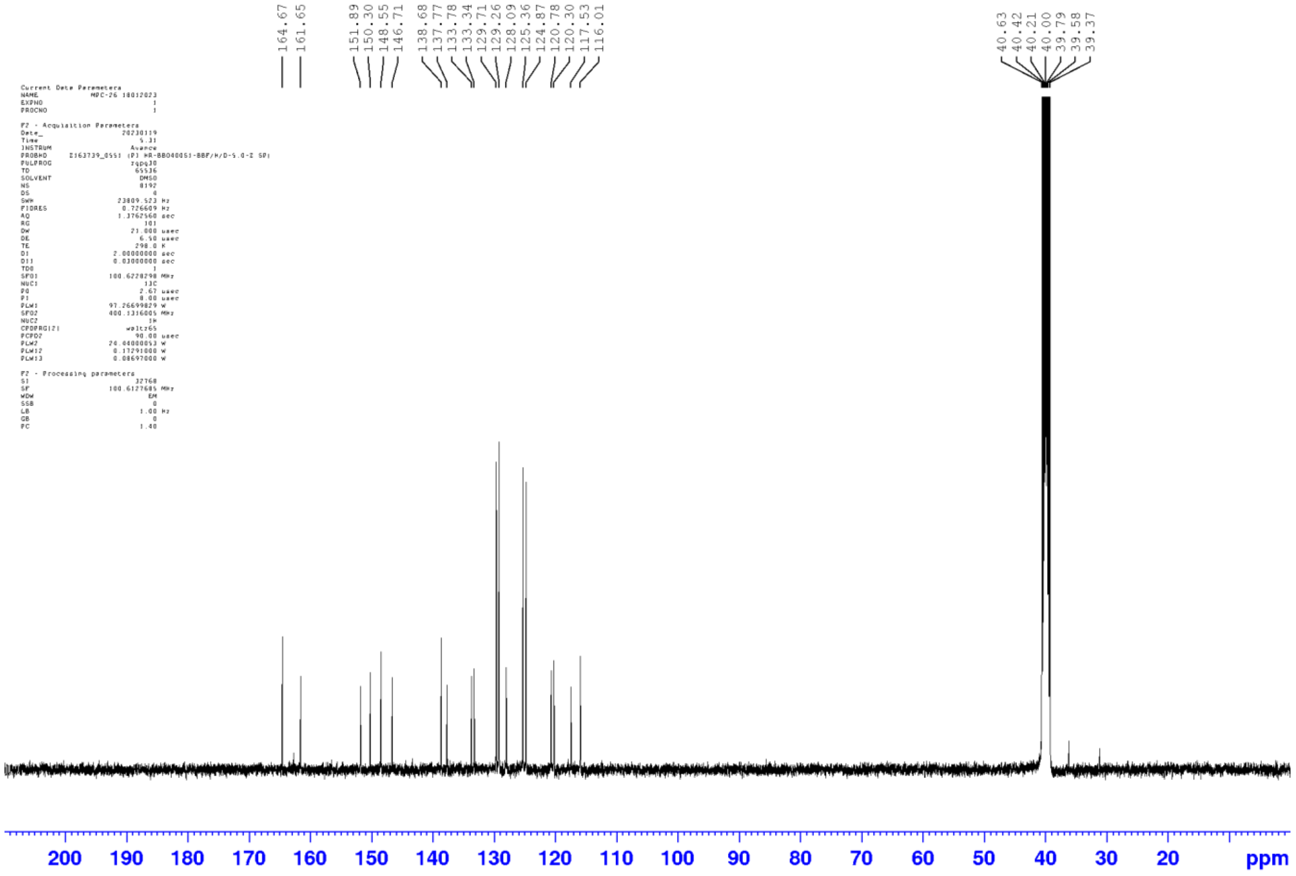

Figure S31. ^{13}C NMR spectrum of compound **9**.

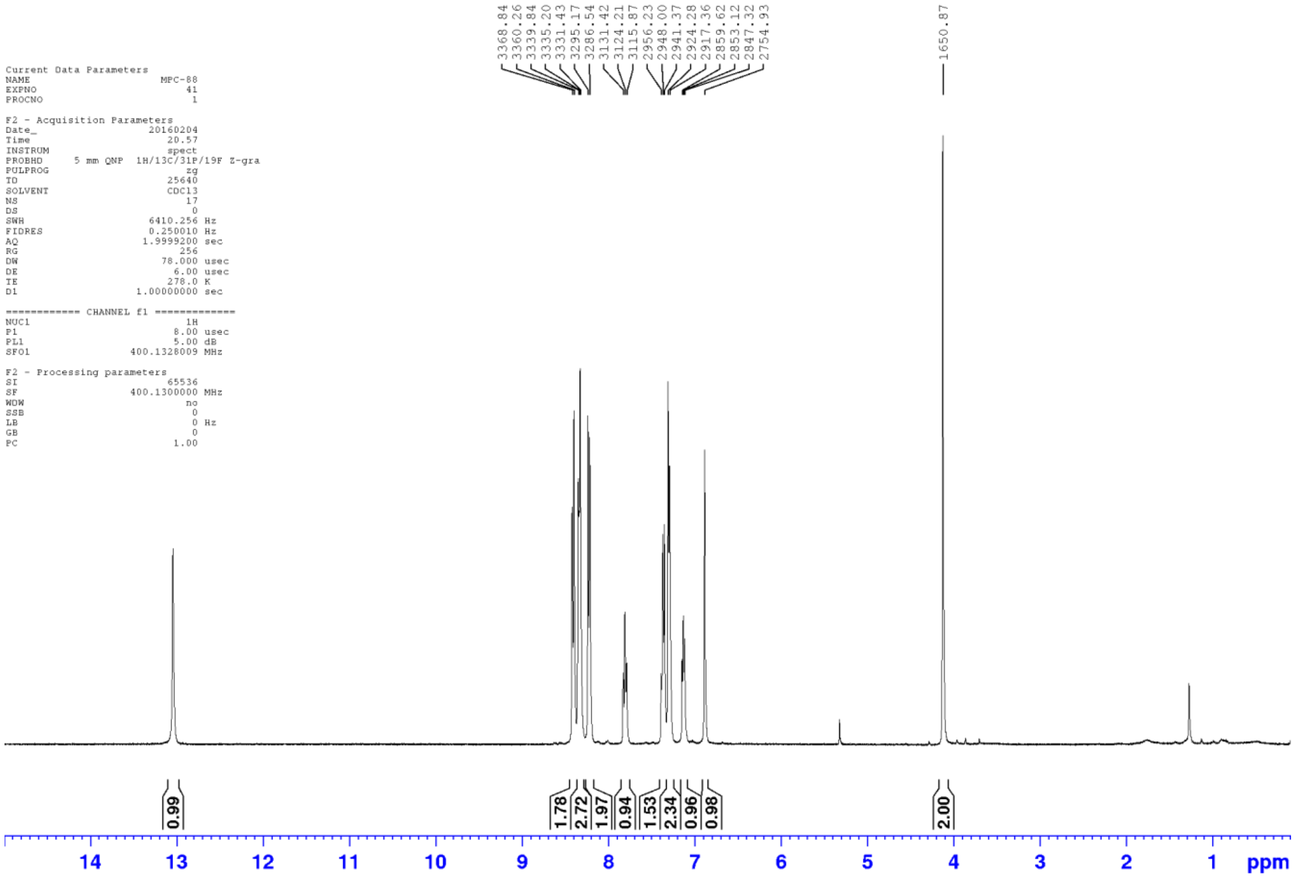

Figure S32. ^{1}H NMR spectrum of compound **10**.

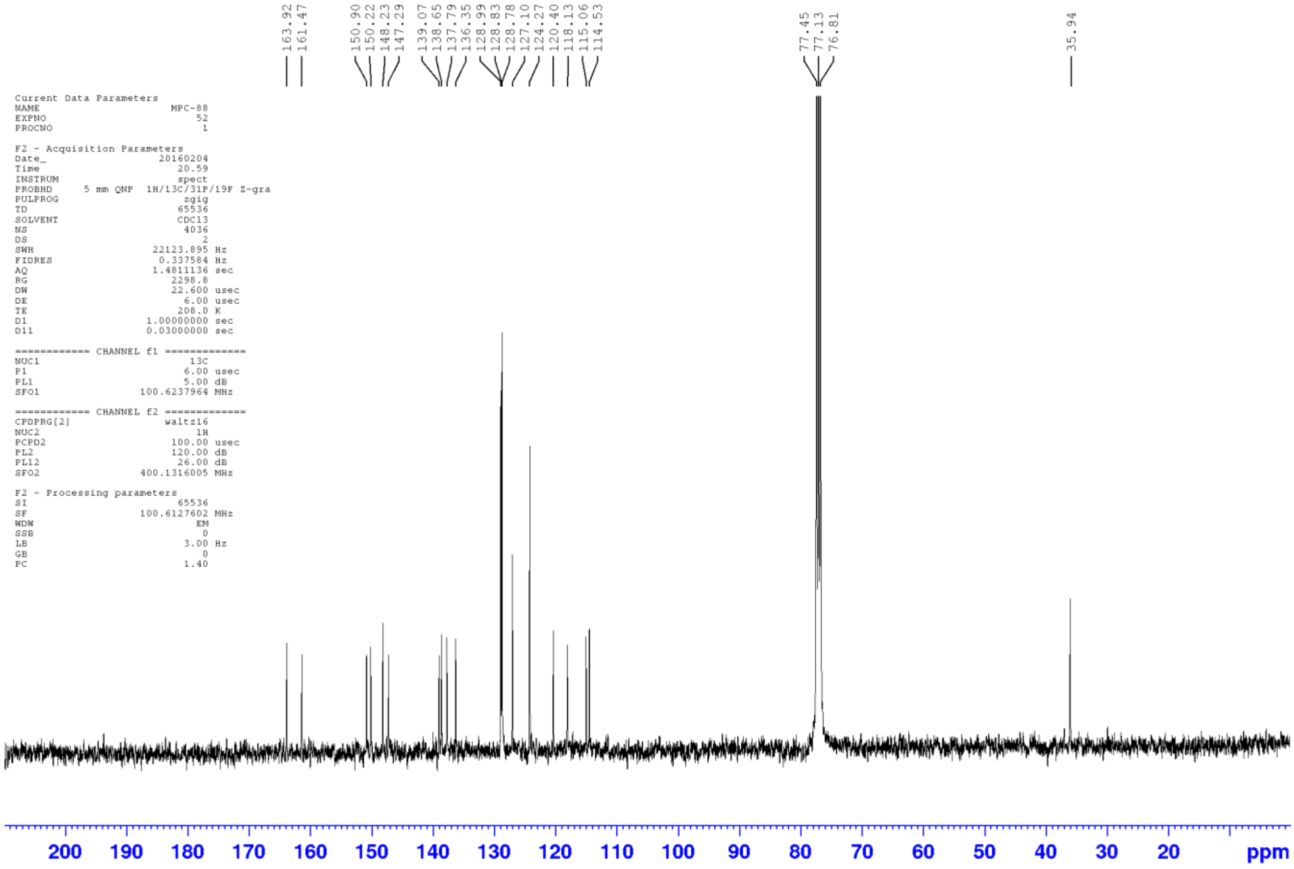

Figure S33. ^{13}C NMR spectrum of compound **10**.

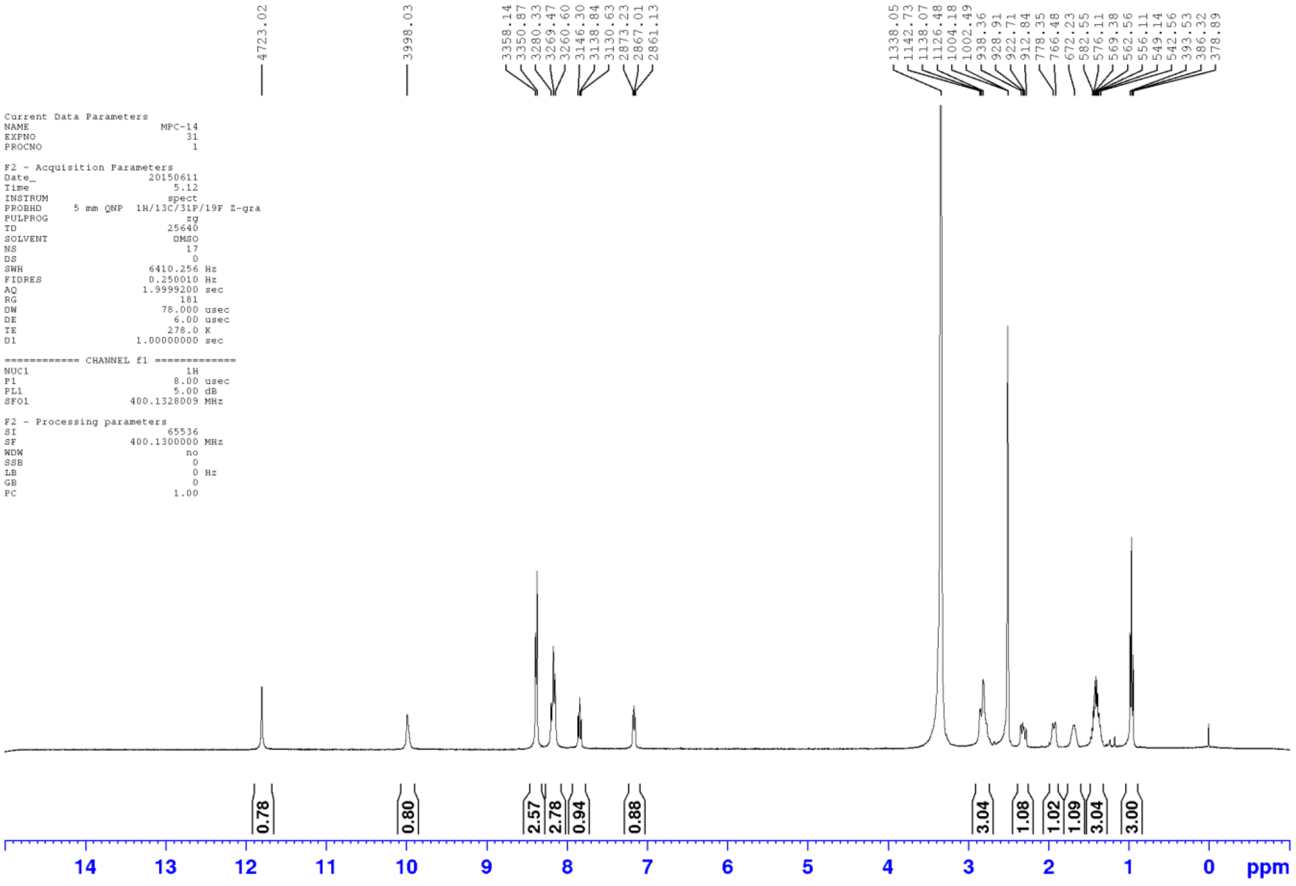

Figure S34. ^{1}H NMR spectrum of compound **11.**

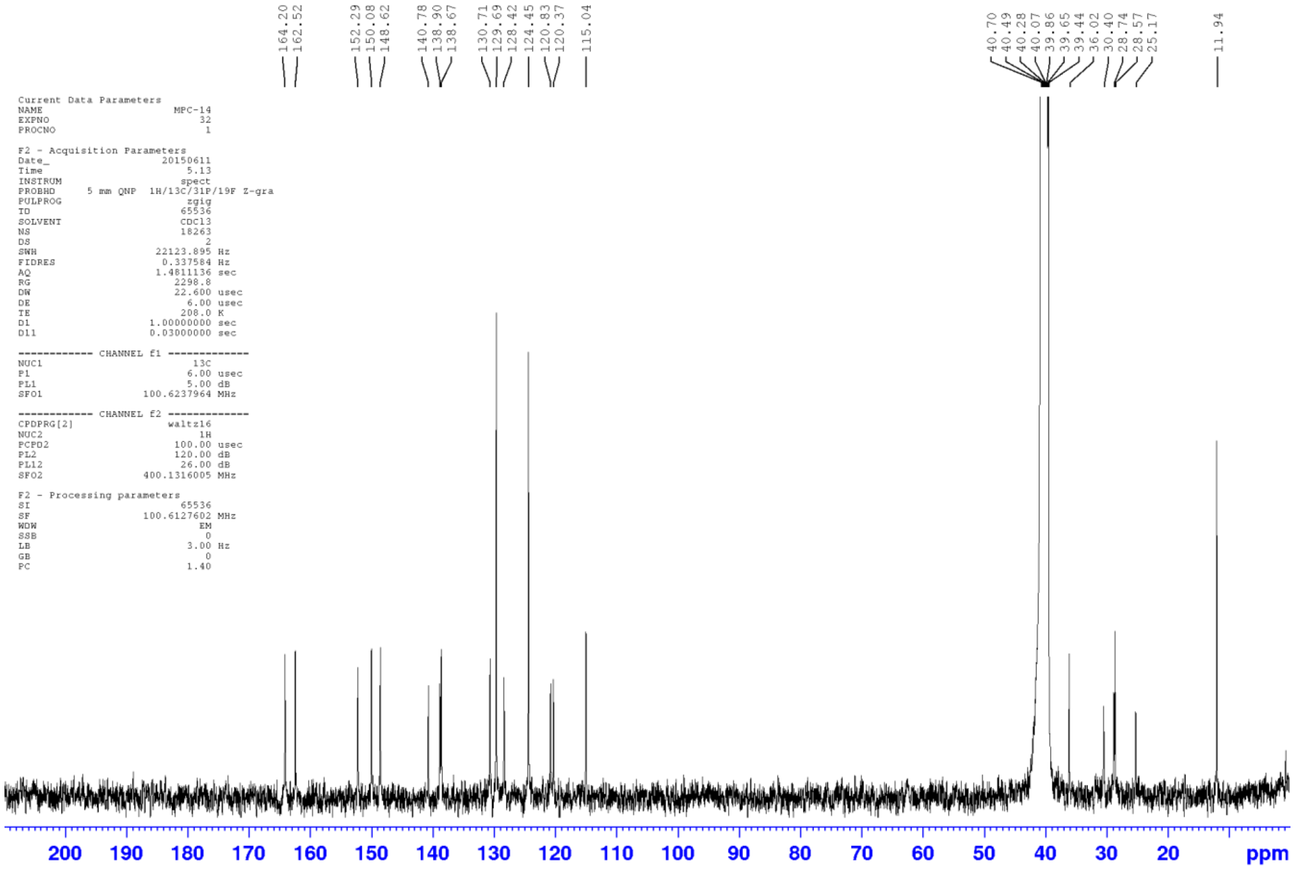

Figure S35. ^{13}C NMR spectrum of compound **11**.

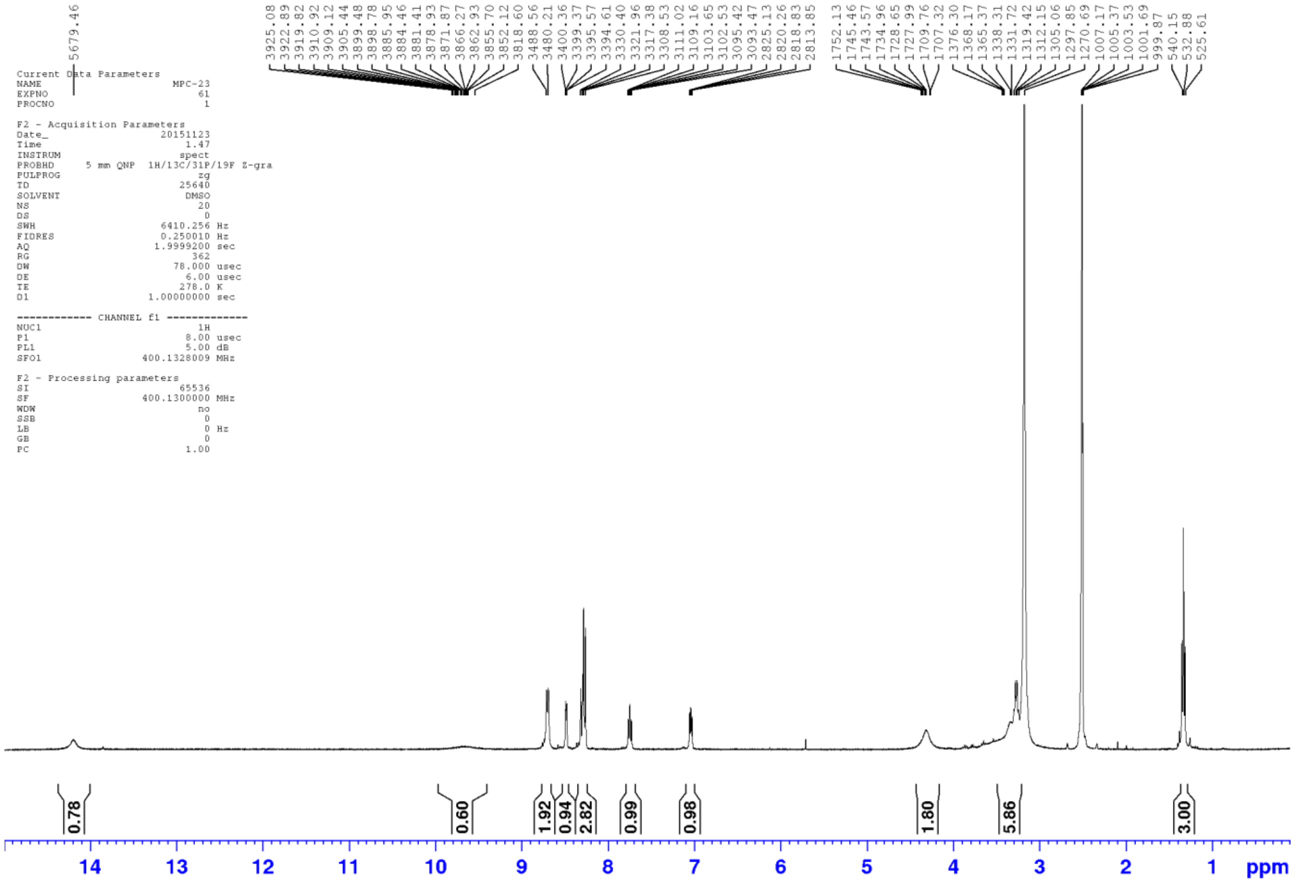

Figure S36. ^{1}H NMR spectrum of compound **12**.

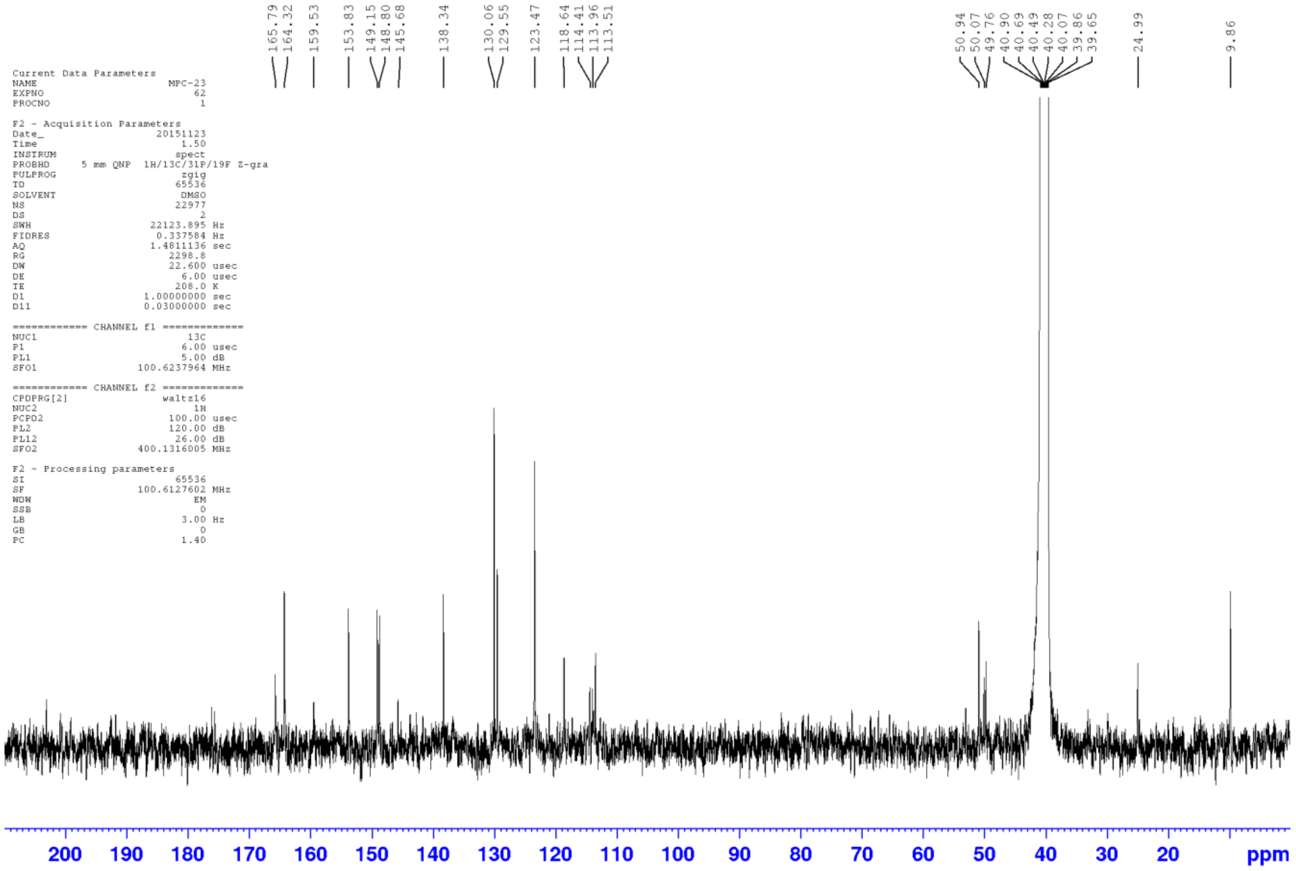

Figure S37. ^{13}C NMR spectrum of compound **12**.

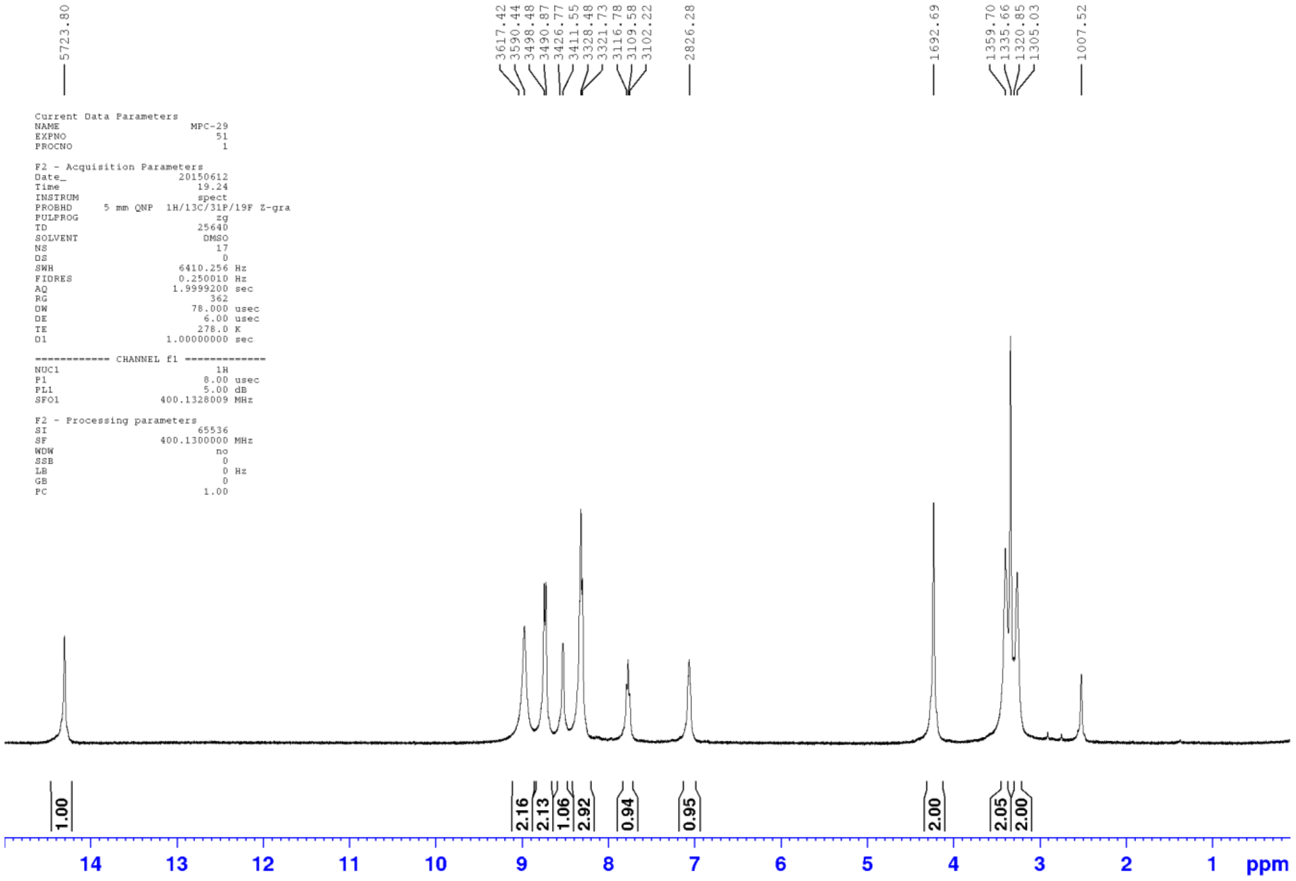

Figure S38. ^{1}H NMR spectrum of compound **13**.

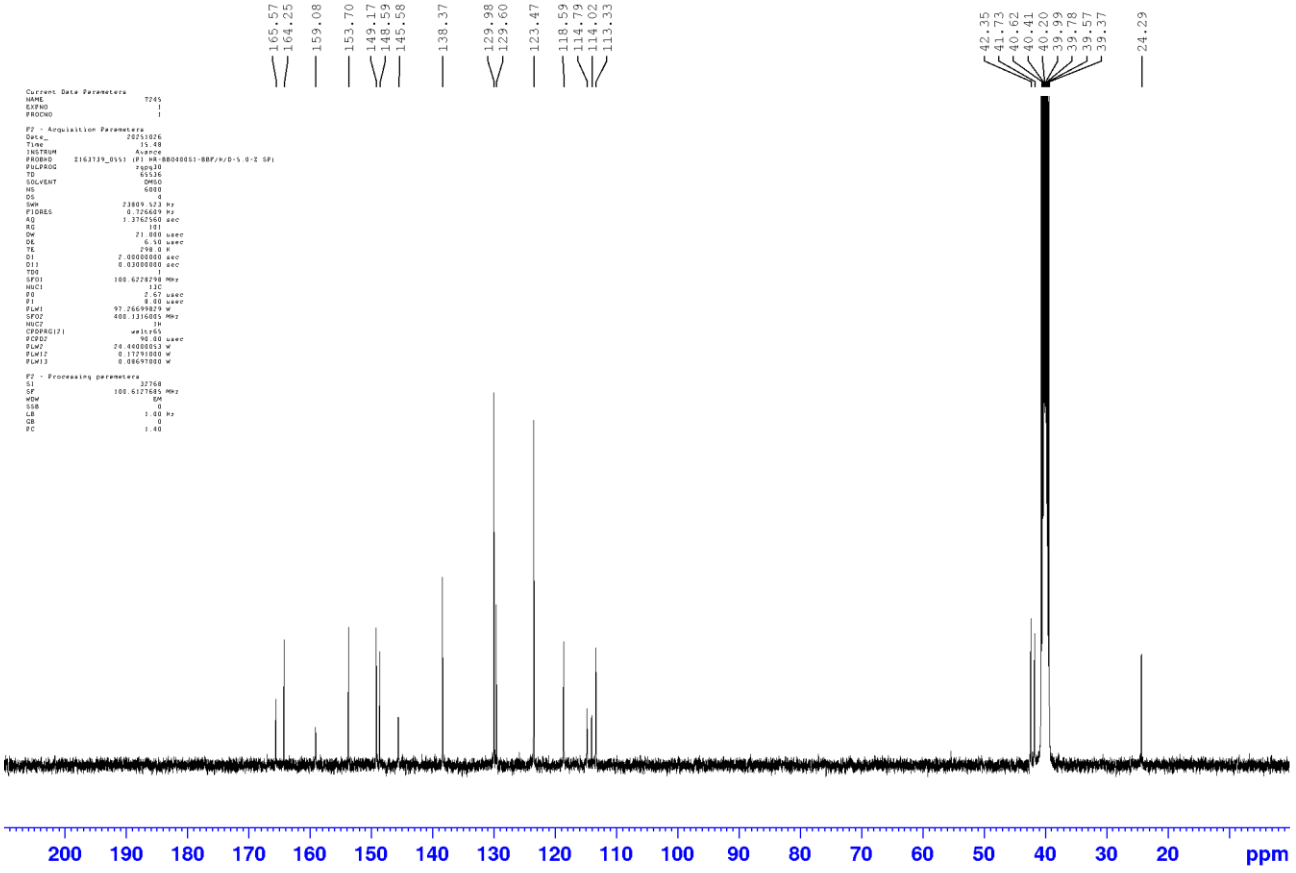

Figure S39. ^{13}C NMR spectrum of compound **13**.

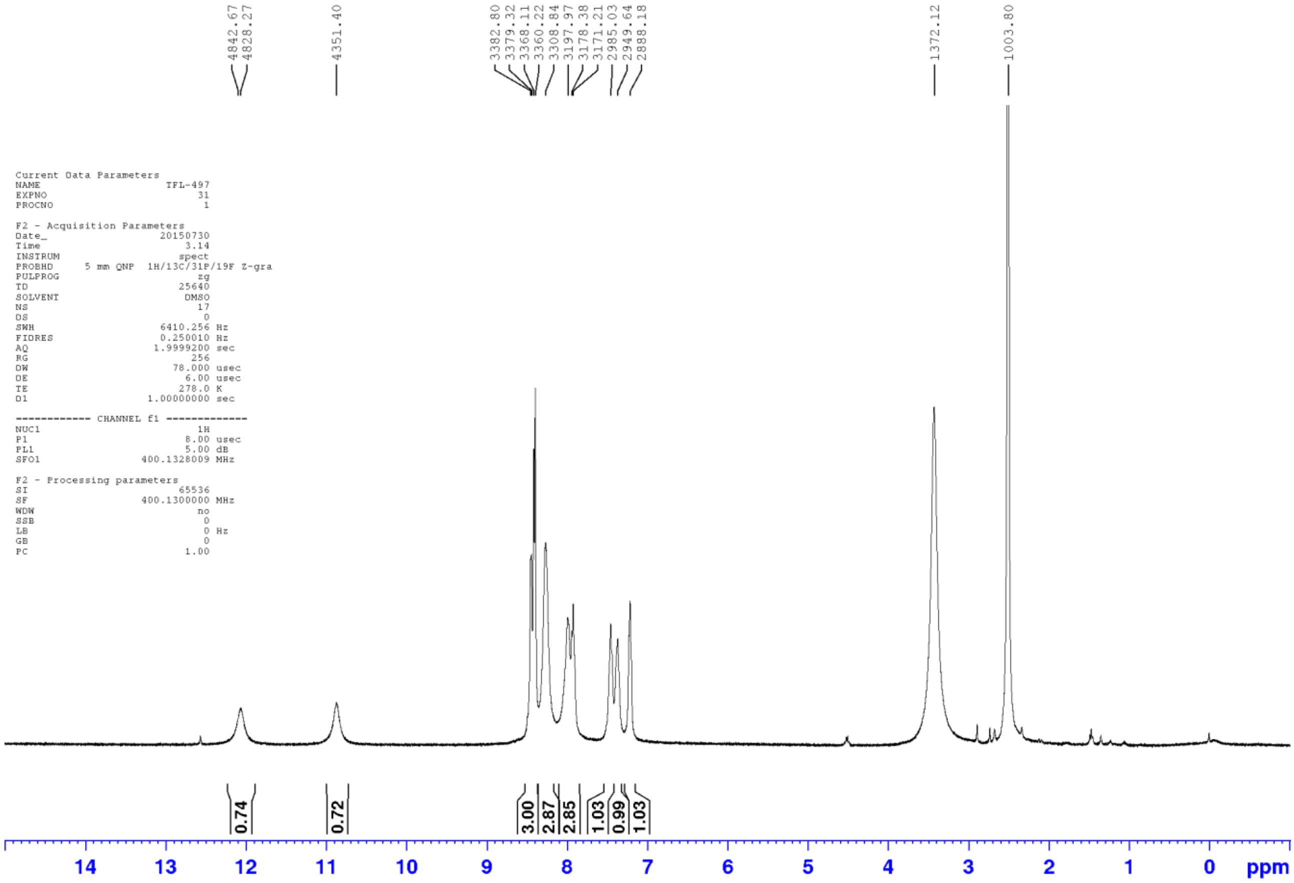

Figure S40. ^{1}H NMR spectrum of compound **14**.

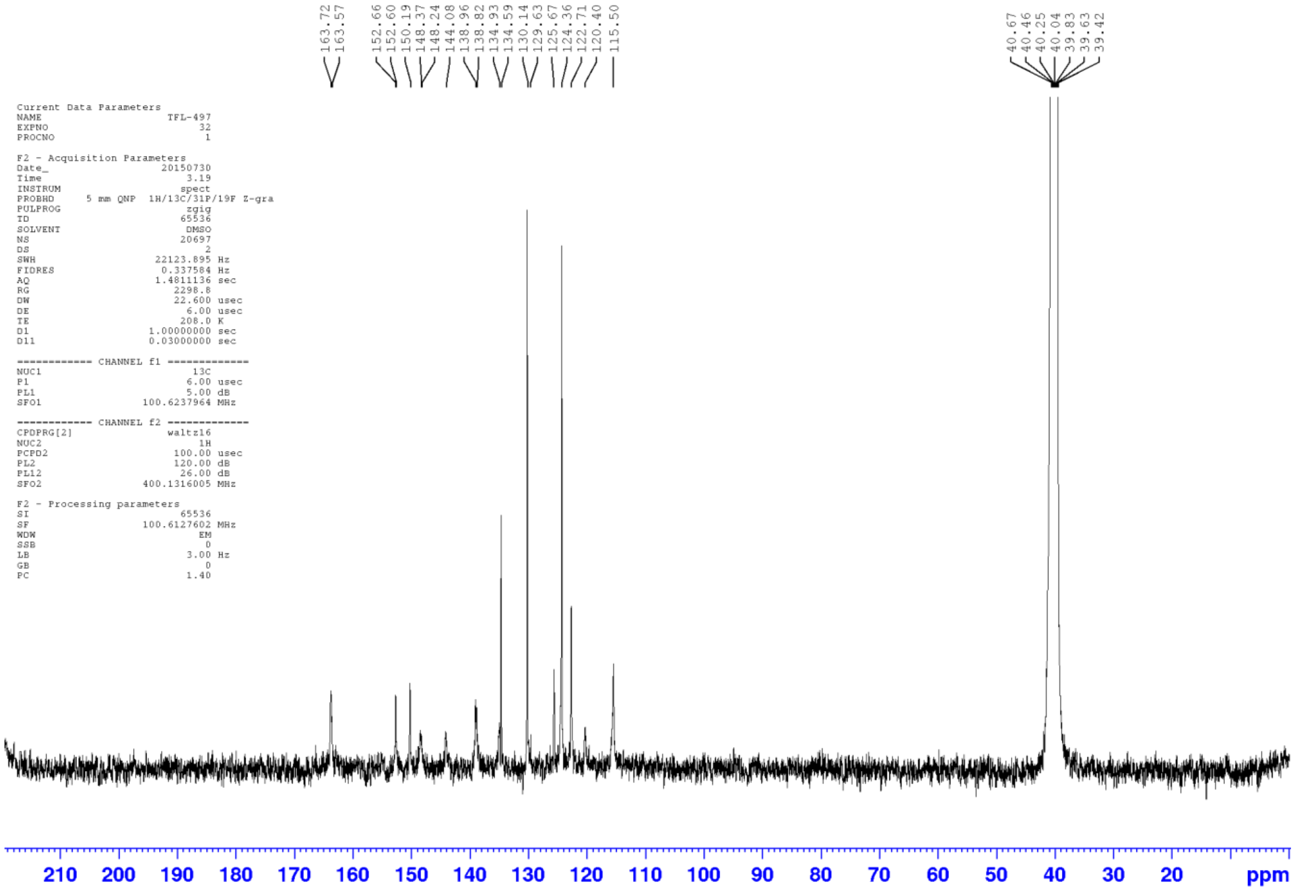

Figure S41. ^{13}C NMR spectrum of compound **14**.

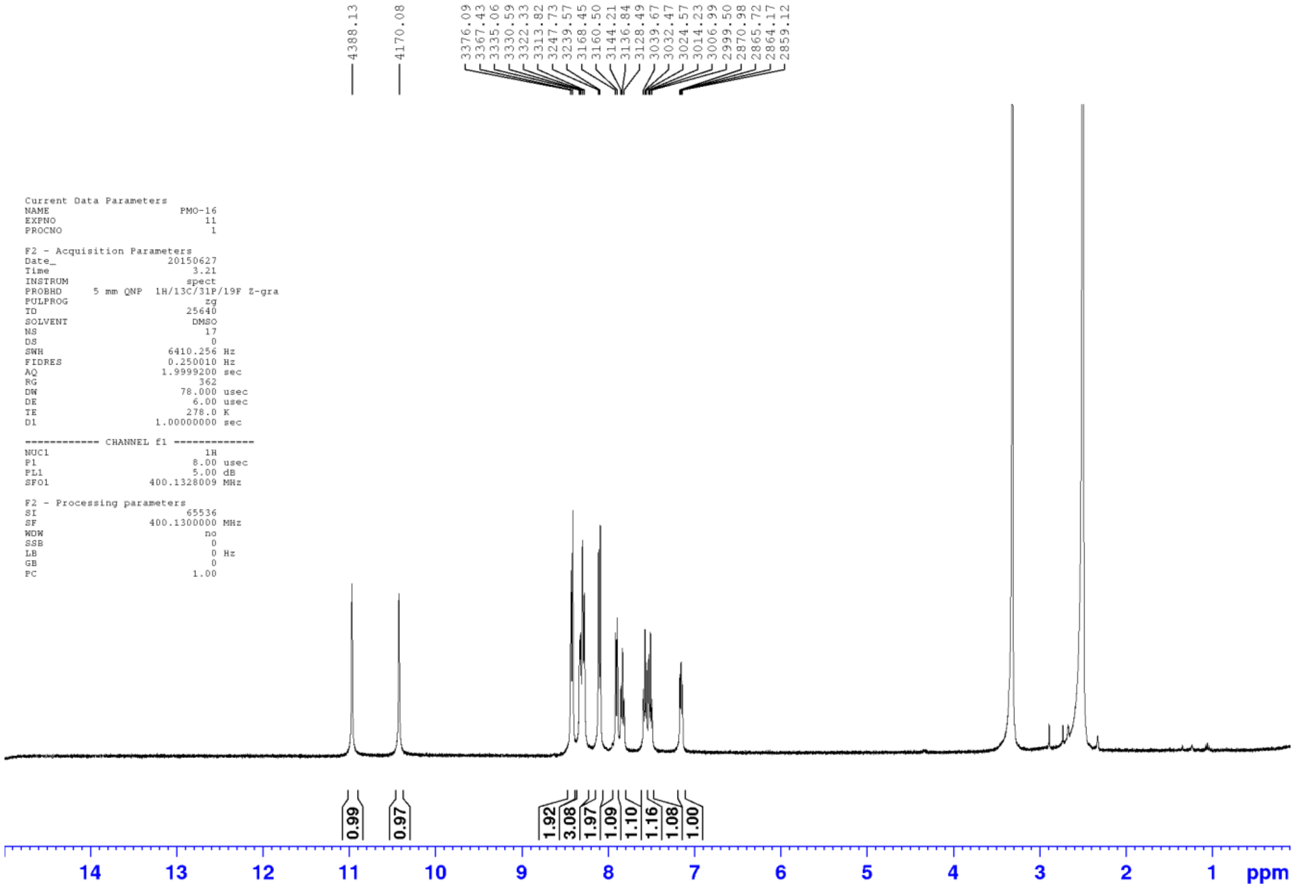

Figure S42. ^{1}H NMR spectrum of compound **15**.

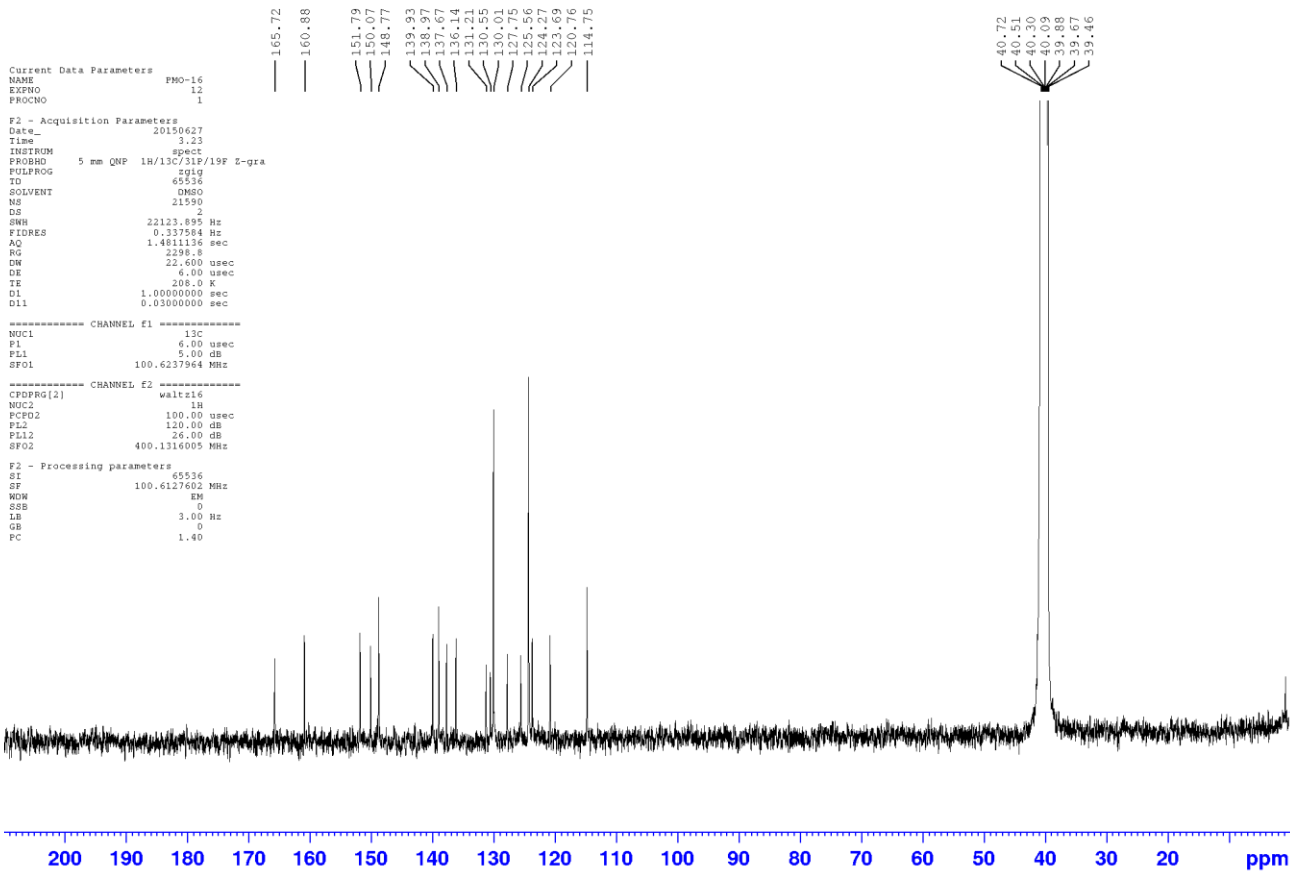

Figure S43. ^{13}C NMR spectrum of compound **15**.

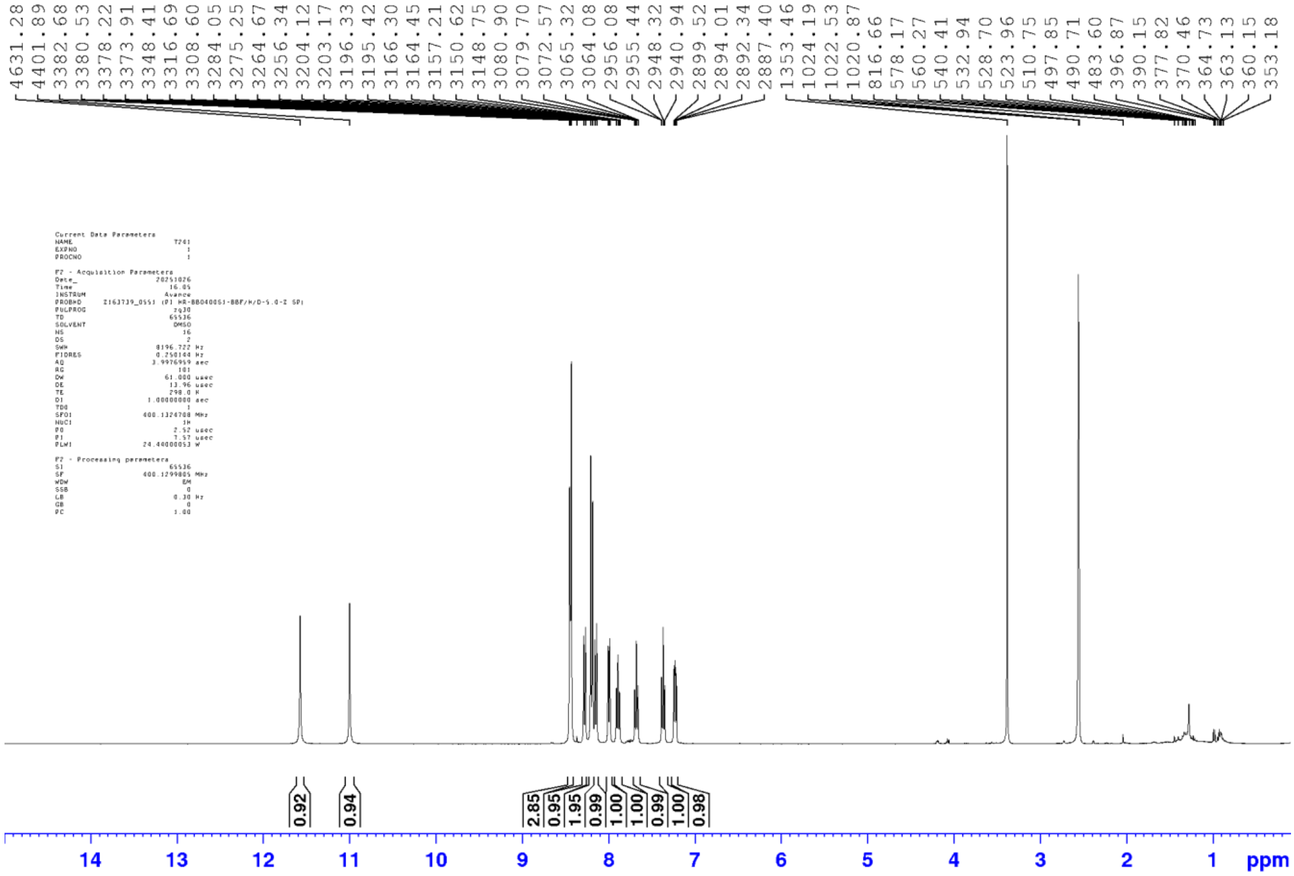

Figure S44. ^{1}H NMR spectrum of compound **16**.

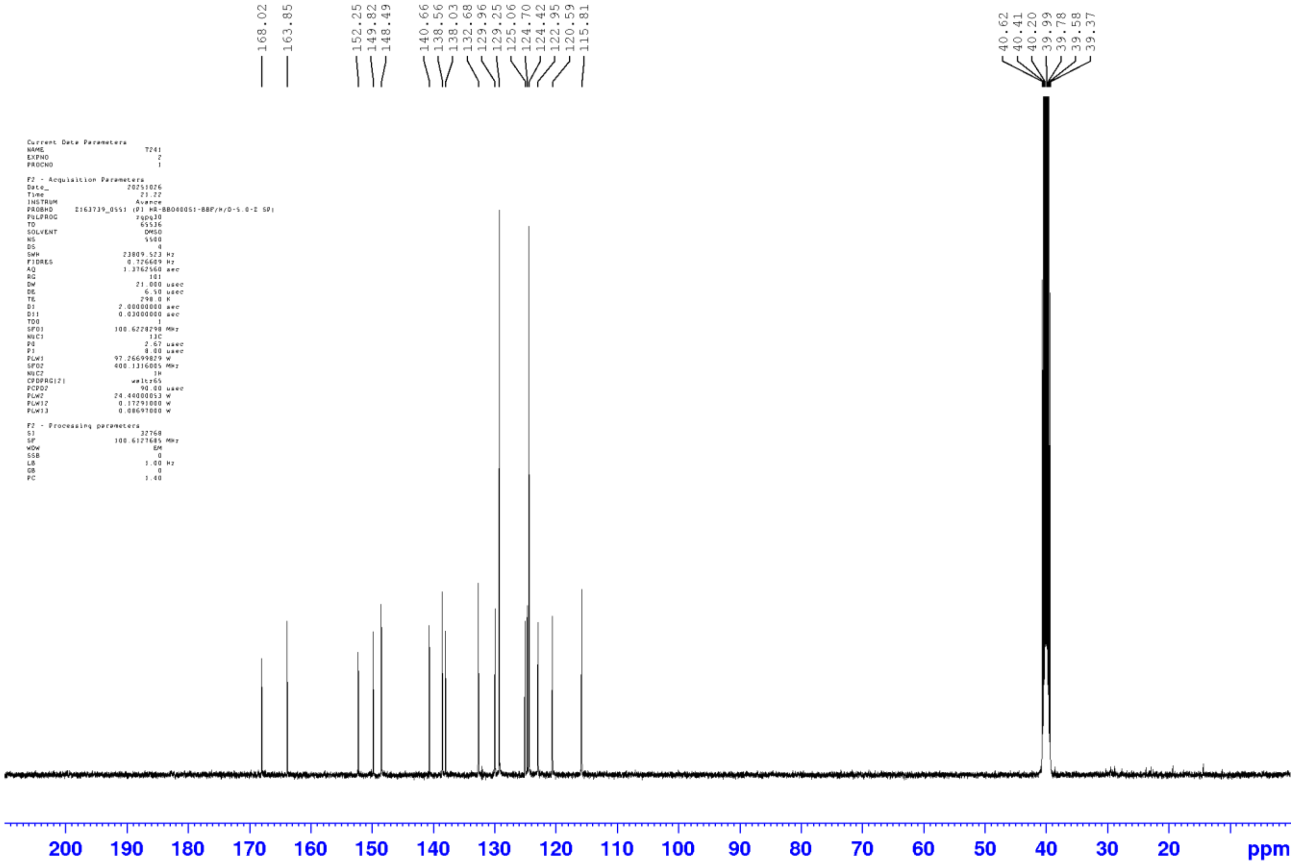

Figure S44. ^{13}C NMR spectrum of compound **16**.

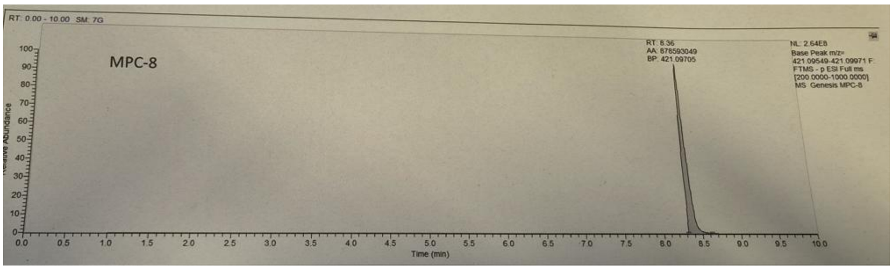

Figure S45. HRMS spectrum of compound **2**.

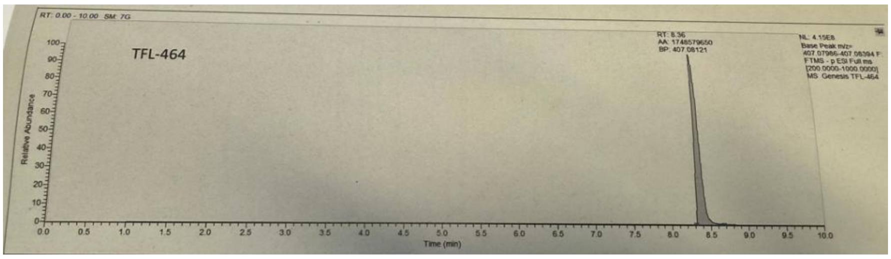

Figure S46. HRMS spectrum of compound **3**.

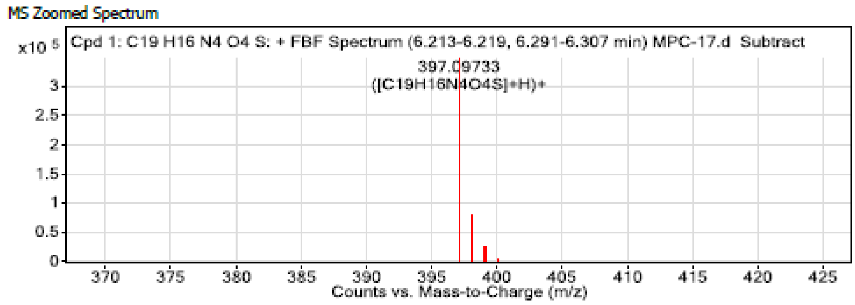

Figure S47. HRMS spectrum of compound **4**.

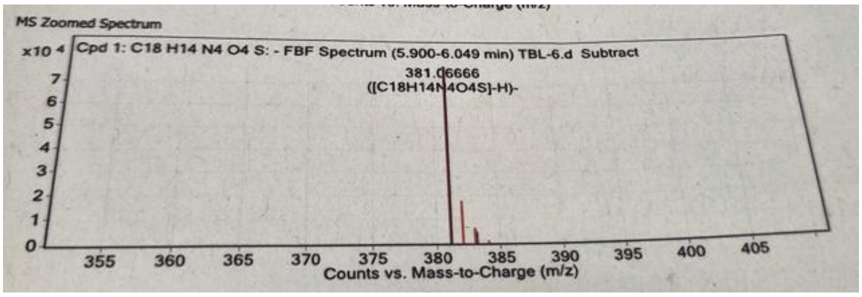

Figure S48. HRMS spectrum of compound **5**.

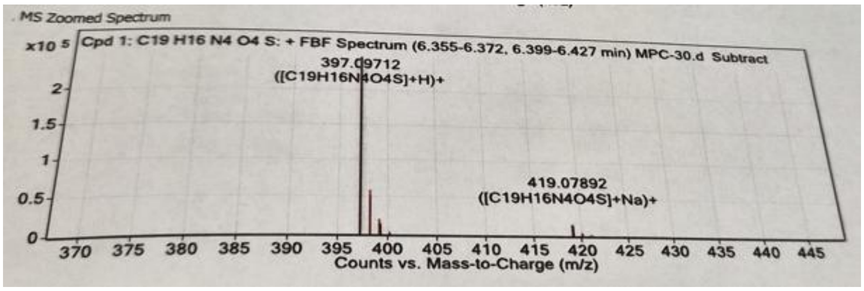

Figure S49. HRMS spectrum of compound **6**.

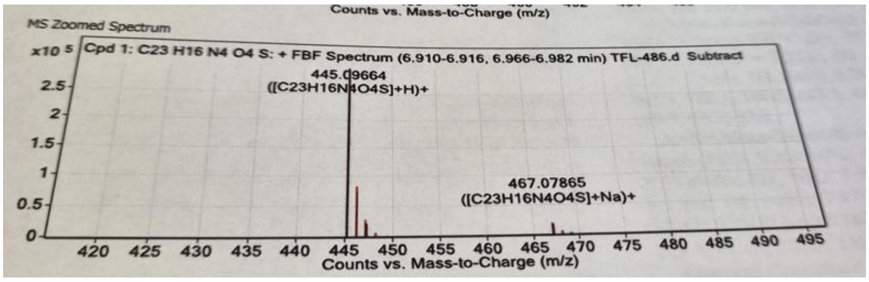

Figure S50. HRMS spectrum of compound **7**.

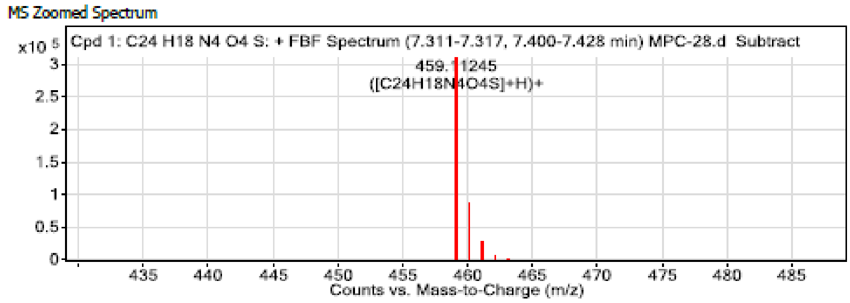

Figure S51. HRMS spectrum of compound **8**.

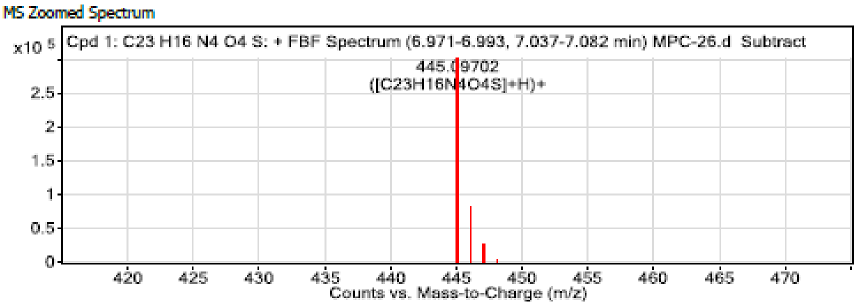

Figure S52. HRMS spectrum of compound **9**.

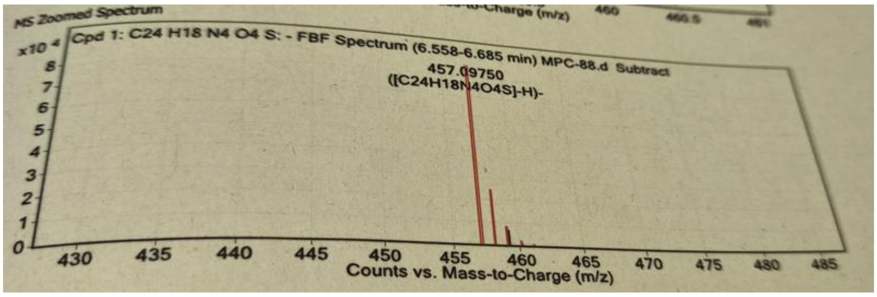

Figure S53. HRMS spectrum of compound **10**.

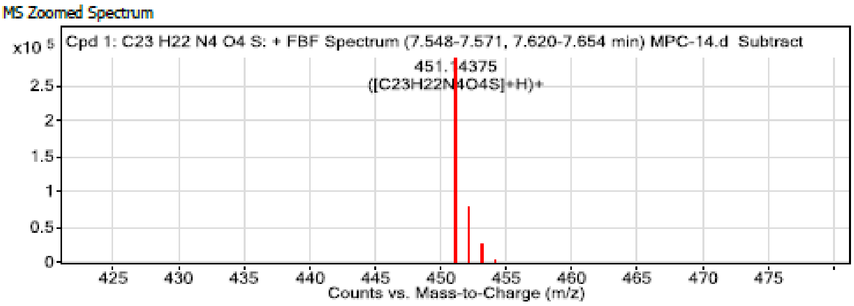

Figure S54. HRMS spectrum of compound **11**.

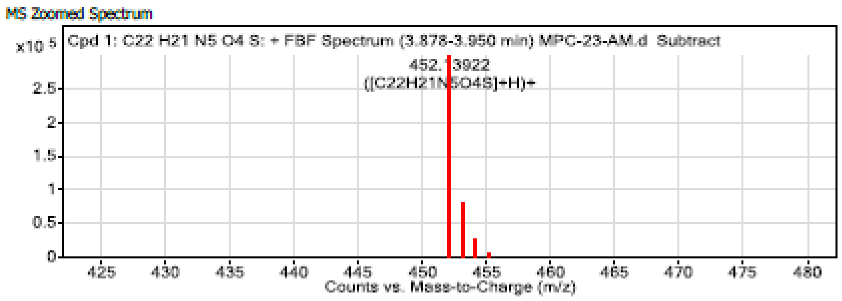

Figure S55. HRMS spectrum of compound **12**.

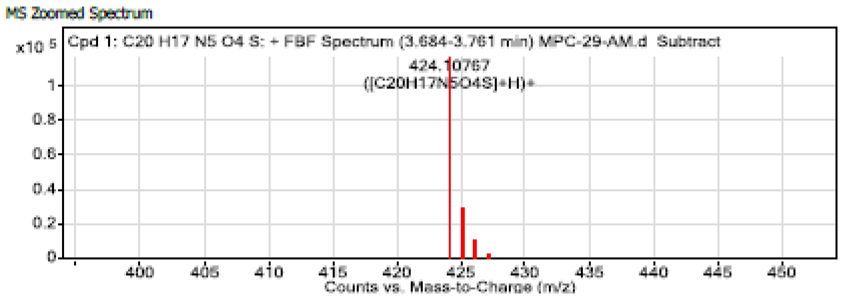

Figure S56. HRMS spectrum of compound **13**.

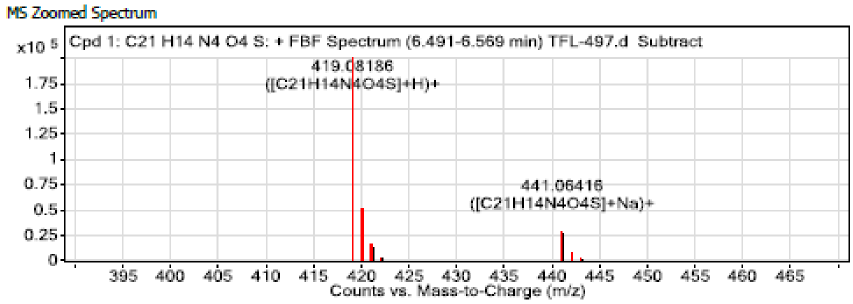

Figure S57. HRMS spectrum of compound **14**.

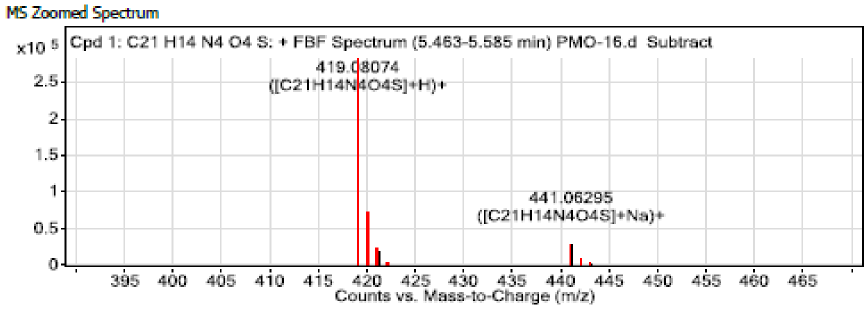

Figure S58. HRMS spectrum of compound **15**.
